# Photosystem stoichiometry adjustment is a photoreceptor-mediated process in *Arabidopsis*

**DOI:** 10.1038/s41598-022-14967-4

**Published:** 2022-06-29

**Authors:** Iskander M. Ibrahim, Steven D. McKenzie, Jae Chung, Uma K. Aryal, Walter D. Leon-Salas, Sujith Puthiyaveetil

**Affiliations:** 1 Department of Biochemistry and Center for Plant Biology, Purdue University, West Lafayette, IN 47907 USA; 2 Department of Comparative Pathobiology, Purdue University, West Lafayette, IN 47907 USA; 3 Bindley Bioscience Center, Purdue University, West Lafayette, IN 47907 USA; 4 School of Engineering Technology, Purdue University, West Lafayette, IN 47907 USA

**Keywords:** Photosystem I, Light responses

## Abstract

Plant growth under spectrally-enriched low light conditions leads to adjustment in the relative abundance of the two photosystems in an acclimatory response known as photosystem stoichiometry adjustment. Adjustment of photosystem stoichiometry improves the quantum efficiency of photosynthesis but how this process perceives light quality changes and how photosystem amount is regulated remain largely unknown. By using a label-free quantitative mass spectrometry approach in *Arabidopsis* here we show that photosystem stoichiometry adjustment is primarily driven by the regulation of photosystem I content and that this forms the major thylakoid proteomic response under light quality. Using light and redox signaling mutants, we further show that the light quality-responsive accumulation of photosystem I gene transcripts and proteins requires phytochrome B photoreceptor but not plastoquinone redox signaling as previously suggested. In far-red light, the increased acceptor side limitation might deplete active photosystem I pool, further contributing to the adjustment of photosystem stoichiometry.

## Introduction

In natural growth habitats of plants, sunlight varies substantially for photosynthesis in both quantity and quality. Some of the variations in sunlight are predictable diurnal or seasonal changes while others are unpredictable as caused by, for example, cloud cover or surrounding vegetation. Enrichment of photosystem II (PSII)-specific short wavelength or photosystem I (PSI)-specific long wavelength light in natural growth habitats of plants causes an imbalance in the photosynthetic electron transport by preferential excitation of one photosystem over the other. One way in which plants counteract this imbalance is by photosystem stoichiometry adjustment, wherein the relative abundance of the two photosystems becomes adjusted to suit the prevailing light quality condition^1^. This acclimatory response, which requires a half-time of approximately 20 h^2^, improves the quantum efficiency of photosynthesis^1^. Regulation of the PSI amount seems to be the principal mechanism by which the photosystem stoichiometry is adjusted in plants, algae, and cyanobacteria^3–5^. Growth under pure far-red light has recently been shown to increase the PSII to PSI ratio in *Arabidopsis* to as much as 5.0 by decreasing the PSI content^6^. There are, however, conflicting reports on the contribution of PSII to plant photosystem stoichiometry adjustment. In pea, per chlorophyll, PSII reaction center content is higher in PSI light and lower in PSII light^1^ while in *Arabidopsis*, PSII content does not seem to vary with light quality when analyzed on an equal chlorophyll basis^7^. The abundance of the cytochrome *b*_6_*f* (cyt *b*_6_*f*) complex, which bridges the two photosystems in series for a linear electron transport from water to NADP^+^, seems to respond to light quality in *Arabidopsis* but not in pea when its concentration was normalized to total chlorophyll^1,7^. Whether light quality affects the accumulation of ATPase is currently unknown and so is the levels of NAD(P)H dehydrogenase complex (NDH) that equips PSI for a cyclic electron transport pathway^8^.

For photosystem stoichiometry adjustment, an important question has been how are light quality variations perceived by plants? Earlier work suggested the involvement of phytochrome photoreceptor^9^ but more recent work has however raised the possibility of chloroplasts themselves acting as light quality sensors wherein the unequal excitation of photosystems by light quality is monitored as a change in the redox state of the inter-photosystem electron carrier plastoquinone (PQ)^10^. PQ pool redox state governs the transcription of chloroplast-encoded reaction center genes but whether this leads to adjustment of photosystem stoichiometry has never been demonstrated^5,10,11^. Our recent work has revealed direct transcriptional control of chloroplast PSI reaction center gene *psaA* by light quality^12^. This regulation requires phytochrome B (phyB)-mediated expression of nuclear-encoded sigma factor 1 (SIG1), a major transcription initiation factor for *psaA*^12^. To further clarify the nature of plant thylakoid proteomic response under light quality and the role of light and redox signals in this process, here we examine the proteomic and genetic responses of a light and a redox signaling mutant of *Arabidopsis*. The redox state of the PQ pool and PSI reaction center are also probed to identify the basis of light quality acclimation of plant thylakoids.

## Results

### Light quality-driven thylakoid proteomic response consists mostly of phytochrome-dependent regulation of PSI amount

**Figure 1:**
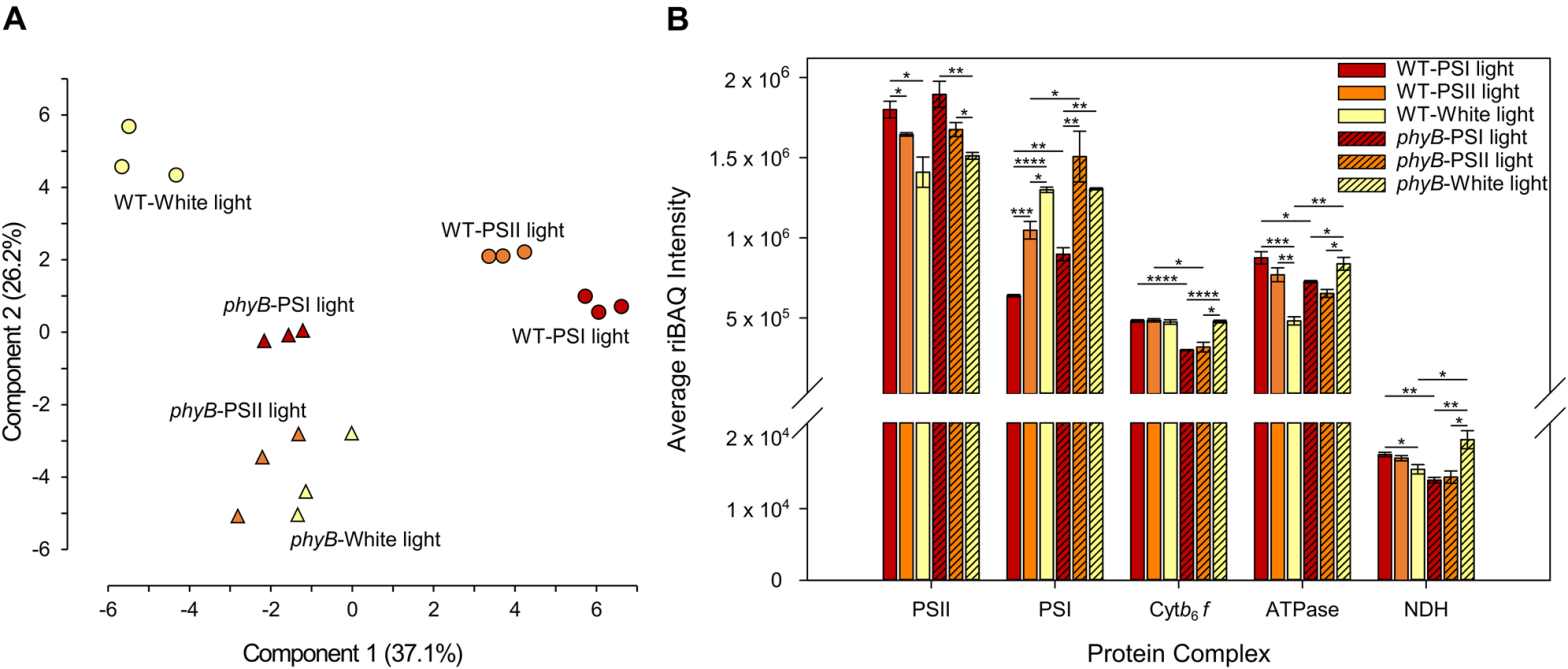
Comparison of light quality-responsive thylakoid proteome and protein complex abundance in wild type and *phyB* mutant. (**A**) A principal component analysis of wild type and *phyB* thylakoid proteome samples using riBAQ values of 476 shared plastid proteins. Light quality condition is indicated alongside each data set. (**B**) Abundance of five major thylakoid protein complexes in wild type and *phyB* under different light quality conditions as calculated from mean riBAQ intensities of their constituent subunits. Error bars represent SEM of three biological replicates. Statistical significance (p-value) is denoted by *, < 0.05; **, 0.01; ***, 0.001; ****, 0.0001. For a full list of all proteins detected in LC–MS/MS analysis, the 476 plastid proteins used for PCA, and for subunits used for thylakoid protein complexes abundance calculation, see Supplementary Tables S1, S2 and S3.

To study the light quality-responsive thylakoid proteome, four-week-old white light-grown wild type and *phyB* mutant *Arabidopsis* plants were acclimated to PSI light or PSII light for four days while a third set of plants remained in the white growth light. Thylakoids were isolated from all three sets of plants and thylakoid proteins were subjected to LC–MS–MS analysis. Mass spectra were searched against *Arabidopsis* proteome database and a total of 1000 proteins were identified to be commonly present in all 18 samples (Supplementary Table S1). A filtering for chloroplast location identifies 476 common proteins (Supplementary Table S2). For label-free quantification of protein abundance between samples, we used the relative intensity-based absolute quantification (riBAQ) method. riBAQ is a normalized measure of molar abundance of individual proteins that is ideal for comparison between different tissues and genotypes^13^. It is calculated by dividing each protein’s iBAQ value by the sum of all unfiltered iBAQ values in the corresponding sample. Figure 1A shows a scatter plot of samples after a principal component analysis of riBAQ values of the 476 shared plastid proteins. Wild type samples cluster into three distinct groups, corresponding to the three light treatments, with the white light group well separated from the PSII and PSI clusters. Variation between biological replicates is fairly small as seen in the close clustering of data points. The three sets of *phyB* thylakoid proteomes, in contrast, seem less spread out than wild type with the white and PSII light samples showing some overlap (Fig. 1A).

With their high abundance, the electron and proton transport complexes make up most of the plant thylakoid proteome. PSII is the most abundant complex, followed by PSI, ATPase, cyt *b*_6_*f*, and NDH (Fig. 1B). To determine how light quality affects the accumulation of these complexes, we quantified their abundance from riBAQ values (Fig. 1B). A total of 57 subunits were chosen for this calculation (Supplementary Table S3), and as described earlier^14^, the abundance of each complex is the mean riBAQ value of its constituent subunits. Photosystem abundance was calculated from the values of reaction center core subunits to account for the heterogeneity in antenna composition and the differing antenna-core stoichiometry. The most conspicuous change in light quality in wild type was a ~ 40% reduction in PSI amount in PSI light relative to the level found in PSII light (Fig. 1B). PSII abundance also varied with light quality but at a modest ~ 9% higher in PSI light compared to PSII light. For cyt *b*_6_*f*, ATPase, and NDH, there is statistically no significant change in abundance between the two light quality treatments. In white growth light, the pattern of photosystem accumulation in wild type plants mirrors that of PSII light but with a further ~ 20% boost in PSI level. Amounts of cyt *b*_6_*f* and NDH in white light were similar to those in photosystem-specific light quality conditions. To further examine the direction of PSII, PSI, and cyt *b*_6_*f* abundance changes, the concentrations of these complexes per chlorophyll were determined by difference absorption spectroscopy of thylakoids isolated from nine-week-old short-day (8 h light/16 h dark) grown wild type plants before and after light switch. Table 1 shows concentration of PSII, PSI, and cyt *b*_6_*f* per mol of chlorophyll before (in white light, zero time) and four days after switch to PSII, PSI, and white light. PSII concentration decreases by ~ 10% in PSII light when compared to the before switch white light sample while growth in other light conditions causes no significant changes in PSII level. PSI amount declines by ~ 5 and ~ 23% in PSII and PSI light, respectively, relative to the level found before the light switch. Cyt *b*_6_*f* complex, likewise, becomes diluted by ~ 13 and ~ 30% after being moved to PSII and PSI light respectively (Table 1). These light quality-driven changes in the abundance of PSII, PSI, and cyt *b*_6_*f* are in line with our proteome data (Fig. 1B) and with a previous report that quantified these complexes per chlorophyll in *Arabidopsis*^7^. However, the decrease of PSI in PSI light seems less pronounced when expressed per chlorophyll (Table 1) than per protein (Fig. 1B). A normalization by chlorophyll also finds differences in cyt *b*_6_*f* abundance (Table 1) in contrast to the unchanged abundance reported by the quantitative proteomics (Fig. 1B). Since not all thylakoid protein complexes bind chlorophyll or bind it equally, chlorophyll normalization is likely to introduce artifacts in thylakoid protein complex estimation.

**Table 1:** Concentration of PSII, PSI, and Cyt *b*_6_*f* per mol of chlorophyll.

Complex (mmol/mol Chl)	White light (before switch)	PSII light (after 4 days)	PSI light (after 4 days)	White light (after 4 days)
PSII (cyt *b*_559_)	2.28 ± 0.06	2.04 ± 0.04*	2.29 ± 0.06	2.21 ± 0.02
PSI (P700)	2.58 ± 0.04	2.44 ± 0.03*	2.0 ± 0.06***	2.62 ± 0.004
Cyt *b*_6_*f* (cyt *b*_6_ and cyt *f*)	0.86 ± 0.01	0.75 ± 0.03*	0.60 ± 0.06*	0.81 ± 0.013

The relevant chromophore used for the estimation of each complex is given in parentheses in column 1. Statistically significant differences between the before switch sample and light quality treatments are inferred from a Student’s t-test at alpha level *p < 0.05 and ***p < 0.001. ± denotes standard error of mean of three biological replicates.

The aberrant light quality proteomic response of *phyB* mutant (Fig. 1A) was also evident in its abundance of electron transport complexes (Fig. 1B). PSII levels in *phyB* were similar to corresponding wild type light quality samples and *phyB* PSI level varied expectedly with a lower accumulation in PSI light. However, *phyB* mutant accumulated nearly 30% more PSI in both PSI and PSII light conditions compared to wild type. Like that in wild type, the abundance of cyt *b*_6_*f*, ATPase, and NDH in *phyB* was insensitive to photosystem-specific light quality conditions but there was a general decline in these complexes in the mutant relative to wild type. The white fluorescent growth light produced a PSII light-like effect in *phyB* as in wild type, with the levels of PSII, PSI, and cyt *b*_6_*f* in *phyB* being similar to wild type white light samples. The abundance of ATPase and NDH was however higher in *phyB* in white light compared to wild type. The PSII to PSI ratio in wild type increased from nearly 1.1 in white light to 2.8 in PSI light as a result of the light quality acclimation (Table 2). This increase parallels with a decline in PSI amount as plants move from white light to PSI light. PSII light-grown wild type plants have an intermediate photosystem ratio of 1.6. Changes in thylakoid protein complex stoichiometry appear to be primarily driven by changes in PSI amount though there is some contribution from ATPase in white light (Table 2). Consistent with an overaccumulation of PSI, the 2.1 and 1.1 PSII to PSI ratios of *phyB* in respectively PSI and PSII light conditions are lower than corresponding wild type samples. Interestingly, *phyB* has a PSII:PSI ratio of 1.16 also in white light. Thus, the overlap in the proteomes of *phyB* PSII and white light samples (Fig. 1A) is also reflected in their identical PSII:PSI ratios. In *phyB*, ratios of non-photosystem complexes have also deviated slightly from wild type ratios in all three light conditions likely due to differences in the accumulation of these complexes (Table 2).

**Table 2:** Stoichiometries of major thylakoid protein complexes in wild type and *phyB*.

	PSII	PSI	Cyt *b*_6_*f*	ATPase	NDH
**WT-PSI light**					
PSII	1 ± 0	2.82 ± 0.09	3.75 ± 0.1	2.06 ± 0.03	98.23 ± 1.08
PSI	0.36 ± 0.01	1 ± 0	1.33 ± 0.01	0.73 ± 0.03	34.88 ± 0.79
Cyt *b*_6_*f*	0.27 ± 0.01	0.75 ± 0.01	1 ± 0	0.55 ± 0.02	26.25 ± 0.52
ATPase	0.49 ± 0.01	1.37 ± 0.06	1.82 ± 0.07	1 ± 0	47.71 ± 1.21
NDH	0.01 ± 0	0.03 ± 0	0.04 ± 0	0.02 ± 0	1 ± 0
***phyB*-PSI light**					
PSII	1 ± 0	2.12 ± 0.08	6.35 ± 0.31	2.61 ± 0.09	130.36 ± 8.73
PSI	0.47 ± 0.02	1 ± 0	3.01 ± 0.17	1.24 ± 0.05	61.57 ± 3.07
Cyt *b*_6_*f*	0.16 ± 0.01	0.33 ± 0.02	1 ± 0	0.41 ± 0.01	20.51 ± 0.72
ATPase	0.38 ± 0.01	0.81 ± 0.03	2.43 ± 0.04	1 ± 0	49.86 ± 1.92
NDH	0.01 ± 0	0.02 ± 0	0.05 ± 0	0.02 ± 0	1 ± 0
**WT-PSII light**					
PSII	1 ± 0	1.58 ± 0.1	3.4 ± 0.08	2.16 ± 0.14	92.36 ± 2.35
PSI	0.64 ± 0.04	1 ± 0	2.16 ± 0.1	1.36 ± 0.01	58.67 ± 2.23
Cyt *b*_6_*f*	0.29 ± 0.01	0.47 ± 0.02	1 ± 0	0.63 ± 0.03	27.19 ± 0.25
ATPase	0.47 ± 0.03	0.73 ± 0	1.59 ± 0.08	1 ± 0	43.08 ± 1.81
NDH	0.01 ± 0	0.02 ± 0	0.04 ± 0	0.02 ± 0	1 ± 0
***phyB*-PSII light**					
PSII	1 ± 0	1.14 ± 0.13	5.35 ± 0.44	2.58 ± 0.17	111.65 ± 6.13
PSI	0.9 ± 0.12	1 ± 0	4.94 ± 1.04	2.3 ± 0.17	101.48 ± 15.86
Cyt *b*_6_*f*	0.19 ± 0.02	0.22 ± 0.04	1 ± 0	0.49 ± 0.06	20.99 ± 1.1
ATPase	0.39 ± 0.02	0.44 ± 0.03	2.1 ± 0.28	1 ± 0	43.58 ± 3.44
NDH	0.01 ± 0	0.01 ± 0	0.05 ± 0	0.02 ± 0	1 ± 0
**WT-white light**					
PSII	1 ± 0	1.08 ± 0.07	2.96 ± 0.11	2.92 ± 0.06	86.8 ± 4.45
PSI	0.93 ± 0.06	1 ± 0	2.74 ± 0.08	2.71 ± 0.15	80.37 ± 4.02
Cyt *b*_6_*f*	0.34 ± 0.01	0.37 ± 0.01	1 ± 0	0.99 ± 0.03	29.28 ± 1
ATPase	0.34 ± 0.01	0.37 ± 0.02	1.01 ± 0.03	1 ± 0	29.65 ± 0.92
NDH	0.01 ± 0	0.01 ± 0	0.03 ± 0	0.03 ± 0	1 ± 0
***phyB*-white light**					
PSII	1 ± 0	1.16 ± 0.01	3.17 ± 0.1	1.81 ± 0.1	74.32 ± 5.75
PSI	0.86 ± 0.01	1 ± 0	2.74 ± 0.06	1.57 ± 0.08	64.11 ± 4.43
Cyt *b*_6_*f*	0.32 ± 0.01	0.37 ± 0.01	1 ± 0	0.57 ± 0.03	23.41 ± 1.49
ATPase	0.55 ± 0.03	0.64 ± 0.03	1.76 ± 0.09	1 ± 0	40.88 ± 1.2
NDH	0.01 ± 0	0.02 ± 0	0.04 ± 0	0.02 ± 0	1 ± 0

± represents standard error of mean of three biological replicates.

### PSI protein and transcript accumulation under changing light quality is PQ pool redox-independent but requires phyB photoreceptor

**Figure 2:**
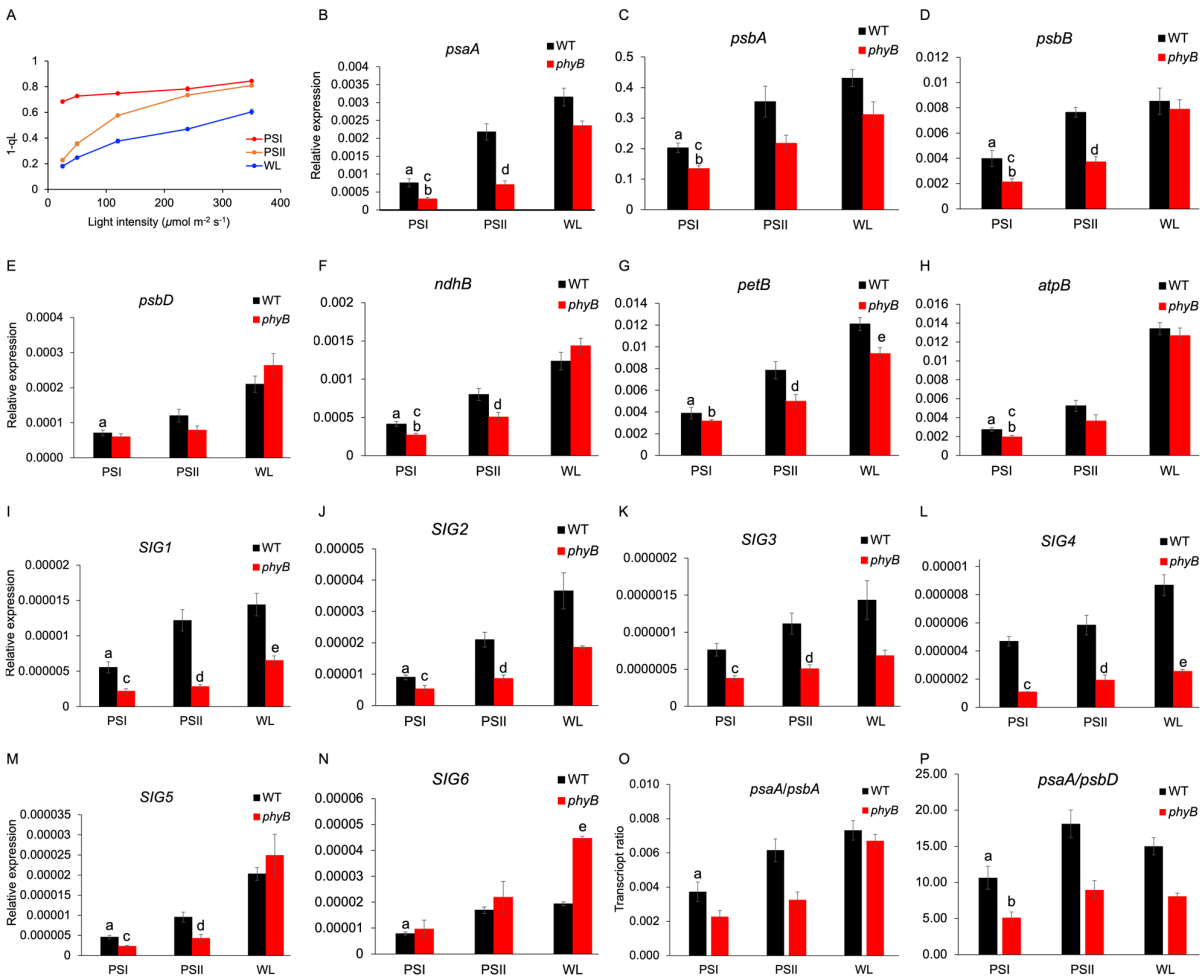
PQ pool reduction level and abundance of selected gene transcripts in light quality-acclimated wild type and *phyB* mutant. (**A**) PQ pool reduction level in wild type plants acclimated to 4-days of PSI, PSII, and white light (WL) as indicated by the 1-qL parameter. Data points represent mean 1-qL values of four biological replicates measured at different actinic light intensities. Error bars represent ± SEM. (**B**–**P**) Abundance of selected plastid (**B**–**H**) and nuclear sigma factor (**I**–**N**) gene transcripts and *psaA*:*psbA* and *psaA*:*psbD* transcript ratios (**O**,**P**) in light quality-acclimated wild type and *phyB* plants. Data are represented as mean ± SEM of three biological replicates. Statistical significance (p-value < 0.05) for the following pairwise sample comparison is denoted by a, wild type PSI vs PSII light; b, *phyB* PSI vs PSII light; c, wild type PSI vs *phyB* PSI light; d, wild type PSII vs *phyB* PSII light; e, wild type white light vs *phyB* white light. For a complete list of p-values see Supplementary Table S5.

The characteristic decrease of PSI in PSI light (Fig. 1B) could arise from a suppression of its de novo biosynthesis or from an increased degradation. Since PQ pool redox signal is a regulator of PSI gene expression^10,11^, we examined whether PQ pool redox state indeed corresponds with PSI protein and transcript accumulation under light quality. After 4-days of acclimation, interestingly, PQ pool becomes over-reduced in PSI light-grown plants and over-oxidized in PSII light-grown wild type plants (Fig. 2A). Increase in the intensity of the white actinic light, used for driving photosynthesis during the measurement, progressively reduces the quinone pool in all three plants as expected (Fig. 2A). These long-term light quality-driven changes in PQ pool redox state are however incongruent with trends in accumulation of PSI or its reaction center gene transcript *psaA* (Figs. 1B, 2B). A reduced quinone pool has been suggested to signal for an increased *psaA* transcription^10^ and yet PSI protein and transcript abundance is lower in PSI light, under which PQ pool becomes over-reduced. A similar mismatch is also apparent for PSII light (Figs. 1B, 2B). On a shorter timescale of minutes, our PSI and PSII lights indeed oxidize and reduce the PQ pool, respectively, as shown by their ability to induce state transitions (Supplementary Fig. S2). The discrepancy between long-term light quality illumination and PQ pool redox state has been noted earlier using both white and orange actinic lights but it has been suggested that the greater amount of PSI in PSII light-acclimated plants oxidizes the pool and the lower amount of PSI in PSI light-acclimated plants, keeps the pool reduced^15,16^. This suggestion however does not account for the continued regulation of *psaA* transcript accumulation under long-term light quality-illumination (Fig. 2B), leaving open the possibility that light quality signals for photosystem stoichiometry adjustment are perceived independently of PQ pool. Chloroplast Sensor Kinase (CSK) is a bacterial-type sensor kinase with a role in linking PQ pool redox state with *psaA* gene expression, making it an ideal candidate for regulating plant photosystem stoichiometry^17,18^. However, the thylakoid protein complex abundance and stoichiometry of a *csk* knockout mutant are similar to wild type (Supplementary Fig. S3 and Supplementary Table S4). It is thus likely that the PQ or CSK redox signaling pathway is not part of plant photosystem stoichiometry adjustment and that redox control of chloroplast gene expression has an as-yet-undetermined function.

To examine whether light quality provides input into PSI biogenesis via phytochrome, we analyzed expression of plastid genes in *phyB* mutant. Figure 2B–H shows transcript levels of selected PSI (*psa*), PSII (*psb*), NDH (*ndh*), cyt *b*_6_*f* (*pet*), and ATPase (*atp*) genes after 4-days of acclimation in PSI, PSII, and white light. Wild type plants respond to PSI light with a statistically significant decrease (p-value < 0.05; Supplementary Table S5) in the abundance of all transcripts relative to PSII light, with the transcript abundance in white light being higher or equal to that of PSII light. Compared to wild type, *phyB* shows a lower abundance of *psaA*, *psbA*, *psbB*, *ndhB*, and *atpB* in PSI light and *psaA*, *psbB*, *petB*, and *ndhB* in PSII light, with the *psaA*, *psbB*, and *ndhB* levels affected in both light conditions. The *phyB* white light sample has similar levels of transcripts as the wild type white light sample, except for a slightly lower *petB*. Between PSI and PSII lights in *phyB*, there is a small but statistically significant change for all transcripts with the exception of *psbD*. Since the transcription of plastid genes depends on the abundance of their respective sigma factors, we checked whether the expression of nuclear sigma factor genes shows any response to light quality. As with plastid transcripts, PSI light lowers the accumulation of all sigma factor gene transcripts except that of *SIG3* and *SIG4* in wild type but this response is lost in *phyB* (Fig. 2I–N). All *SIG* transcripts except *SIG6* are present in lower amount in *phyB* in both PSI and PSII light when compared with wild type. Of all plastid genes analyzed in wild type the effect of light quality change seems most pronounced for *psaA*, with its transcript declining nearly threefold in PSI light (Fig. 2B). This preferential decrease of *psaA* is also evident in its ratios to *psbA* and *psbD* (Fig. 2O,P). Furthermore, light quality-driven expression of *psaA* depends on phyB-mediated transcription of *SIG1* as the loss of light quality responsiveness of *SIG1* in *phyB* (Fig. 2I) results in unchanged *psaA*:*psbA* ratios in the mutant (Fig. 2O). The phyB-mediated expression of SIG1 thus likely accounts for the light quality-responsive *psaA* transcription reported here (Fig. 2B) and elsewhere^10,19^.

### Far-red light acclimation involves phyB-dependent downregulation of proteins involved in PSI assembly, chloroplast translation, and chlorophyll biosynthesis

**Figure 3:**
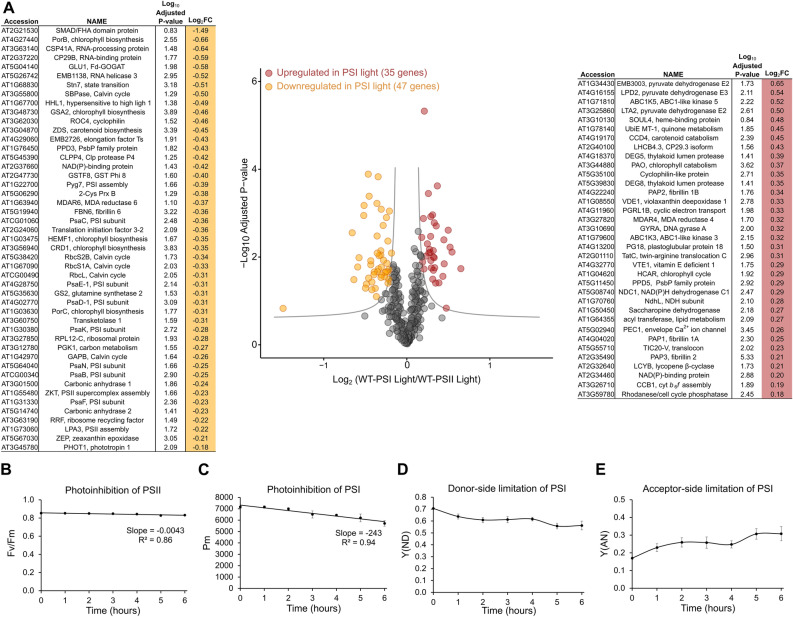
Differentially accumulating thylakoid proteins and differing photosystem susceptibilities in wild type under light quality conditions. (**A**) A volcano plot of differentially accumulating thylakoid proteins in a comparison of wild type PSI vs PSII light samples. The y-axis contains the negative log_10_-adjusted p-values and the x-axis, log_2_ up or down fold change in protein accumulation. The cut-off lines denote statistically significant changes (ANOVA followed by two-tailed t-test, p-value < 0.05). Differential expression analysis was performed using the Perseus software platform and results were plotted in R using the ggplot package. The list of downregulated and upregulated proteins in PSI light are given on the left and right of the plot, respectively. The lists contain gene accessions, latest annotations on protein function, -log_10_-adjusted p-values, and log_2_ fold changes. Each data point is the mean riBAQ value of three biological replicates. (**B**–**E**) PSII and PSI maximal photochemical efficiencies under PSI light illumination. F_v_/F_m_ and P_m_ values as a measure of photoinhibition of PSII (**B**) and PSI (**C**), respectively, in far-red light. The donor (**D**) and acceptor (**E**) side limitation of PSI are also plotted at time points before (0 time) and after incubation in PSI light. Each data point is mean ± SEM with n = 4 biological replicates.

To further understand the basis of photoregulation of PSI content by phytochrome, we examined whether our larger set of thylakoid light quality proteome data contain any potential regulatory proteins especially since the plastid gene expression machinery is tethered to the thylakoid membrane in mature chloroplasts^20,21^. Figure 3A shows a volcano plot of differentially expressed thylakoid proteins in wild type PSI light vs PSII light samples. The differential accumulation of all thylakoid proteins, except STN7, is phyB-dependent (Supplementary Fig. S4). The down-regulated proteins in PSI light (or upregulated in PSII light) include seven subunits of PSI, including the reaction center protein PsaB, as consistent with the decrease of PSI in far-red light. Remarkably, down-regulated proteins also include Pyg7, an essential assembly factor for algal and plant PSI^22,23^. The PsbP family protein PPD3 is another potential PSI assembly factor as its paralogue PPD1 has been implicated in PSI assembly^24^. The phyB-mediated downregulation of these assembly factors may thus slow PSI assembly, leading to a decline in PSI content in far-red light. Several RNA-binding proteins are also down-regulated but it is unclear whether they are specific for PSI mRNAs. Other possible regulators include translation elongation factor EF-Ts, translation initiation factor 3, and a ribosome recycling factor. A homologue of initiation factor 3, intriguingly, controls chromatic acclimation in cyanobacteria^25^. The ribosomal protein L12-C and the RNA Helicase 3 (RH3), which is required for chloroplast ribosome biogenesis^26^, are also downregulated as indicative of a phyB-mediated general decrease in plastid translation under far-red light. Multiple chlorophyll biosynthetic enzymes, including the exclusively thylakoid-bound PorB and PorC^27^, accumulate less in PSI light as suggestive of a phyB-driven generalized decrease in chlorophyll biosynthesis. Intriguingly, the gene expression of some of these chlorophyll enzymes has already been shown to be under phytochrome control^28^. Proteins that accumulated in PSI light (or down-regulated in PSII light) include the rare Lhcb4.3 isoform (Lhcb8), which is known to be induced by far-red light and high light^29,30^, and several enzymes of carbon metabolism. The higher abundance of violaxanthin deepoxidase 1 (VDE1) (Fig. 3A) is consistent with the recent report of a higher nonphotochemical quenching in far-red-acclimated *Arabidopsis* plants^6^.

### Far-red light illumination causes a decrease in photochemically active PSI pool

To examine whether photodamage is a further contributing factor for the decrease of PSI in PSI light, we measured the photochemically active pool of photosystems in 4-week old wild type plants illuminated with the same low intensity PSI light that was used in the long-term light quality experiment. Figure 3B shows active pool of PSII upon transfer of white light-grown plants to PSI light as measured by the chlorophyll fluorescence parameter F_v_/F_m_. The F_v_/F_m_ decreases only slightly after 6 h of PSI light relative to the value in white light (zero time). PSI, in contrast, shows a much larger decline in activity in PSI light as revealed by the maximum photooxidizable P700 (P_m_) (Fig. 3C). We further find that this decrease arises from the acceptor side limitation of PSI (Fig. 3E), as consistent with a decreased utilization of reductants downstream^31^. An increased acceptor side limitation may lead to heightened photoinactivation of reaction centers, which in turn could result in increased degradation of PSI in far-red illumination of PSI light.

## Discussion

Ever since the first description of plant photosystem stoichiometry adjustment, the precise nature of this acclimation and the regulatory pathway governing it have eagerly been sought. Our proteome and spectroscopic analyses reveal a major light quality-responsive abundance change in PSI but not PSII (Fig. 1B and Table 1). This observation is consistent with earlier reports and underlines the suggestion that it is mostly the regulation of PSI amount that adjusts the photosystem stoichiometry^3–5^. Analysis of thylakoid proteomes of *phyB* and *csk* knockout mutants, and the redox state of PQ pool under prolonged light quality illumination strongly suggests that regulation of PSI amount and photosystem stoichiometry requires the phytochrome photoreceptor but not PQ pool redox signal or CSK (Figs. 1B, 2A, Supplementary Fig. S3, Table 2, and Supplementary Table S4). To further ascertain the role of phytochrome in photosystem stoichiometry adjustment, we estimated phytochrome photoequilibrium (PPE) of our PSII, PSI, and white fluorescent light sources based on their photon flux distribution (Supplementary Fig. S1) and the photochemical cross sections (σ) of P_r_ and P_fr_ as given in^32^. PPE is the ratio of active phytochrome (P_fr_) to the total phytochrome (P_r_ + P_fr_) and is often used to predict the phytochrome response under a given light source. From the PPE values given in Table 3 it is readily apparent that the PSII and white fluorescent light sources have a higher ratio of active phytochrome than the PSI light source. An additional measure of the red to far-red ratio (R/FR) of each light source further agrees with its PPE value. With a relatively higher R/FR ratios, both PSII and white fluorescent lights are likely to favor an active phytochrome population while the PSI light, with its lower R/FR ratio, may produce a less active pool of phytochromes. Remarkably, the PSII light-like effect of white fluorescent illumination on thylakoid proteome and photosystem abundance could now be attributed to the action spectrum of phytochrome. With a high PPE and R/FR ratio, the white fluorescent light should certainly behave like the PSII light. The emission spectrum of the white fluorescent lamp indeed mimics the PSII light source, containing little of the long wavelength component but the white fluorescent light is bluer (Supplementary Fig. S1) with an order of magnitude higher intensity (see “Methods”), a difference that likely causes a lower level of ATPase in white light-grown plants (Fig. 1B). It should be noted that the 1.1 PSII:PSI ratio estimated here for wild type plants in white light is lower than the ~ 1.6 ratio that we reported earlier^14^. However, plants for the current study are grown on a long-day condition and at a lower light intensity. The active P_fr_ form of phytochrome certainly accumulates under long-day condition^33^, which may then promote increased PSI synthesis and as a result a lower PSII to PSI ratio (Fig. 1B, Table 2). Photosystem stoichiometry might also vary in plant development as the difference absorption spectroscopy of cytochromes and P700 in older plants (Table 1) seems to suggest a nearly equimolar ratio of PSII and PSI even though these plants were grown under a short-day condition. Whether photosystem stoichiometry indeed varies with age in a phytochrome-dependent manner in *Arabidopsis* remains to be seen.

**Table 3:** PPE and R/FR ratios of light sources.

Illumination condition	Phytochrome photoequilibrium (P_fr_/(P_r_ + P_fr_))	R/FR
PSII light	0.87	3.8
White light	0.83	3.5
PSI light	0.70	2.0

The depletion of photochemically active PSI pool under PSI light is indicative of photoinactivation and degradation playing a role in downregulating PSI amount (Fig. 3B). This degradation acting in conjunction with a phyB-mediated decrease in PSI gene transcription and assembly specifically, and chloroplast translation and chlorophyll biosynthesis generally may bring about a decline in the steady state level of PSI in far-red light (Figs. 2, 3). The exact pathways that produce the over accumulation of PSI in *phyB* mutant remain to be investigated as the constitutive lack of this major phytochrome photoreceptor has other consequences for thylakoid proteome (Fig. 1B, Supplementary Fig. S5, and Supplementary Tables S6 and S7). It is also possible that phytochrome works cooperatively with blue light photoreceptors in determining thylakoid protein composition^34^. Enrichment of far-red light is pervasive under natural growth habitats of plants due to shading. Far-red light, with its preferential excitation of the so-called “red” chlorophylls of PSI^35^, acts as a PSI light. The low intensity solar radiation under cloudy days on the other hand mimics PSII light with its higher blue to red ratio, which may preferentially excite the Soret and Q_y_ band regions of chlorophyll *b* (Supplementary Fig. S1)^36^. In summary, our data reveal that the regulation of PSI amount forms the major thylakoid proteomic response under light quality, that this response serves to adjust photosystem stoichiometry, and that the regulation of PSI content by light quality is a photoreceptor-mediated rather than a plastoquinone redox-controlled process.

## Methods

### Plant materials and growth conditions

*Arabidopsis thaliana* (col-0) wild type, *phyB* null mutant (*phyB*-*9*), and *csk* knockout mutant (SALK_027360) plants were grown from seeds on soil at 21 °C. The white growth light was supplied with Philips F40T8/TL841 strip lamps giving ~ 80 µmol photons m^−2^ s^−1^. PSI light was provided with Narva 18 W/015 red fluorescent strip lamps wrapped with a layer of plasa red filter (LEE 029). White fluorescent lamps (Philips Master TL-D 18 W/827) covered with a layer of burnt yellow filter (LEE 770) produced PSII light. Photon flux density at the leaf level was ~ 6 µmol photons m^−2^ s^−1^ in PSI light and ~ 12 µmol photons m^−2^ s^−1^ in PSII light. For plants used for the analysis of thylakoid proteome and plastid gene transcript, all three lights were provided on a long day condition (16 h light/8 h dark). Plants used for the measurement of state transitions, photoinhibition, and protein complex concentration per chlorophyll were 6–9-week-old and grown under a short day condition (8 h light/16 h dark) at a white growth light intensity of ~ 150 µmol photons m^−2^ s^−1^. The spectral irradiance under different plant growth conditions is recorded by a wavelength-calibrated Black Comet spectroradiometer (StellarNet). Mutant *Arabidopsis* plants were grown and plant materials disposed in accordance with Purdue’s Institutional policy on genetically modified organisms.

### Thylakoid proteomics

Thylakoid membranes were extracted from the leaves of 35-days old light quality-acclimated plants as described in Ref.^14^. Thylakoid protein extraction and peptide sample preparation were performed essentially as in Ref.^14^. Samples were analyzed by reverse-phase LC–MS/MS using the Dionex UltiMate 3000 RSLC nano System coupled to an Orbitrap Fusion Lumos Tribid Mass Spectrometer (Thermo Fisher Scientific). Sample run, peptide detection, and quantification were done as described in detail in Ref.^14^ with no modifications.

### Quantitative real-time PCR

Total RNA was isolated from the leaves of 13-days old light quality-acclimated plants using the TRIzol reagent. RNA was treated with RNase-free DNase (NEB) to eliminate possible DNA contamination. qRT-PCR was performed with a one-step QuantiTech SRBR Green RT-PCR kit (Qiagen) in a StepOnePlus thermocycler (Applied Biosystems). The expression values of target genes were normalized to both total RNA and to housekeeping plastid gene *rrn16*. The relative changes in gene expression were analyzed by the 2^−ΔCt^ method. Primer pairs used are listed in Supplementary Table S8.

### Chlorophyll fluorescence and absorbance measurements

Room temperature variable fluorescence was acquired using a Hansatech pulse amplitude modulated (PAM) fluorescence monitoring system (FMS1) (Norfolk, England) with an emitter-detector unit. State 2 and state 1 respectively were induced with the same low intensity PSII and PSI lights used in the proteome analysis. The maximal fluorescence in state 2 (F_m_^'^2) and state 1 (F_m_^'^1) were recorded after a 0.8-s-long saturating white light pulse (13,000 µmol photons m^−2^ s^−1^). F_v_/F_m_ was calculated as $\frac{{F}_{m}-{F}_{0}}{{F}_{m}}$ and 1-qL^37^ as $1-\left(qP\frac{{F}_{0}^{^{\prime}}}{{F}_{s}}\right)$, where $qP= \frac{{F}_{m}^{^{\prime}}-{F}_{s}}{{F}_{m}^{^{\prime}}-{F}_{0}^{^{\prime}}}$.

The P700 redox kinetics was determined by difference absorption spectroscopy using a JTS-10 spectrophotometer. The redox state of P700 was monitored with a 705 nm probe light. To measure P_m_, leaf discs were illuminated with a 0.25-s-long 630 nm saturating pulse (2600 µmol photons m^−2^ s^−1^). A 5-s-long 630 nm continuous actinic light (940 µmol photons m^−2^ s^−1^), placed immediately after the saturating pulse, recorded the P parameter. A subsequent saturating pulse recorded the maximum photooxidizable P700 under actinic light (P_m_^'^). The P700 absorbance signal was corrected by the subtraction of a contaminating chlorophyll fluorescence signal, as determined by the measurement of the sample under saturating and actinic illumination without the probe light. The acceptor Y(NA) and donor Y(ND) side parameters were calculated as $\frac{{P}_{m}-{P}_{m}^{^{\prime}}}{{P}_{m}}$ and $\frac{P}{{P}_{m}}$, respectively^38^. PSII and PSI photoinhibition were monitored using F_v_/F_m_ and P_m_ parameters, respectively, under the same low intensity PSI light used in proteome and plastid gene transcripts analysis.

Cytochrome and P700 difference spectroscopy of thylakoid samples were performed with a Hitachi U-3900 and JTS-10 spectrophotometers, respectively, and as described earlier^14^.

### Estimation of phytochrome photoequilibrium and R/FR ratios

Phytochrome photoequilibrium (PPE) was calculated with the equation below using spectral photon distribution of each spectrum and photochemical cross sections of P_r_ (σ_r_) and P_fr_ (σ_fr_) taken from references^32,39^.

$$PPE= \frac{\sum_{\lambda =300 nm}^{\lambda =800 nm}{N}_{\lambda }{\sigma }_{{r}_{\lambda }}}{\sum_{\lambda =300 nm}^{\lambda =800 nm}{N}_{\lambda }{\sigma }_{{r}_{\lambda }}+ \sum_{\lambda =300 nm}^{\lambda =800 nm}{N}_{\lambda }{\sigma }_{{fr}_{\lambda }}}$$

${N}_{\lambda }$= the photon flux density at wavelength $\lambda$; ${\sigma }_{r\lambda }$= the photochemical cross section of P_r_ at wavelength $\lambda$ (m^2^ mol^−1^); ${\sigma }_{fr\lambda }$= the photochemical cross section of P_fr_ at wavelength $\lambda$^39^.

For the calculation of red to far-red ratios (R/FR) of each light source, the photon flux densities were integrated from area under wavelengths 620–699 nm for red and 700–750 nm for far-red from the corresponding spectrum and the red photon flux density was divided with the far-red density.

## Supplementary Information

Supplementary Information.

## Data Availability

The datasets from this study will be assigned a stable DOI and published on the Purdue University Research Repository (PURR): (https://purr.purdue.edu).
