# Supplementary material for: Photosystem stoichiometry adjustment is a photoreceptor-mediated process in Arabidopsis
Source: Sci Rep. 2022 Jun 29;12:10982. doi: 10.1038/s41598-022-14967-4 (PMC9243065; doi:10.1038/s41598-022-14967-4)
Supplement: Supplementary file 1 — Supplementary Information. [file 41598_2022_14967_MOESM1_ESM.pdf]

Photosystem stoichiometry adjustment is a photoreceptor-mediated process in *Arabidopsis*

Iskander M. Ibrahim, Steven D. McKenzie, Jae Chung, Uma K. Aryal, Walter D. Leon-Salas, Sujith Puthiyaveetil

Supplementary Figure 1

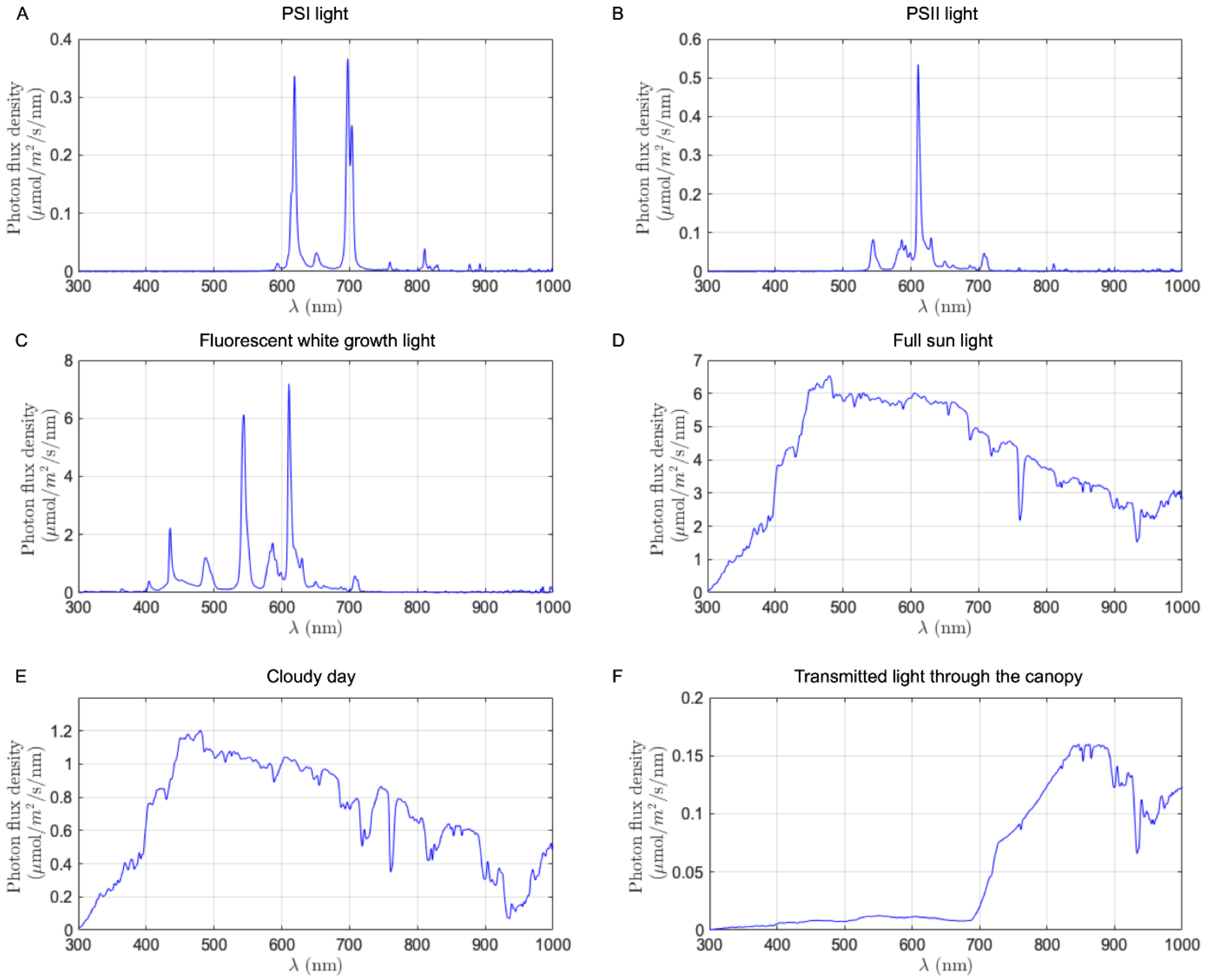

Supplementary Figure 1. Representative light emission spectra of some artificial and natural light sources

(**A-F**) The wavelength-calibrated spectral irradiance under PSI light (A), PSII light (B), fluorescent white growth light (C), full sunlight (D), cloudy day (E), and understory of canopy (F). The measured intensities of these light sources at plant height are ~6, 12, 150, 2000, 440, 3 μmol photons m^{-2} s^{-1}, respectively.

Supplementary Figure 2

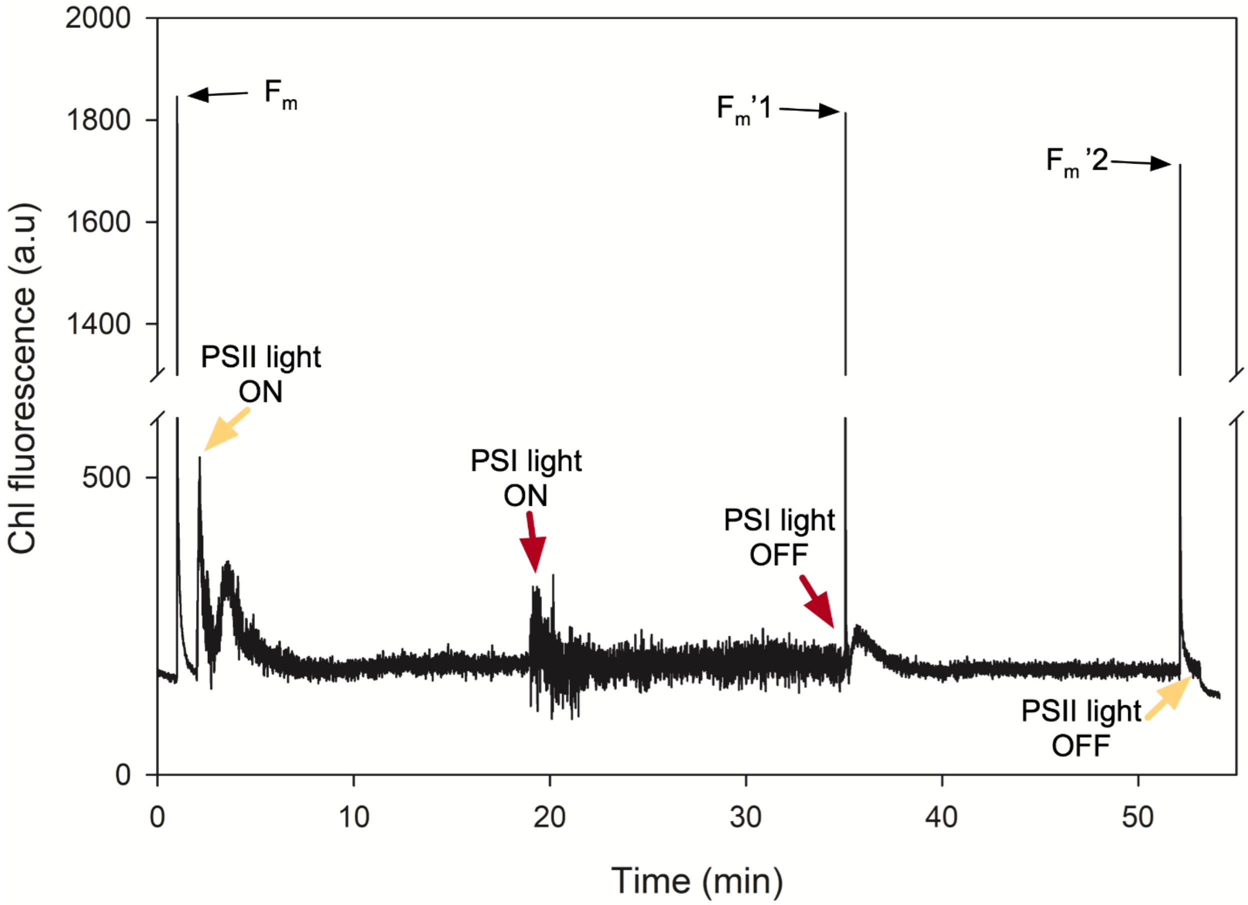

Supplementary Figure 2. Light state transitions as induced by PSII and PSI light sources

Changes in room temperature variable chlorophyll fluorescence yield indicate state 2 and state 1 transitions as induced by PSII and PSI lights, respectively. The time points at which PSII and PSI lights are on are indicated on the trace. F_m, $F_m'1$, and $F_m'2$ denote maximal fluorescence under dark, state 1, and state 2, respectively. Due to the lower intensities of the PSII and PSI light sources, the fluorescence signal tends to be jittery.

Supplementary Figure 3

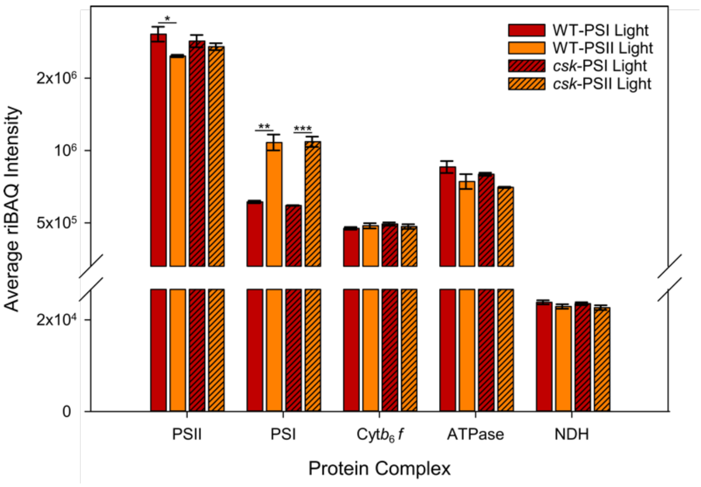

Supplementary Figure 3. Thylakoid protein complex abundance in wild type and *csk ko*

Abundance of major thylakoid protein complexes in wild type and *csk ko* mutant under different light quality conditions. The wild type data are the same as that plotted in Fig. 1B. Error bars represent SEM of three biological replicates. Statistical significance (p-value) is denoted by *, <0.05; **, 0.01; ***, 0.001. For a full list of subunits used for this analysis, see Supplementary Table S3.

Supplementary Figure 4

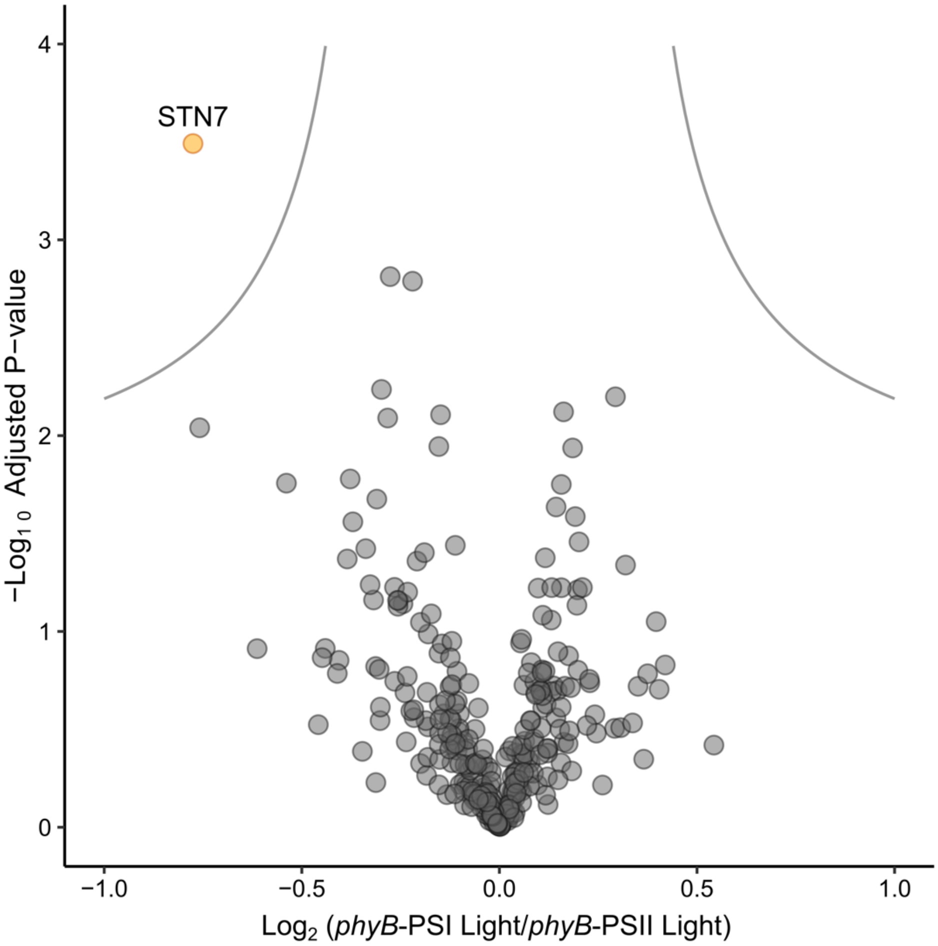

Supplementary Figure 4. Differentially accumulating thylakoid protein in *phyB*

A volcano plot depicts differentially expressed thylakoid proteins in a comparison of *phyB* PSI vs PSII light proteome samples. The plot properties and data statistics are the same as in Fig. 3A. Each data point is the mean riBAQ value of three biological replicates.

Supplementary Figure 5

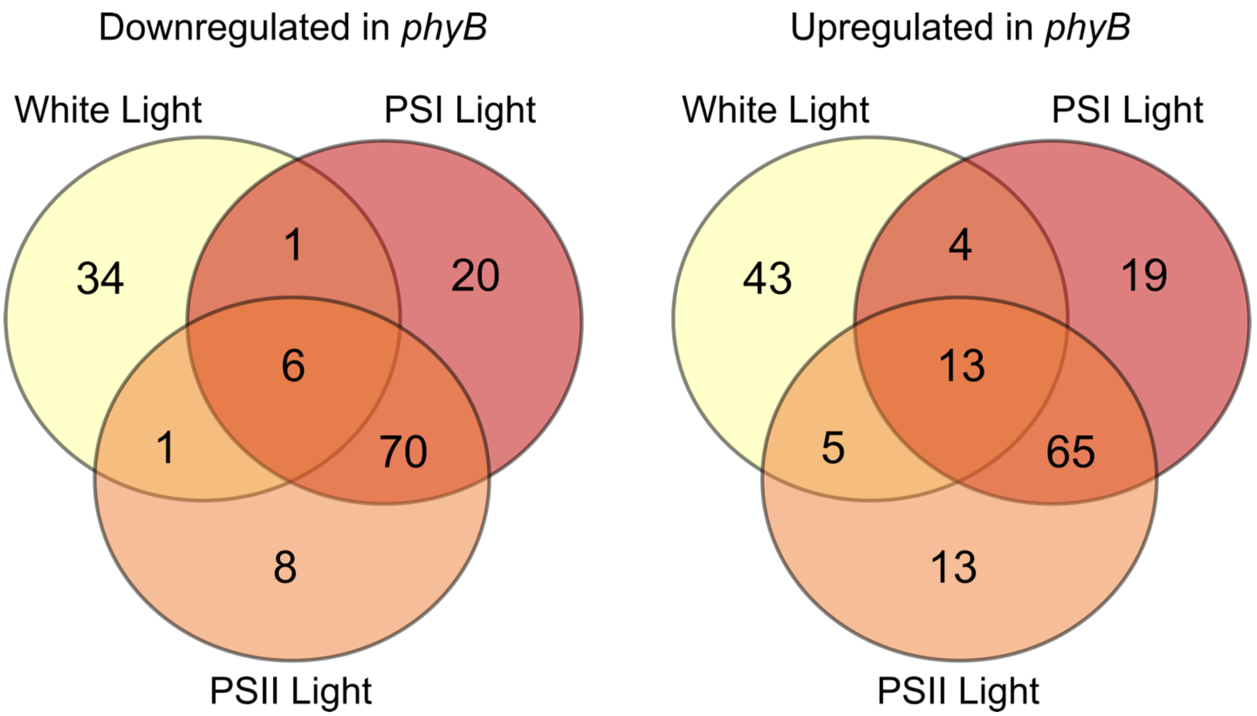

Supplementary Figure 5. Up or down-regulated thylakoid proteins in *phyB* vs wild type proteome comparison

A Venn diagram showing the number of unique and shared up or down-regulated thylakoid proteins in a proteome comparison of *phyB* vs wild type under PSI, PSII, and white light conditions. The Venn diagram was generated using the University of Ghent Bioinformatics server (https://bioinformatics.psb.ugent.be/webtools/Venn/). The differentially accumulating proteins are listed in Supplementary Tables S6 and S7.

Supplementary Table 1 List of all detected proteins in LC-MS/MS analysis

Accession	Symbol	Name	Subacon Consensus Localization
AT1G01080.1	RNA-binding (RRM/RBD/RNP motifs) family protein	RNA-binding (RRM/RBD/RNP motifs) family protein	plastid
AT1G01100.4	60S acidic ribosomal protein family	60S acidic ribosomal protein family	cytosol
AT1G01170.2	Protein of unknown function (DUF1138)	Protein of unknown function (DUF1138)	plasma membrane
AT1G01320.2	Tetratricopeptide repeat (TPR)-like superfamily protein	Tetratricopeptide repeat (TPR)-like superfamily protein	nucleus
AT1G01470.1	LEA14, LSR3	Late embryogenesis abundant protein	cytosol
AT1G01610.1	ATGPAT4, GPAT4	glycerol-3-phosphate acyltransferase 4	golgi
AT1G01790.1	KEA1, ATKEA1	K+ efflux antiporter 1	plastid
AT1G02140.1	MEE63, MAGO, HAP1	mago nashi family protein	nucleus
AT1G02560.1	CLPP5, NCLPP5, NCLPP1	nuclear encoded CLP protease 5	plastid
AT1G02780.1	emb2386	Ribosomal protein L19e family protein	cytosol
AT1G02910.1	LPA1	tetratricopeptide repeat (TPR)-containing protein	plastid
AT1G03160.1	FZL	FZO-like	plastid
AT1G03475.1	LIN2, HEMF1, ATCPO-I	Coproporphyrinogen III oxidase	plastid
AT1G03600.1	PSB27	photosystem II family protein	plastid
AT1G03630.2	POR C, PORC	protochlorophyllide oxidoreductase C	plastid
AT1G03680.1	ATHM1, TRX-M1, ATM1, THM1	thioredoxin M-type 1	plastid
AT1G03860.1	ATPHB2, PHB2	prohibitin 2	mitochondrion
AT1G04170.1	EIF2 GAMMA	eukaryotic translation initiation factor 2 gamma subunit	cytosol
AT1G04270.2	RPS15	cytosolic ribosomal protein S15	cytosol
AT1G04410.1	Lactate/malate dehydrogenase family protein	Lactate/malate dehydrogenase family protein	vacuole
AT1G04430.2	S-adenosyl-L-methionine-dependent methyltransferases superfamily protein	S-adenosyl-L-methionine-dependent methyltransferases superfamily protein	endoplasmic reticulum
AT3G04400.2	emb2171	Ribosomal protein L14p/L23e family protein	cytosol
AT1G04530.1	TPR4	Tetratricopeptide repeat (TPR)-like superfamily protein	cytosol
AT1G04620.1	coenzyme F420 hydrogenase family / dehydrogenase, beta subunit family	coenzyme F420 hydrogenase family / dehydrogenase, beta subunit family	plastid
AT1G50010.1	TUA2	tubulin alpha-2 chain	cytosol
AT1G05190.1	emb2394	Ribosomal protein L6 family	plastid
AT1G06190.1	Rho termination factor	Rho termination factor	plastid

AT1G06220.1	MEE5, CLO, GFA1	Ribosomal protein S5/Elongation factor G/III/V family protein	nucleus
AT1G06400.1	ARA2, ATRABA1A, ATRAB11E, ARA-2	Ras-related small GTP-binding family protein	cytosol
AT1G06410.1	ATTPS7, TPS7, ATTPSA	trehalose-phosphatase/synthase 7	cytosol
AT1G06430.1	FTSH8	FTSH protease 8	plastid
AT1G06530.1	Tropomyosin-related	Tropomyosin-related	mitochondrion
AT1G06680.2	PSBP-1, OEE2, PSII-P	photosystem II subunit P-1	plastid
AT1G06690.1	NAD(P)-linked oxidoreductase superfamily protein	NAD(P)-linked oxidoreductase superfamily protein	plastid
AT1G06950.1	ATTIC110, TIC110	translocon at the inner envelope membrane of chloroplasts 110	plastid
AT1G07110.1	F2KP, ATF2KP, FKFBP	fructose-2,6-bisphosphatase	cytosol
AT1G07320.1	RPL4	ribosomal protein L4	plastid
AT5G59970.1	Histone superfamily protein	Histone superfamily protein	nucleus
AT5G59850.1	Ribosomal protein S8 family protein	Ribosomal protein S8 family protein	cytosol
AT1G07810.1	ECA1, ATECA1, ACA3	ER-type Ca2+-ATPase 1	endoplasmic reticulum
AT1G07890.8	APX1, MEE6, CS1, ATAPX1, ATAPX01	ascorbate peroxidase 1	cytosol
AT5G60390.3	GTP binding Elongation factor Tu family protein	GTP binding Elongation factor Tu family protein	cytosol
AT1G08360.1	Ribosomal protein L1p/L10e family	Ribosomal protein L1p/L10e family	cytosol
AT1G08470.1	SSL3	strictosidine synthase-like 3	endoplasmic reticulum
AT1G08480.1	unknown protein;FUNCTIONS IN: molecular_function unknown;INVOLVED IN: biological_process unknown;LOCATED IN: mitochondrion, plasma membrane, plastid, vacuole;EXPRESSED IN: 27 plant structures;EXPRESSED DURING: 15 growth stage	unknown protein;FUNCTIONS IN: molecular_function unknown;INVOLVED IN: biological_process unknown;LOCATED IN: mitochondrion, plasma membrane, plastid, vacuole;EXPRESSED IN: 27 plant structures;EXPRESSED DURING: 15 growth stage	mitochondrion
AT1G08520.1	ALB1, ALB-1V, V157, PDE166, CHLD	ALBINA 1	plastid
AT1G08550.2	NPQ1, AVDE1	non-photochemical quenching 1	plastid
AT1G08640.1	CJD1	Chloroplast J-like domain 1	plastid
AT1G54690.1	HTA3, H2AXB, G-H2AX, GAMMA-H2AX	gamma histone variant H2AX	nucleus
AT1G09020.1	SNF4, ATSNF4	homolog of yeast sucrose nonfermenting 4	golgi
AT5G65360.1	Histone superfamily protein	Histone superfamily protein	nucleus
AT1G09210.1	CRT1b, AtCRT1b	calreticulin 1b	endoplasmic reticulum
AT1G09310.1	Protein of unknown function, DUF538	Protein of unknown function, DUF538	cytosol
AT1G09340.1	CRB, CSP41B, HIP1.3	chloroplast RNA binding	plastid

AT1G57860.1	Translation protein SH3-like family protein	Translation protein SH3-like family protein	cytosol
AT1G09630.1	ATRAB11C, ATRABA2A, ATRAB-A2A, RAB-A2A, RAB11c	RAB GTPase 11C	plasma membrane,cytosol
AT1G09640.1	Translation elongation factor EF1B, gamma chain	Translation elongation factor EF1B, gamma chain	cytosol
AT1G09770.1	ATCDC5, CDC5, ATMYBCDC5	cell division cycle 5	nucleus
AT1G10290.1	ADL6, DRP2A	dynamin-like protein 6	golgi
AT1G10510.1	emb2004	RNI-like superfamily protein	plastid
AT3G62290.3	ARFA1E	ADP-ribosylation factor A1E	golgi
AT1G10840.1	TIF3H1	translation initiation factor 3 subunit H1	cytosol
AT1G10950.1	TMN1, AtTMN1	transmembrane nine 1	golgi
AT1G11750.1	CLPP6, NCLPP1, NCLPP6	CLP protease proteolytic subunit 6	plastid
AT1G11860.3	Glycine cleavage T-protein family	Glycine cleavage T-protein family	mitochondrion
AT1G11890.1	SEC22, ATSEC22	Synaptobrevin family protein	endoplasmic reticulum
AT1G11910.1	APA1, ATAPA1	aspartic proteinase A1	vacuole
AT1G12250.2	Pentapeptide repeat-containing protein	Pentapeptide repeat-containing protein	plastid
AT1G12840.1	DET3, ATVHA-C	vacuolar ATP synthase subunit C (VATC) / V-ATPase C subunit / vacuolar proton pump C subunit (DET3)	vacuole,golgi
AT1G12900.4	GAPA-2	glyceraldehyde 3-phosphate dehydrogenase A subunit 2	plastid
AT1G12920.1	ERF1-2	eukaryotic release factor 1-2	cytosol
AT1G13110.1	CYP71B7	cytochrome P450, family 71 subfamily B, polypeptide 7	plasma membrane
AT1G14150.1	PQL1, PQL2	PsbQ-like 2	plastid
AT1G14320.1	SAC52, RPL10, RPL10A	Ribosomal protein L16p/L10e family protein	cytosol
AT1G14345.1	NAD(P)-linked oxidoreductase superfamily protein	NAD(P)-linked oxidoreductase superfamily protein	plastid
AT1G14650.1	SWAP (Suppressor-of-White-APricot)/surp domain-containing protein / ubiquitin family protein	SWAP (Suppressor-of-White-APricot)/surp domain-containing protein / ubiquitin family protein	nucleus
AT1G15120.1	Ubiquinol-cytochrome C reductase hinge protein	Ubiquinol-cytochrome C reductase hinge protein	mitochondrion
AT1G15690.1	AVP1, ATAVP3, AVP-3, AtVHP1;1	Inorganic H pyrophosphatase family protein	vacuole
AT1G15820.1	LHCB6, CP24	light harvesting complex photosystem II subunit 6	plastid
AT1G15930.2	Ribosomal protein L7Ae/L30e/S12e/Gadd45 family protein	Ribosomal protein L7Ae/L30e/S12e/Gadd45 family protein	cytosol
AT1G15980.1	NDF1, NDH48	NDH-dependent cyclic electron flow 1	plastid

AT1G16445.1	S-adenosyl-L-methionine-dependent methyltransferases superfamily protein	S-adenosyl-L-methionine-dependent methyltransferases superfamily protein	plastid
AT1G79210.3	N-terminal nucleophile aminohydrolases (Ntn hydrolases) superfamily protein	N-terminal nucleophile aminohydrolases (Ntn hydrolases) superfamily protein	cytosol
AT1G16610.2	SR45, RNPS1	arginine/serine-rich 45	plastid
AT1G16720.1	HCF173	high chlorophyll fluorescence phenotype 173	plastid
AT1G16880.1	uridylyltransferase-related	uridylyltransferase-related	plastid
AT1G16920.1	RAB11, ATRABA1B, RABA1b	RAB GTPase homolog A1B	plasma membrane,golgi
AT1G17100.1	SOUL heme-binding family protein	SOUL heme-binding family protein	extracellular
AT1G17220.1	FUG1	Translation initiation factor 2, small GTP-binding protein	plastid
AT1G17290.1	AlaAT1	alanine aminotransferas	mitochondrion
AT1G17470.2	ATDRG1, ATDRG, DRG1	developmentally regulated G-protein 1	cytosol
AT1G17880.1	BTF3, ATBTF3	basic transcription factor 3	nucleus
AT1G18080.1	ATARCA, RACK1A_AT, RACK1A	Transducin/WD40 repeat-like superfamily protein	cytosol
AT1G18170.1	FKBP-like peptidyl-prolyl cis-trans isomerase family protein	FKBP-like peptidyl-prolyl cis-trans isomerase family protein	plastid
AT1G18450.1	ATARP4, ARP4	actin-related protein 4	nucleus
AT1G18540.1	Ribosomal protein L6 family protein	Ribosomal protein L6 family protein	cytosol
AT1G19360.1	Nucleotide-diphospho-sugar transferase family protein	Nucleotide-diphospho-sugar transferase family protein	golgi
AT1G19570.1	DHAR1, ATDHAR1, DHAR5	dehydroascorbate reductase	peroxisome
AT1G19580.1	GAMMA CA1	gamma carbonic anhydrase 1	mitochondrion
AT1G19670.1	ATCLH1, CORI1, ATHCOR1, CLH1	chlorophyllase 1	cytosol
AT1G19870.1	iqd32	IQ-domain 32	cytosol
AT1G20010.1	TUB5	tubulin beta-5 chain	cytosol
AT1G20020.1	ATLFNR2, FNR2	ferredoxin-NADP(+)-oxidoreductase 2	plastid
AT1G20340.1	DRT112, PETE2	Cupredoxin superfamily protein	plastid
AT1G20440.1	COR47, RD17, AtCOR47	cold-regulated 47	nucleus
AT1G20450.2	LTI29, LTI45, ERD10	Dehydrin family protein	cytosol
AT1G20620.1	CAT3, SEN2, ATCAT3	catalase 3	peroxisome
AT1G20693.2	HMGB2, HMG BETA 1, NFD2, NFD02	high mobility group B2	nucleus
AT1G20960.1	emb1507	U5 small nuclear ribonucleoprotein helicase, putative	nucleus
AT1G21500.1	unknown protein;Has 29 Blast hits to 29 proteins in 12 species: Archae - 0;Bacteria -	unknown protein;Has 29 Blast hits to 29 proteins in 12 species: Archae - 0;Bacteria - 0;Metazoa -	plastid

	0;Metazoa - 0;Fungi - 2;Plants - 27;Viruses - 0;Other Eukaryotes - 0 (source: NCBI BLink).	0;Fungi - 2;Plants - 27;Viruses - 0;Other Eukaryotes - 0 (source: NCBI BLink).	
AT1G21750.1	ATPDIL1-1, ATPDI5, PDI5, PDIL1-1	PDI-like 1-1	endoplasmic reticulum
AT1G22300.1	GRF10, 14-3-3EPSILON, GF14 EPSILON	general regulatory factor 10	cytosol
AT1G22450.1	COX6B, ATCOX6B2	cytochrome C oxidase 6B	mitochondrion
AT1G22520.1	Domain of unknown function (DUF543)	Domain of unknown function (DUF543)	mitochondrion
AT1G22700.3	Tetratricopeptide repeat (TPR)-like superfamily protein	Tetratricopeptide repeat (TPR)-like superfamily protein	plastid
AT1G22740.1	RAB7, ATRABG3B, RAB75, RABG3B	RAB GTPase homolog G3B	vacuole
AT4G09800.1	RPS18C	S18 ribosomal protein	cytosol
AT1G22840.1	CYTC-1, ATCYTC-A	CYTOCHROME C-1	mitochondrion
AT1G70600.1	Ribosomal protein L18e/L15 superfamily protein	Ribosomal protein L18e/L15 superfamily protein	cytosol
AT1G23310.1	GGT1, AOAT1, GGAT1	glutamate:glyoxylate aminotransferase	peroxisome
AT1G24180.1	IAR4	Thiamin diphosphate-binding fold (THDP-binding) superfamily protein	mitochondrion
AT1G24490.2	ALB4, ARTEMIS	OxaA/YidC-like membrane insertion protein	plastid
AT1G25490.1	RCN1, REGA, ATB BETA BETA, EER1	ARM repeat superfamily protein	plasma membrane
AT1G26630.1	FBR12, ATELF5A-2, ELF5A-2	Eukaryotic translation initiation factor 5A-1 (eIF-5A 1) protein	cytosol
AT1G27090.1	glycine-rich protein	glycine-rich protein	nucleus
AT1G27390.1	TOM20-2	translocase outer membrane 20-2	mitochondrion
AT1G27400.1	Ribosomal protein L22p/L17e family protein	Ribosomal protein L22p/L17e family protein	plastid,nucleus,cytosol
AT1G27970.1	NTF2B	nuclear transport factor 2B	nucleus
AT1G28140.1	unknown protein;FUNCTIONS IN: molecular_function unknown;INVOLVED IN: biological_process unknown;LOCATED IN: chloroplast;EXPRESSED IN: 22 plant structures;EXPRESSED DURING: 13 growth stages;CONTAINS InterPro DOMAIN/s: Protei	unknown protein;FUNCTIONS IN: molecular_function unknown;INVOLVED IN: biological_process unknown;LOCATED IN: chloroplast;EXPRESSED IN: 22 plant structures;EXPRESSED DURING: 13 growth stages;CONTAINS InterPro DOMAIN/s: Protei	plastid
AT1G28200.1	FIP1	FH interacting protein 1	golgi
AT1G29250.1	Alba DNA/RNA-binding protein	Alba DNA/RNA-binding protein	cytosol
AT2G34250.2	SecY protein transport family protein	SecY protein transport family protein	endoplasmic reticulum

AT1G29700.1	Metallo-hydrolase/oxidoreductase superfamily protein	Metallo-hydrolase/oxidoreductase superfamily protein	plastid
AT1G29930.1	CAB1, AB140, CAB140, LHCB1.3	chlorophyll A/B binding protein 1	plastid
AT1G30230.1	Glutathione S-transferase, C-terminal-like;Translation elongation factor EF1B/ribosomal protein S6	Glutathione S-transferase, C-terminal-like;Translation elongation factor EF1B/ribosomal protein S6	cytosol
AT1G30360.1	ERD4	Early-responsive to dehydration stress protein (ERD4)	plasma membrane
AT1G30380.1	PSAK	photosystem I subunit K	plastid
AT1G30470.3	SIT4 phosphatase-associated family protein	SIT4 phosphatase-associated family protein	nucleus
AT1G30580.1	GTP binding	GTP binding	cytosol
AT1G30630.1	Coatomer epsilon subunit	Coatomer epsilon subunit	golgi
AT1G30690.2	Sec14p-like phosphatidylinositol transfer family protein	Sec14p-like phosphatidylinositol transfer family protein	cytosol
AT1G31330.1	PSAF	photosystem I subunit F	plastid
AT1G32060.1	PRK	phosphoribulokinase	plastid
AT1G32220.1	NAD(P)-binding Rossmann-fold superfamily protein	NAD(P)-binding Rossmann-fold superfamily protein	plastid
AT1G32470.1	Single hybrid motif superfamily protein	Single hybrid motif superfamily protein	mitochondrion
AT1G32990.1	PRPL11	plastid ribosomal protein l11	plastid
AT1G33040.1	NACA5	nascent polypeptide-associated complex subunit alpha-like protein 5	nucleus
AT1G33140.1	PGY2	Ribosomal protein L6 family	cytosol
AT1G34000.1	OHP2	one-helix protein 2	plastid
AT1G34430.1	EMB3003	2-oxoacid dehydrogenases acyltransferase family protein	plastid
AT1G35160.1	GRF4, 14-3-3PHI, GF14 PHI	GF14 protein phi chain	cytosol
AT1G35620.1	ATPDIL5-2, ATPDI8, PDI8, PDIL5-2	PDI-like 5-2	endoplasmic reticulum
AT1G35680.1	Ribosomal protein L21	Ribosomal protein L21	plastid
AT1G35720.1	ANNAT1, OXY5, ATOXY5	annexin 1	peroxisome
AT1G36730.1	Translation initiation factor IF2/IF5	Translation initiation factor IF2/IF5	cytosol
AT1G37130.1	NIA2, B29, NIA2-1, CHL3, NR, NR2, ATNR2	nitrate reductase 2	cytosol
AT1G42550.1	PMI1	plastid movement impaired1	cytosol
AT1G42960.1	expressed protein localized to the inner membrane of the chloroplast.	expressed protein localized to the inner membrane of the chloroplast.	plastid
AT1G42970.1	GAPB	glyceraldehyde-3-phosphate dehydrogenase B subunit	plastid
AT1G43170.9	RP1	ribosomal protein 1	cytosol

AT1G43890.3	ATRAB18, ATRABC1, RAB18-1, RABC1, ATRAB-C1, RAB18	RAB GTPASE HOMOLOG B18	cytosol
AT1G44575.1	NPQ4, PSBS	Chlorophyll A-B binding family protein	plastid
AT1G45201.2	ATTLL1, TLL1	triacylglycerol lipase-like 1	endoplasmic reticulum
AT1G45474.2	LHCA5	photosystem I light harvesting complex gene 5	plastid
AT1G47128.1	RD21, RD21A	Granulin repeat cysteine protease family protein	extracellular
AT1G47260.1	APFI, GAMMA CA2	gamma carbonic anhydrase 2	mitochondrion
AT1G47420.1	SDH5	succinate dehydrogenase 5	mitochondrion
AT1G48030.2	mtLPD1	mitochondrial lipoamide dehydrogenase 1	mitochondrion
AT1G48410.1	AGO1	Stabilizer of iron transporter SufD / Polynucleotidyl transferase	nucleus
AT1G48600.1	PMEAMT, AtPMEAMT	S-adenosyl-L-methionine-dependent methyltransferases superfamily protein	cytosol
AT1G48610.1	AT hook motif-containing protein	AT hook motif-containing protein	nucleus
AT1G48620.1	HON5	high mobility group A5	nucleus
AT1G48830.2	Ribosomal protein S7e family protein	Ribosomal protein S7e family protein	cytosol
AT1G48920.1	ATNUC-L1, PARL1, NUC-L1	nucleolin like 1	nucleus
AT1G49750.1	Leucine-rich repeat (LRR) family protein	Leucine-rich repeat (LRR) family protein	extracellular
AT1G49760.2	PAB8	poly(A) binding protein 8	nucleus
AT1G49970.1	CLPR1, NCLPP5, SVR2	CLP protease proteolytic subunit 1	plastid
AT1G50250.1	FTSH1	FTSH protease 1	plastid
AT1G50450.1	Saccharopine dehydrogenase	Saccharopine dehydrogenase	plastid
AT1G51100.1	unknown protein;FUNCTIONS IN: molecular_function unknown;INVOLVED IN: biological_process unknown;LOCATED IN: chloroplast, chloroplast stroma;EXPRESSED IN: 22 plant structures;EXPRESSED DURING: 13 growth stages;Has 26 Blast h	unknown protein;FUNCTIONS IN: molecular_function unknown;INVOLVED IN: biological_process unknown;LOCATED IN: chloroplast, chloroplast stroma;EXPRESSED IN: 22 plant structures;EXPRESSED DURING: 13 growth stages;Has 26 Blast h	plastid
AT1G51110.1	Plastid-lipid associated protein PAP / fibrillin family protein	Plastid-lipid associated protein PAP / fibrillin family protein	plastid
AT1G51400.1	Photosystem II 5 kD protein	Photosystem II 5 kD protein	plastid
AT1G51510.1	Y14	RNA-binding (RRM/RBD/RNP motifs) family protein	nucleus,cytosol
AT1G51980.1	Insulinase (Peptidase family M16) protein	Insulinase (Peptidase family M16) protein	mitochondrion
AT1G52300.1	Zinc-binding ribosomal protein family protein	Zinc-binding ribosomal protein family protein	cytosol

AT1G52360.1	Coatomer, beta subunit	Coatomer, beta subunit	cytosol
AT1G52400.3	BGLU18	beta glucosidase 18	endoplasmic reticulum
AT1G52510.1	alpha/beta-Hydrolases superfamily protein	alpha/beta-Hydrolases superfamily protein	plastid
AT1G52740.1	HTA9	histone H2A protein 9	nucleus
AT1G53210.1	sodium/calcium exchanger family protein / calcium-binding EF hand family protein	sodium/calcium exchanger family protein / calcium-binding EF hand family protein	plasma membrane
AT1G53240.1	mMDH1	Lactate/malate dehydrogenase family protein	mitochondrion
AT1G53750.1	RPT1A	regulatory particle triple-A 1A	nucleus,cytosol
AT1G54220.2	Dihydrolipoamide acetyltransferase, long form protein	Dihydrolipoamide acetyltransferase, long form protein	mitochondrion
AT1G54270.1	EIF4A-2	eif4a-2	cytosol
AT1G54350.1	ABC transporter family protein	ABC transporter family protein	plastid
AT1G54410.1	dehydrin family protein	dehydrin family protein	cytosol
AT1G54500.1	Rubredoxin-like superfamily protein	Rubredoxin-like superfamily protein	plastid
AT1G54520.1	unknown protein;FUNCTIONS IN: molecular_function unknown;INVOLVED IN: biological_process unknown;LOCATED IN: chloroplast;EXPRESSED IN: 24 plant structures;EXPRESSED DURING: 15 growth stages;CONTAINS InterPro DOMAIN/s: Protei	unknown protein;FUNCTIONS IN: molecular_function unknown;INVOLVED IN: biological_process unknown;LOCATED IN: chloroplast;EXPRESSED IN: 24 plant structures;EXPRESSED DURING: 15 growth stages;CONTAINS InterPro DOMAIN/s: Protei	plastid
AT1G54780.1	TLP18.3	thylakoid lumen 18.3 kDa protein	plastid
AT1G55160.2	unknown protein;FUNCTIONS IN: molecular_function unknown;INVOLVED IN: biological_process unknown;LOCATED IN: mitochondrion, plastid;EXPRESSED IN: 22 plant structures;EXPRESSED DURING: 13 growth stages;BEST Arabidopsis thalia	unknown protein;FUNCTIONS IN: molecular_function unknown;INVOLVED IN: biological_process unknown;LOCATED IN: mitochondrion, plastid;EXPRESSED IN: 22 plant structures;EXPRESSED DURING: 13 growth stages;BEST Arabidopsis thalia	mitochondrion
AT1G55450.1	S-adenosyl-L-methionine-dependent methyltransferases superfamily protein	S-adenosyl-L-methionine-dependent methyltransferases superfamily protein	golgi
AT1G55480.1	ZKT	protein containing PDZ domain, a K-box domain, and a TPR region	plastid
AT1G55490.2	CPN60B, LEN1	chaperonin 60 beta	plastid
AT1G55670.1	PSAG	photosystem I subunit G	plastid
AT1G56070.1	LOS1	Ribosomal protein S5/Elongation factor G/III/V family protein	cytosol

AT1G56330.1	SAR1, ATSAR1, ATSARA1B, ATSAR1B, SAR1B	secretion-associated RAS 1B	endoplasmic reticulum,cytosol
AT1G56340.1	CRT1, CRT1a, AtCRT1a	calreticulin 1a	endoplasmic reticulum
AT1G56500.1	haloacid dehalogenase-like hydrolase family protein	haloacid dehalogenase-like hydrolase family protein	plastid
AT1G57720.2	Translation elongation factor EF1B, gamma chain	Translation elongation factor EF1B, gamma chain	cytosol
AT1G59610.1	ADL3, CF1, DRP2B, DL3	dynamin-like 3	plasma membrane
AT1G59870.1	PEN3, PDR8, ATPDR8, ABCG36, ATABCG36	ABC-2 and Plant PDR ABC-type transporter family protein	plasma membrane
AT1G59900.1	AT-E1 ALPHA, E1 ALPHA	pyruvate dehydrogenase complex E1 alpha subunit	mitochondrion
AT1G61250.2	SC3	secretory carrier 3	plasma membrane
AT1G61520.2	LHCA3	photosystem I light harvesting complex gene 3	Plastid
AT1G62020.1	Coatomer, alpha subunit	Coatomer, alpha subunit	cytosol
AT1G62390.1	Phox2	Octicosapeptide/Phox/Bem1p (PB1) domain-containing protein / tetratricopeptide repeat (TPR)-containing protein	nucleus
AT1G62750.1	ATSCO1, ATSCO1/CPEF-G, SCO1	Translation elongation factor EFG/EF2 protein	plastid
AT1G63210.1	Transcription elongation factor Spt6	Transcription elongation factor Spt6	nucleus
AT1G63940.4	MDAR6	monodehydroascorbate reductase 6	plastid
AT1G64355.1	unknown protein;FUNCTIONS IN: molecular_function unknown;INVOLVED IN: biological_process unknown;LOCATED IN: chloroplast;EXPRESSED IN: 23 plant structures;EXPRESSED DURING: 13 growth stages;CONTAINS InterPro DOMAIN/s: Protei	unknown protein;FUNCTIONS IN: molecular_function unknown;INVOLVED IN: biological_process unknown;LOCATED IN: chloroplast;EXPRESSED IN: 23 plant structures;EXPRESSED DURING: 13 growth stages;CONTAINS InterPro DOMAIN/s: Protei	plastid
AT1G64740.1	TUA1	alpha-1 tubulin	cytosol
AT1G64770.1	NDF2, NDH45	NDH-dependent cyclic electron flow 1	plastid
AT1G64970.1	G-TMT, TMT1, VTE4	gamma-tocopherol methyltransferase	plastid
AT1G65230.1	Uncharacterized conserved protein (DUF2358)	Uncharacterized conserved protein (DUF2358)	plastid
AT1G65260.1	PTAC4, VIPP1	plastid transcriptionally active 4	plastid
AT1G65270.3	unknown protein;FUNCTIONS IN: molecular_function unknown;INVOLVED IN: biological_process unknown;LOCATED IN: endoplasmic reticulum, plasma membrane;EXPRESSED	unknown protein;FUNCTIONS IN: molecular_function unknown;INVOLVED IN: biological_process unknown;LOCATED IN: endoplasmic reticulum, plasma membrane;EXPRESSED IN: 24	extracellular

	IN: 24 plant structures;EXPRESSED DURING: 13 growth stages;Has 353	plant structures;EXPRESSED DURING: 13 growth stages;Has 353	
AT1G65540.1	LETM1-like protein	LETM1-like protein	mitochondrion
AT1G65820.1	microsomal glutathione s-transferase, putative	microsomal glutathione s-transferase, putative	endoplasmic reticulum
AT1G65930.1	cICDH	cytosolic NADP+-dependent isocitrate dehydrogenase	cytosol
AT1G65960.2	GAD2	glutamate decarboxylase 2	cytosol
AT1G66580.1	SAG24, RPL10C	senescence associated gene 24	cytosol
AT1G66970.1	SVL2	SHV3-like 2	plasma membrane
AT1G67090.1	RBCS1A	ribulose bisphosphate carboxylase small chain 1A	plastid
AT1G67230.1	LINC1	little nuclei1	nucleus
AT1G67350.2	unknown protein;FUNCTIONS IN: molecular_function unknown;INVOLVED IN: photorespiration;LOCATED IN: mitochondrial membrane, mitochondrial respiratory chain complex I, respiratory chain complex I;EXPRESSED IN: 24 plant structure	unknown protein;FUNCTIONS IN: molecular_function unknown;INVOLVED IN: photorespiration;LOCATED IN: mitochondrial membrane, mitochondrial respiratory chain complex I, respiratory chain complex I;EXPRESSED IN: 24 plant structure	cytosol
AT1G67680.1	SRP72 RNA-binding domain	SRP72 RNA-binding domain	nucleus
AT1G67700.2	unknown protein;FUNCTIONS IN: molecular_function unknown;INVOLVED IN: biological_process unknown;LOCATED IN: chloroplast, chloroplast envelope;EXPRESSED IN: 22 plant structures;EXPRESSED DURING: 13 growth stages;Has 49 Blast	unknown protein;FUNCTIONS IN: molecular_function unknown;INVOLVED IN: biological_process unknown;LOCATED IN: chloroplast, chloroplast envelope;EXPRESSED IN: 22 plant structures;EXPRESSED DURING: 13 growth stages;Has 49 Blast	plastid
AT1G67730.1	YBR159, KCR1, ATKCR1	beta-ketoacyl reductase 1	endoplasmic reticulum
AT1G68010.1	HPR, ATHPR1	hydroxypyruvate reductase	peroxisome
AT1G68590.2	Ribosomal protein PSRP-3/Ycf65	Ribosomal protein PSRP-3/Ycf65	plastid
AT1G68680.1	unknown protein;FUNCTIONS IN: molecular_function unknown;INVOLVED IN: biological_process unknown;LOCATED IN: chloroplast;EXPRESSED IN: 23 plant structures;EXPRESSED DURING: 16 growth stages;Has 20 Blast hits to 20 proteins i	unknown protein;FUNCTIONS IN: molecular_function unknown;INVOLVED IN: biological_process unknown;LOCATED IN: chloroplast;EXPRESSED IN: 23 plant structures;EXPRESSED DURING: 16 growth stages;Has 20 Blast hits to 20 proteins i	mitochondrion
AT1G68830.1	STN7	STT7 homolog STN7	plastid

AT1G69200.1	FLN2	fructokinase-like 2	plastid
AT1G69260.1	AFP1	ABI five binding protein	nucleus
AT1G69620.1	RPL34	ribosomal protein L34	cytosol
AT1G69840.7	SPFH/Band 7/PHB domain-containing membrane-associated protein family	SPFH/Band 7/PHB domain-containing membrane-associated protein family	cytosol
AT1G70070.1	EMB25, PDE317, ISE2	DEAD/DEAH box helicase, putative	plastid
AT1G70320.1	UPL2	ubiquitin-protein ligase 2	cytosol
AT1G70410.2	ATBCA4, BCA4	beta carbonic anhydrase 4	plasma membrane
AT1G70760.1	CRR23	inorganic carbon transport protein-related	plastid
AT1G70770.2	Protein of unknown function DUF2359, transmembrane	Protein of unknown function DUF2359, transmembrane	golgi,cytosol
AT1G71220.1	EBS1, UGGT, PSL2	UDP-glucose:glycoprotein glucosyltransferases;transferases, transferring hexosyl groups;transferases, transferring glycosyl groups	endoplasmic reticulum
AT1G71480.1	Nuclear transport factor 2 (NTF2) family protein	Nuclear transport factor 2 (NTF2) family protein	plastid
AT1G71500.1	Rieske (2Fe-2S) domain-containing protein	Rieske (2Fe-2S) domain-containing protein	plastid
AT1G71695.1	Peroxidase superfamily protein	Peroxidase superfamily protein	extracellular
AT1G71810.1	Protein kinase superfamily protein	Protein kinase superfamily protein	plastid
AT1G72150.1	PATL1	PATELLIN 1	plasma membrane
AT1G72160.1	Sec14p-like phosphatidylinositol transfer family protein	Sec14p-like phosphatidylinositol transfer family protein	cytosol
AT1G72170.1	Domain of unknown function (DUF543)	Domain of unknown function (DUF543)	mitochondrion
AT1G72370.2	P40, AP40, RP40, RPSAA	40s ribosomal protein SA	cytosol
AT1G72610.1	GLP1, ATGER1, GER1	germin-like protein 1	extracellular
AT1G72640.2	NAD(P)-binding Rossmann-fold superfamily protein	NAD(P)-binding Rossmann-fold superfamily protein	plastid
AT1G73060.1	LPA3	Low PSII Accumulation 3	plastid
AT1G73110.1	P-loop containing nucleoside triphosphate hydrolases superfamily protein	P-loop containing nucleoside triphosphate hydrolases superfamily protein	plastid
AT1G73230.1	Nascent polypeptide-associated complex NAC	Nascent polypeptide-associated complex NAC	nucleus
AT1G73990.1	SPPA, SPPA1	signal peptide peptidase	plastid
AT1G74050.1	Ribosomal protein L6 family protein	Ribosomal protein L6 family protein	cytosol
AT1G74070.1	Cyclophilin-like peptidyl-prolyl cis-trans isomerase family protein	Cyclophilin-like peptidyl-prolyl cis-trans isomerase family protein	plastid

AT3G55750.1	Ribosomal protein L35Ae family protein	Ribosomal protein L35Ae family protein	cytosol
AT1G74470.1	Pyridine nucleotide-disulphide oxidoreductase family protein	Pyridine nucleotide-disulphide oxidoreductase family protein	plastid
AT1G74560.1	NRP1	NAP1-related protein 1	nucleus,cytosol
AT1G74730.1	Protein of unknown function (DUF1118)	Protein of unknown function (DUF1118)	plastid
AT1G74970.1	RPS9, TWN3	ribosomal protein S9	plastid
AT1G75350.1	emb2184	Ribosomal protein L31	plastid
AT1G75690.1	DnaJ/Hsp40 cysteine-rich domain superfamily protein	DnaJ/Hsp40 cysteine-rich domain superfamily protein	plastid
AT1G75750.2	GASA1	GAST1 protein homolog 1	extracellular
AT1G75950.1	SKP1, ASK1, ATSKP1, SKP1A, UIP1	S phase kinase-associated protein 1	cytosol
AT1G76030.1	ATPase, V1 complex, subunit B protein	ATPase, V1 complex, subunit B protein	vacuole,golgi
AT1G76180.2	ERD14	Dehydrin family protein	nucleus
AT1G76400.1	Ribophorin I	Ribophorin I	endoplasmic reticulum
AT1G76450.1	Photosystem II reaction center PsbP family protein	Photosystem II reaction center PsbP family protein	plastid
AT1G76810.1	eukaryotic translation initiation factor 2 (eIF-2) family protein	eukaryotic translation initiation factor 2 (eIF-2) family protein	cytosol
AT1G76850.1	SEC5A	exocyst complex component sec5	plasma membrane
AT1G77490.1	TAPX	thylakoidal ascorbate peroxidase	plastid
AT1G77510.1	ATPDIL1-2, PDI6, ATPDI6, PDIL1-2	PDI-like 1-2	endoplasmic reticulum
AT1G77590.1	LACS9	long chain acyl-CoA synthetase 9	plastid
AT1G78140.1	S-adenosyl-L-methionine-dependent methyltransferases superfamily protein	S-adenosyl-L-methionine-dependent methyltransferases superfamily protein	plastid
AT1G78300.1	GRF2, 14-3-3OMEGA, GF14 OMEGA	general regulatory factor 2	cytosol
AT1G78900.2	VHA-A	vacuolar ATP synthase subunit A	vacuole,golgi
AT1G78915.1	Tetratricopeptide repeat (TPR)-like superfamily protein	Tetratricopeptide repeat (TPR)-like superfamily protein	plastid
AT1G78920.1	AVP2, AVPL1, AtVHP2;1, VHP2;1, VP2	vacuolar H+-pyrophosphatase 2	golgi
AT1G79010.1	Alpha-helical ferredoxin	Alpha-helical ferredoxin	mitochondrion
AT1G79040.1	PSBR	photosystem II subunit R	plastid
AT1G79340.1	AtMC4, MC4	metacaspase 4	cytosol
AT1G79560.1	EMB156, EMB36, EMB1047, FTSH12	FTSH protease 12	plastid
AT1G79600.1	Protein kinase superfamily protein	Protein kinase superfamily protein	plastid
AT1G79850.1	RPS17, CS17, PRPS17	ribosomal protein S17	plastid
AT1G79920.2	Heat shock protein 70 (Hsp 70) family protein	Heat shock protein 70 (Hsp 70) family protein	cytosol

AT1G80030.3	Molecular chaperone Hsp40/DnaJ family protein	Molecular chaperone Hsp40/DnaJ family protein	plastid
AT1G80070.1	SUS2, EMB33, EMB177, EMB14	Pre-mRNA-processing-splicing factor	nucleus
AT1G80300.1	NTT1, ATNTT1	nucleotide transporter 1	plastid
AT1G80380.3	P-loop containing nucleoside triphosphate hydrolases superfamily protein	P-loop containing nucleoside triphosphate hydrolases superfamily protein	plastid
AT1G80410.1	EMB2753	tetratricopeptide repeat (TPR)-containing protein	cytosol
AT2G01110.1	APG2, UNE3, PGA2, TATC	Sec-independent periplasmic protein translocase	plastid
AT2G01140.1	Aldolase superfamily protein	Aldolase superfamily protein	plastid
AT2G01250.1	Ribosomal protein L30/L7 family protein	Ribosomal protein L30/L7 family protein	cytosol
AT2G01470.1	STL2P, ATSEC12	SEC12P-like 2 protein	endoplasmic reticulum
AT2G01720.1	Ribophorin I	Ribophorin I	endoplasmic reticulum
AT2G01970.1	Endomembrane protein 70 protein family	Endomembrane protein 70 protein family	golgi
AT2G02050.1	NADH-ubiquinone oxidoreductase B18 subunit, putative	NADH-ubiquinone oxidoreductase B18 subunit, putative	mitochondrion
AT2G02560.2	CAND1, ATCAND1, ETA2, TIP120, HVE	cullin-associated and neddylation dissociated	cytosol
AT2G03420.1	unknown protein;Has 38 Blast hits to 38 proteins in 17 species: Archae - 0;Bacteria - 0;Metazoa - 0;Fungi - 0;Plants - 38;Viruses - 0;Other Eukaryotes - 0 (source: NCBI BLink).	unknown protein;Has 38 Blast hits to 38 proteins in 17 species: Archae - 0;Bacteria - 0;Metazoa - 0;Fungi - 0;Plants - 38;Viruses - 0;Other Eukaryotes - 0 (source: NCBI BLink).	plastid
AT2G03440.1	NRP1, ATNRP1	nodulin-related protein 1	nucleus
AT2G03510.1	SPFH/Band 7/PHB domain-containing membrane-associated protein family	SPFH/Band 7/PHB domain-containing membrane-associated protein family	endoplasmic reticulum
AT2G04030.1	CR88, EMB1956, HSP90.5, Hsp88.1, AtHsp90.5	Chaperone protein htpG family protein	plastid
AT2G04350.2	LACS8	AMP-dependent synthetase and ligase family protein	peroxisome
AT2G05070.1	LHCB2.2, LHCB2	photosystem II light harvesting complex gene 2.2	plastid
AT2G05220.2	Ribosomal S17 family protein	Ribosomal S17 family protein	cytosol
AT2G05620.1	PGR5	proton gradient regulation 5	plastid
AT2G05710.1	ACO3	aconitase 3	mitochondrion
AT2G06850.1	EXGT-A1, EXT, XTH4	xyloglucan endotransglucosylase/hydrolase 4	extracellular
AT2G07050.1	CAS1	cycloartenol synthase 1	golgi
AT2G07698.1	ATPase, F1 complex, alpha subunit protein	ATPase, F1 complex, alpha subunit protein	mitochondrion,plastid

ATMG00480.1	ORFB, ATP8	Plant mitochondrial ATPase, F0 complex, subunit 8 protein	mitochondrion
AT2G09990.1	Ribosomal protein S5 domain 2-like superfamily protein	Ribosomal protein S5 domain 2-like superfamily protein	cytosol
AT2G10940.2	Bifunctional inhibitor/lipid-transfer protein/seed storage 2S albumin superfamily protein	Bifunctional inhibitor/lipid-transfer protein/seed storage 2S albumin superfamily protein	extracellular
AT2G13360.2	AGT, AGT1, SGAT	alanine:glyoxylate aminotransferase	peroxisome
AT2G13560.1	NAD-ME1	NAD-dependent malic enzyme 1	mitochondrion
AT2G14170.2	ALDH6B2	aldehyde dehydrogenase 6B2	cytosol
AT2G14720.2	VSR4, VSR2;1, BP80-2;1, MTV4	vacuolar sorting receptor 4	vacuole,golgi
AT2G14880.1	SWIB/MDM2 domain superfamily protein	SWIB/MDM2 domain superfamily protein	plastid
AT2G15290.1	ATTIC21, TIC21, CIA5, PIC1	translocon at inner membrane of chloroplasts 21	plastid
AT2G16940.1	Splicing factor, CC1-like	Splicing factor, CC1-like	nucleus
AT2G16950.2	TRN1, ATTRN1	transportin 1	cytosol
AT5G07090.2	Ribosomal protein S4 (RPS4A) family protein	Ribosomal protein S4 (RPS4A) family protein	cytosol
AT2G18020.1	EMB2296	Ribosomal protein L2 family	cytosol
AT2G18710.1	SCY1	SECY homolog 1	plastid
AT2G18960.1	AHA1, PMA, OST2, HA1	H(+)-ATPase 1	plasma membrane
AT2G19480.3	NAP1;2	nucleosome assembly protein 1;2	nucleus
AT2G19520.1	FVE, ACG1, MSI4, NFC4, NFC04, ATMSI4	Transducin family protein / WD-40 repeat family protein	nucleus
AT2G19680.2	Mitochondrial ATP synthase subunit G protein	Mitochondrial ATP synthase subunit G protein	mitochondrion
AT2G19730.3	Ribosomal L28e protein family	Ribosomal L28e protein family	cytosol
AT5G56670.1	Ribosomal protein S30 family protein	Ribosomal protein S30 family protein	nucleus,cytosol
AT2G19860.1	ATHXK2, HXK2	hexokinase 2	mitochondrion
AT4G29040.1	RPT2a	regulatory particle AAA-ATPase 2A	nucleus,cytosol
AT2G20260.1	PSAE-2	photosystem I subunit E-2	plastid
AT2G20360.1	NAD(P)-binding Rossmann-fold superfamily protein	NAD(P)-binding Rossmann-fold superfamily protein	mitochondrion
AT2G20420.1	ATP citrate lyase (ACL) family protein	ATP citrate lyase (ACL) family protein	mitochondrion
AT2G20530.2	ATPHB6, PHB6	prohibitin 6	mitochondrion
AT2G20580.1	RPN1A, ATRPN1A	26S proteasome regulatory subunit S2 1A	nucleus,cytosol
AT2G20630.1	PIA1	PP2C induced by AVRRPM1	cytosol
AT2G20760.1	Clathrin light chain protein	Clathrin light chain protein	plasma membrane
AT2G20890.1	PSB29, THF1	photosystem II reaction center PSB29 protein	plastid

AT2G20990.1	SYTA, NTMC2TYPE1.1, ATSYTA, NTMC2T1.1, SYT1	synaptotagmin A	plasma membrane,endoplasmic reticulum
AT2G21160.1	Translocon-associated protein (TRAP), alpha subunit	Translocon-associated protein (TRAP), alpha subunit	endoplasmic reticulum
AT2G21280.1	GC1, ATSULA, SULA	NAD(P)-binding Rossmann-fold superfamily protein	plastid
AT2G21330.1	FBA1	fructose-bisphosphate aldolase 1	plastid
AT2G21390.1	Coatomer, alpha subunit	Coatomer, alpha subunit	cytosol
AT2G21410.1	VHA-A2	vacuolar proton ATPase A2	vacuole
AT2G21530.1	SMAD/FHA domain-containing protein	SMAD/FHA domain-containing protein	plastid
AT2G21580.2	Ribosomal protein S25 family protein	Ribosomal protein S25 family protein	nucleus
AT2G21660.1	ATGRP7, CCR2, GR-RBP7, GRP7	cold, circadian rhythm, and rna binding 2	nucleus
AT2G21870.1	MGP1	copper ion binding;cobalt ion binding;zinc ion binding	mitochondrion
AT2G21960.1	unknown protein;LOCATED IN: chloroplast;EXPRESSED IN: 22 plant structures;EXPRESSED DURING: 13 growth stages;BEST Arabidopsis thaliana protein match is: unknown protein (TAIR:AT1G56180.1);Has 224 Blast hits to 222 proteins in	unknown protein;LOCATED IN: chloroplast;EXPRESSED IN: 22 plant structures;EXPRESSED DURING: 13 growth stages;BEST Arabidopsis thaliana protein match is: unknown protein (TAIR:AT1G56180.1);Has 224 Blast hits to 222 proteins in	plastid
AT2G22170.1	Lipase/lipooxygenase, PLAT/LH2 family protein	Lipase/lipooxygenase, PLAT/LH2 family protein	extracellular
AT2G22360.1	DNAJ heat shock family protein	DNAJ heat shock family protein	plastid
AT2G23670.1	YCF37	homolog of Synechocystis YCF37	plastid
AT2G24020.2	Uncharacterised BCR, YbaB family COG0718	Uncharacterised BCR, YbaB family COG0718	plastid
AT2G24060.1	Translation initiation factor 3 protein	Translation initiation factor 3 protein	plastid
AT2G24090.1	Ribosomal protein L35	Ribosomal protein L35	plastid
AT2G24420.2	DNA repair ATPase-related	DNA repair ATPase-related	extracellular
AT2G24590.1	RSZ22a, At-RSZ22a	RNA recognition motif and CCHC-type zinc finger domains containing protein	nucleus
AT2G24820.1	TIC55-II	translocon at the inner envelope membrane of chloroplasts 55-II	plastid
AT2G25080.1	ATGPX1, GPX1	glutathione peroxidase 1	plastid
AT2G25110.1	SDF2, ATSDL, AtSDF2	stromal cell-derived factor 2-like protein precursor	endoplasmic reticulum
AT2G25670.2	BEST Arabidopsis thaliana protein match is: copper ion binding (TAIR:AT4G32610.1);Has	BEST Arabidopsis thaliana protein match is: copper ion binding (TAIR:AT4G32610.1);Has 43784 Blast hits to 26928 proteins in	nucleus

	43784 Blast hits to 26928 proteins in 1799 species: Archae - 86;Bacteria - 6347;Metazoa - 15971;Fungi - 5398;Plants - 1931;Viruses - 259;	1799 species: Archae - 86;Bacteria - 6347;Metazoa - 15971;Fungi - 5398;Plants - 1931;Viruses - 259;	
AT2G26080.1	AtGLDP2, GLDP2	glycine decarboxylase P-protein 2	mitochondrion
AT2G26250.1	FDH, KCS10	3-ketoacyl-CoA synthase 10	golgi
AT2G26340.1	unknown protein;FUNCTIONS IN: molecular_function unknown;INVOLVED IN: biological_process unknown;LOCATED IN: chloroplast thylakoid membrane, chloroplast thylakoid lumen, chloroplast;EXPRESSED IN: 22 plant structures;EXPRESSED	unknown protein;FUNCTIONS IN: molecular_function unknown;INVOLVED IN: biological_process unknown;LOCATED IN: chloroplast thylakoid membrane, chloroplast thylakoid lumen, chloroplast;EXPRESSED IN: 22 plant structures;EXPRESSED	plastid
AT2G27290.1	Protein of unknown function (DUF1279)	Protein of unknown function (DUF1279)	plastid
AT2G27530.2	PGY1	Ribosomal protein L1p/L10e family	cytosol
AT2G27680.1	NAD(P)-linked oxidoreductase superfamily protein	NAD(P)-linked oxidoreductase superfamily protein	plastid
AT2G27710.3	60S acidic ribosomal protein family	60S acidic ribosomal protein family	cytosol
AT2G27720.1	60S acidic ribosomal protein family	60S acidic ribosomal protein family	cytosol
AT2G27730.1	copper ion binding	copper ion binding	mitochondrion
AT2G28000.1	CPN60A, CH-CPN60A, SLP	chaperonin-60alpha	plastid
AT2G28430.1	unknown protein;Has 28 Blast hits to 28 proteins in 12 species: Archae - 0;Bacteria - 0;Metazoa - 0;Fungi - 0;Plants - 28;Viruses - 0;Other Eukaryotes - 0 (source: NCBI BLink).	unknown protein;Has 28 Blast hits to 28 proteins in 12 species: Archae - 0;Bacteria - 0;Metazoa - 0;Fungi - 0;Plants - 28;Viruses - 0;Other Eukaryotes - 0 (source: NCBI BLink).	mitochondrion
AT2G28800.4	ALB3	63 kDa inner membrane family protein	plastid
AT2G28900.1	OEP16, ATOEP16-L, ATOEP16-1, OEP16-1	outer plastid envelope protein 16-1	plastid
AT2G29550.1	TUB7	tubulin beta-7 chain	cytosol
AT2G29650.2	ANTR1	phosphate transporter 4;1	plastid
AT2G30390.1	FC2, FC-II, ATFC-II	ferrochelatase 2	plastid
AT2G30490.1	ATC4H, C4H, CYP73A5, REF3	cinnamate-4-hydroxylase	endoplasmic reticulum
AT2G30620.1	winged-helix DNA-binding transcription factor family protein	winged-helix DNA-binding transcription factor family protein	nucleus
AT2G30870.1	ATGSTF10, ERD13, ATGSTF4, GSTF10	glutathione S-transferase PHI 10	cytosol

AT2G30930.1	unknown protein;FUNCTIONS IN: molecular_function unknown;INVOLVED IN: biological_process unknown;LOCATED IN: chloroplast, plasma membrane, membrane;EXPRESSED IN: 24 plant structures;EXPRESSED DURING: 14 growth stages;BEST Ar	unknown protein;FUNCTIONS IN: molecular_function unknown;INVOLVED IN: biological_process unknown;LOCATED IN: chloroplast, plasma membrane, membrane;EXPRESSED IN: 24 plant structures;EXPRESSED DURING: 14 growth stages;BEST Ar	plasma membrane
AT2G30950.1	VAR2, FTSH2	FtsH extracellular protease family	plastid
AT2G30970.2	ASP1	aspartate aminotransferase 1	mitochondrion
AT2G31610.1	Ribosomal protein S3 family protein	Ribosomal protein S3 family protein	cytosol
AT2G32060.3	Ribosomal protein L7Ae/L30e/S12e/Gadd45 family protein	Ribosomal protein L7Ae/L30e/S12e/Gadd45 family protein	cytosol
AT2G32080.2	PUR ALPHA-1	purin-rich alpha 1	cytosol
AT2G32240.1	FUNCTIONS IN: molecular_function unknown;INVOLVED IN: response to cadmium ion;LOCATED IN: plasma membrane;EXPRESSED IN: 25 plant structures;EXPRESSED DURING: 13 growth stages;CONTAINS InterPro DOMAIN/s: Prefoldin (InterPro:IP	FUNCTIONS IN: molecular_function unknown;INVOLVED IN: response to cadmium ion;LOCATED IN: plasma membrane;EXPRESSED IN: 25 plant structures;EXPRESSED DURING: 13 growth stages;CONTAINS InterPro DOMAIN/s: Prefoldin (InterPro:IP	endoplasmic reticulum
AT2G32640.1	Lycopene beta/epsilon cyclase protein	Lycopene beta/epsilon cyclase protein	plastid
AT2G32920.1	ATPDIL2-3, PDI9, ATPDI9, PDIL2-3	PDI-like 2-3	endoplasmic reticulum
AT2G33040.1	ATP3	gamma subunit of Mt ATP synthase	mitochondrion
AT2G33150.1	PKT3, PED1, KAT2	peroxisomal 3-ketoacyl-CoA thiolase 3	peroxisome
AT2G33210.2	HSP60-2	heat shock protein 60-2	mitochondrion
AT2G33220.1	GRIM-19 protein	GRIM-19 protein	mitochondrion
AT2G33380.1	RD20, CLO-3	Caleosin-related family protein	cytosol
AT2G33450.1	Ribosomal L28 family	Ribosomal L28 family	plastid
AT2G33800.1	Ribosomal protein S5 family protein	Ribosomal protein S5 family protein	plastid
AT2G34420.1	LHB1B2, LHCB1.5	photosystem II light harvesting complex gene B1B2	plastid
AT2G34430.1	LHB1B1, LHCB1.4	light-harvesting chlorophyll-protein complex II subunit B1	plastid
AT2G34460.1	NAD(P)-binding Rossmann-fold superfamily protein	NAD(P)-binding Rossmann-fold superfamily protein	plastid

AT2G34480.1	Ribosomal protein L18ae/LX family protein	Ribosomal protein L18ae/LX family protein	cytosol
AT2G35410.1	RNA-binding (RRM/RBD/RNP motifs) family protein	RNA-binding (RRM/RBD/RNP motifs) family protein	plastid
AT2G35490.1	Plastid-lipid associated protein PAP / fibrillin family protein	Plastid-lipid associated protein PAP / fibrillin family protein	plastid
AT2G35660.1	CTF2A	FAD/NAD(P)-binding oxidoreductase family protein	mitochondrion
AT2G35880.1	TPX2 (targeting protein for Xklp2) protein family	TPX2 (targeting protein for Xklp2) protein family	cytosol
AT2G36145.1	unknown protein;FUNCTIONS IN: molecular_function unknown;INVOLVED IN: biological_process unknown;LOCATED IN: chloroplast thylakoid membrane, chloroplast stroma, chloroplast;EXPRESSED IN: 19 plant structures;EXPRESSED DURING:	unknown protein;FUNCTIONS IN: molecular_function unknown;INVOLVED IN: biological_process unknown;LOCATED IN: chloroplast thylakoid membrane, chloroplast stroma, chloroplast;EXPRESSED IN: 19 plant structures;EXPRESSED DURING:	plastid
AT2G36160.1	Ribosomal protein S11 family protein	Ribosomal protein S11 family protein	cytosol
AT2G36250.2	FTSZ2-1, ATFTSZ2-1	Tubulin/FtsZ family protein	plastid
AT2G36530.1	LOS2, ENO2	Enolase	cytosol
AT2G36830.1	GAMMA-TIP, TIP1;1, GAMMA-TIP1	gamma tonoplast intrinsic protein	vacuole
AT2G37170.1	PIP2B, PIP2;2	plasma membrane intrinsic protein 2	plasma membrane
AT2G37220.1	RNA-binding (RRM/RBD/RNP motifs) family protein	RNA-binding (RRM/RBD/RNP motifs) family protein	plastid
AT2G37270.2	ATRPS5B, RPS5B	ribosomal protein 5B	cytosol
AT3G53750.1	ACT3	actin 3	cytosol
AT2G37660.1	NAD(P)-binding Rossmann-fold superfamily protein	NAD(P)-binding Rossmann-fold superfamily protein	plastid
AT2G37860.3	LCD1	Protein of unknown function (DUF3411)	plastid
AT2G37970.1	SOUL-1	SOUL heme-binding family protein	plasma membrane
AT2G38040.2	CAC3	acetyl Co-enzyme a carboxylase carboxyltransferase alpha subunit	plastid
AT2G38270.1	CXIP2, ATGRX2	CAX-interacting protein 2	plastid
AT2G38280.2	FAC1, ATAMPD	AMP deaminase, putative / myoadenylate deaminase, putative	plasma membrane
AT2G38540.1	LP1, LTP1, ATLTP1	lipid transfer protein 1	extracellular
AT2G38550.1	Transmembrane proteins 14C	Transmembrane proteins 14C	plastid
AT2G39010.1	PIP2E, PIP2;6	plasma membrane intrinsic protein 2E	plasma membrane
AT3G55280.3	RPL23AB	ribosomal protein L23AB	cytosol
AT2G39470.1	PPL2	PsbP-like protein 2	plastid

AT2G39730.1	RCA	rubisco activase	plastid
AT2G39800.3	P5CS1	delta1-pyrroline-5-carboxylate synthase 1	cytosol
AT2G39990.1	EIF2, AteIF3f, eIF3F	eukaryotic translation initiation factor 2	nucleus,cytosol
AT2G40060.1	Clathrin light chain protein	Clathrin light chain protein	plasma membrane
AT2G40100.1	LHCB4.3	light harvesting complex photosystem II	plastid
AT2G40290.1	Eukaryotic translation initiation factor 2 subunit 1	Eukaryotic translation initiation factor 2 subunit 1	cytosol
AT2G40510.1	Ribosomal protein S26e family protein	Ribosomal protein S26e family protein	cytosol
AT2G40765.1	unknown protein;FUNCTIONS IN: molecular_function unknown;INVOLVED IN: biological_process unknown;LOCATED IN: mitochondrion, mitochondrial respiratory chain complex III;EXPRESSED IN: 24 plant structures;EXPRESSED DURING: 15 gr	unknown protein;FUNCTIONS IN: molecular_function unknown;INVOLVED IN: biological_process unknown;LOCATED IN: mitochondrion, mitochondrial respiratory chain complex III;EXPRESSED IN: 24 plant structures;EXPRESSED DURING: 15 gr	mitochondrion
AT2G40890.1	CYP98A3	cytochrome P450, family 98, subfamily A, polypeptide 3	endoplasmic reticulum
AT2G41100.4	TCH3	Calcium-binding EF hand family protein	cytosol
AT2G41560.1	ACA4	autoinhibited Ca(2+)-ATPase, isoform 4	vacuole
AT2G41740.1	VLN2, ATVLN2	villin 2	cytosol
AT2G41840.1	Ribosomal protein S5 family protein	Ribosomal protein S5 family protein	cytosol
AT2G42130.3	Plastid-lipid associated protein PAP / fibrillin family protein	Plastid-lipid associated protein PAP / fibrillin family protein	plastid
AT2G42210.4	ATOEP16-3, OEP16-3	Mitochondrial import inner membrane translocase subunit Tim17/Tim22/Tim23 family protein	mitochondrion
AT2G42220.1	Rhodanese/Cell cycle control phosphatase superfamily protein	Rhodanese/Cell cycle control phosphatase superfamily protein	plastid
AT2G42520.1	P-loop containing nucleoside triphosphate hydrolases superfamily protein	P-loop containing nucleoside triphosphate hydrolases superfamily protein	nucleus
AT2G42590.3	GRF9, GF14 MU	general regulatory factor 9	cytosol
AT2G42600.2	ATPPC2, PPC2	phosphoenolpyruvate carboxylase 2	cytosol
AT2G42690.1	alpha/beta-Hydrolases superfamily protein	alpha/beta-Hydrolases superfamily protein	cytosol
AT5G45775.1	Ribosomal L5P family protein	Ribosomal L5P family protein	cytosol
AT2G43030.1	Ribosomal protein L3 family protein	Ribosomal protein L3 family protein	plastid

AT3G59540.1	Ribosomal L38e protein family	Ribosomal L38e protein family	cytosol
AT2G43950.1	OEP37, ATOEP37	chloroplast outer envelope protein 37	plastid
AT2G44060.2	Late embryogenesis abundant protein, group 2	Late embryogenesis abundant protein, group 2	cytosol
AT2G44100.2	ATGDI1, AT-GDI1, GDI1	guanosine nucleotide diphosphate dissociation inhibitor 1	cytosol
AT2G44120.1	Ribosomal protein L30/L7 family protein	Ribosomal protein L30/L7 family protein	cytosol
AT2G44350.2	ATCS, CSY4	Citrate synthase family protein	mitochondrion
AT2G44610.1	RAB6, ATRABH1B, ATRAB6A, RAB6A	Ras-related small GTP-binding family protein	golgi
AT2G44640.1	FUNCTIONS IN: molecular_function unknown;INVOLVED IN: biological_process unknown;LOCATED IN: mitochondrion, chloroplast, plasma membrane, plastid, chloroplast envelope;EXPRESSED IN: 23 plant structures;EXPRESSED DURING: 13 gro	FUNCTIONS IN: molecular_function unknown;INVOLVED IN: biological_process unknown;LOCATED IN: mitochondrion, chloroplast, plasma membrane, plastid, chloroplast envelope;EXPRESSED IN: 23 plant structures;EXPRESSED DURING: 13 gro	plastid
AT2G45140.1	PVA12	plant VAP homolog 12	endoplasmic reticulum
AT2G45470.1	FLA8, AGP8	FASCICLIN-like arabinogalactan protein 8	plasma membrane,extracellular
AT2G45640.1	SAP18, ATSAP18	SIN3 associated polypeptide P18	nucleus
AT2G45710.1	Zinc-binding ribosomal protein family protein	Zinc-binding ribosomal protein family protein	cytosol
AT2G45740.3	PEX11D	peroxin 11D	peroxisome
AT2G45820.1	Remorin family protein	Remorin family protein	plasma membrane
AT2G45960.2	PIP1B, TMP-A, ATHH2, PIP1;2	plasma membrane intrinsic protein 1B	plasma membrane
AT2G46280.2	TRIP-1, TIF3I1	TGF-beta receptor interacting protein 1	cytosol
AT2G46820.2	PTAC8, TMP14, PSAP, PSI-P	photosystem I P subunit	plastid
AT3G62250.1	UBQ5	ubiquitin 5	nucleus
AT2G47470.1	ATPDIL2-1, UNE5, MEE30, PDI11, ATPDI11	thioredoxin family protein	endoplasmic reticulum
AT2G47510.2	FUM1	fumarase 1	mitochondrion
AT3G62840.1	Small nuclear ribonucleoprotein family protein	Small nuclear ribonucleoprotein family protein	nucleus
AT2G47730.1	ATGSTF8, ATGSTF5, GST6, GSTF8	glutathione S-transferase phi 8	plastid
AT2G47840.1	Uncharacterised conserved protein ycf60	Uncharacterised conserved protein ycf60	plastid
AT3G01280.1	VDAC1, ATVDAC1	voltage dependent anion channel 1	mitochondrion

AT3G01290.1	SPFH/Band 7/PHB domain-containing membrane-associated protein family	SPFH/Band 7/PHB domain-containing membrane-associated protein family	plasma membrane
AT3G01390.2	VMA10, AVMA10	vacuolar membrane ATPase 10	vacuole
AT3G01440.1	PQL1, PQL2	PsbQ-like 1	plastid
AT3G01480.1	CYP38, ATCYP38	cyclophilin 38	plastid
AT3G01500.1	CA1, ATBCA1, SABP3, ATSABP3	carbonic anhydrase 1	plastid
AT3G02080.1	Ribosomal protein S19e family protein	Ribosomal protein S19e family protein	cytosol
AT3G02090.1	MPPBETA	Insulinase (Peptidase family M16) protein	mitochondrion
AT3G02200.2	Proteasome component (PCI) domain protein	Proteasome component (PCI) domain protein	nucleus
AT3G02260.1	BIG, DOC1, TIR3, UMB1, ASA1, LPR1, CRM1	auxin transport protein (BIG)	nucleus
AT3G02560.2	Ribosomal protein S7e family protein	Ribosomal protein S7e family protein	cytosol
AT3G02830.1	ZFN1	zinc finger protein 1	nucleus
AT3G02880.1	Leucine-rich repeat protein kinase family protein	Leucine-rich repeat protein kinase family protein	plasma membrane
AT3G03100.1	NADH:ubiquinone oxidoreductase, 17.2kDa subunit	NADH:ubiquinone oxidoreductase, 17.2kDa subunit	mitochondrion
AT3G03250.1	UGP, UGP1, AtUGP1	UDP-GLUCOSE PYROPHOSPHORYLASE 1	cytosol
AT3G03710.1	RIF10, PNP	polyribonucleotide nucleotidyltransferase, putative	plastid
AT3G04120.1	GAPC, GAPC-1, GAPC1	glyceraldehyde-3-phosphate dehydrogenase C subunit 1	nucleus,cytosol
AT3G04260.1	PTAC3	plastid transcriptionally active 3	plastid
AT3G04340.1	emb2458	FtsH extracellular protease family	plastid
AT3G04790.1	Ribose 5-phosphate isomerase, type A protein	Ribose 5-phosphate isomerase, type A protein	plastid
AT3G04840.1	Ribosomal protein S3Ae	Ribosomal protein S3Ae	cytosol
AT3G04870.2	ZDS, PDE181, SPC1	zeta-carotene desaturase	plastid
AT3G04920.1	Ribosomal protein S24e family protein	Ribosomal protein S24e family protein	cytosol
AT3G05060.1	NOP56-like pre RNA processing ribonucleoprotein	NOP56-like pre RNA processing ribonucleoprotein	nucleus
AT3G05230.1	Signal peptidase subunit	Signal peptidase subunit	endoplasmic reticulum
AT3G05420.1	ACBP4	acyl-CoA binding protein 4	cytosol
AT3G05530.1	RPT5A, ATS6A.2	regulatory particle triple-A ATPase 5A	nucleus,cytosol
AT3G05560.3	Ribosomal L22e protein family	Ribosomal L22e protein family	cytosol
AT3G05590.1	RPL18	ribosomal protein L18	cytosol
AT3G05970.1	LACS6, ATLACS6	long-chain acyl-CoA synthetase 6	peroxisome
AT3G06050.1	PRXIIF, ATPRXIIF	peroxiredoxin IIF	mitochondrion

AT3G06300.1	AT-P4H-2	P4H isoform 2	endoplasmic reticulum
AT3G06510.1	SFR2, ATSFR2	Glycosyl hydrolase superfamily protein	plastid
AT3G06720.2	AT-IMP, ATKAP ALPHA, AIMP ALPHA	importin alpha isoform 1	cytosol
AT3G06730.1	TRX P, TRX z	Thioredoxin z	plastid
AT3G06810.1	IBR3	acyl-CoA dehydrogenase-related	peroxisome
AT3G07110.1	Ribosomal protein L13 family protein	Ribosomal protein L13 family protein	cytosol
AT3G07480.1	2Fe-2S ferredoxin-like superfamily protein	2Fe-2S ferredoxin-like superfamily protein	mitochondrion
AT3G07568.1	unknown protein;Has 9 Blast hits to 9 proteins in 5 species: Archae - 0;Bacteria - 0;Metazoa - 0;Fungi - 0;Plants - 9;Viruses - 0;Other Eukaryotes - 0 (source: NCBI BLink).	unknown protein;Has 9 Blast hits to 9 proteins in 5 species: Archae - 0;Bacteria - 0;Metazoa - 0;Fungi - 0;Plants - 9;Viruses - 0;Other Eukaryotes - 0 (source: NCBI BLink).	mitochondrion
AT3G07770.1	Hsp89.1, AtHsp90.6, AtHsp90-6	HEAT SHOCK PROTEIN 89.1	mitochondrion
AT3G08530.1	Clathrin, heavy chain	Clathrin, heavy chain	plasma membrane
AT3G08580.2	AAC1	ADP/ATP carrier 1	mitochondrion
AT3G08920.1	Rhodanese/Cell cycle control phosphatase superfamily protein	Rhodanese/Cell cycle control phosphatase superfamily protein	plastid
AT3G08940.2	LHCB4.2	light harvesting complex photosystem II	plastid
AT3G09050.1	unknown protein;FUNCTIONS IN: molecular_function unknown;INVOLVED IN: biological_process unknown;LOCATED IN: chloroplast thylakoid membrane, chloroplast;EXPRESSED IN: 21 plant structures;EXPRESSED DURING: 13 growth stages;Ha	unknown protein;FUNCTIONS IN: molecular_function unknown;INVOLVED IN: biological_process unknown;LOCATED IN: chloroplast thylakoid membrane, chloroplast;EXPRESSED IN: 21 plant structures;EXPRESSED DURING: 13 growth stages;Ha	plastid
AT3G09200.1	Ribosomal protein L10 family protein	Ribosomal protein L10 family protein	cytosol
AT3G09440.2	Heat shock protein 70 (Hsp 70) family protein	Heat shock protein 70 (Hsp 70) family protein	peroxisome
AT3G09500.1	Ribosomal L29 family protein	Ribosomal L29 family protein	cytosol
AT3G09580.1	FAD/NAD(P)-binding oxidoreductase family protein	FAD/NAD(P)-binding oxidoreductase family protein	plastid
AT3G09630.1	Ribosomal protein L4/L1 family	Ribosomal protein L4/L1 family	cytosol
AT3G09740.1	SYP71, ATSYP71	syntaxin of plants 71	plasma membrane
AT3G09840.1	CDC48, ATCDC48, CDC48A	cell division cycle 48	cytosol
AT3G10060.1	FKBP-like peptidyl-prolyl cis-trans isomerase family protein	FKBP-like peptidyl-prolyl cis-trans isomerase family protein	plastid

AT3G10130.1	SOUL heme-binding family protein	SOUL heme-binding family protein	plastid
AT3G10350.1	P-loop containing nucleoside triphosphate hydrolases superfamily protein	P-loop containing nucleoside triphosphate hydrolases superfamily protein	plastid
AT3G10370.1	SDP6	FAD-dependent oxidoreductase family protein	mitochondrion
AT3G10380.1	SEC8, ATSEC8	subunit of exocyst complex 8	plasma membrane
AT3G10690.1	GYRA	DNA GYRASE A	plastid
AT5G05370.1	Cytochrome b-c1 complex, subunit 8 protein	Cytochrome b-c1 complex, subunit 8 protein	mitochondrion
AT3G10920.1	MSD1, MEE33, ATMSD1	manganese superoxide dismutase 1	mitochondrion
AT3G11070.1	Outer membrane OMP85 family protein	Outer membrane OMP85 family protein	mitochondrion
AT3G11130.1	Clathrin, heavy chain	Clathrin, heavy chain	plasma membrane
AT3G11400.1	EIF3G1, ATEIF3G1	eukaryotic translation initiation factor 3G1	cytosol
AT3G11510.1	Ribosomal protein S11 family protein	Ribosomal protein S11 family protein	cytosol
AT3G11630.1	Thioredoxin superfamily protein	Thioredoxin superfamily protein	plastid
AT3G11780.1	MD-2-related lipid recognition domain-containing protein / ML domain-containing protein	MD-2-related lipid recognition domain-containing protein / ML domain-containing protein	extracellular
AT3G11910.2	UBP13	ubiquitin-specific protease 13	cytosol
AT3G11930.1	Adenine nucleotide alpha hydrolases-like superfamily protein	Adenine nucleotide alpha hydrolases-like superfamily protein	nucleus
AT3G11940.2	ATRPS5A, AML1, RPS5A	ribosomal protein 5A	cytosol
AT3G11945.1	PDS2, ATHST, HST	homogentisate prenyltransferase	plastid
AT3G12345.1	unknown protein;LOCATED IN: chloroplast;Has 35333 Blast hits to 34131 proteins in 2444 species: Archae - 798;Bacteria - 22429;Metazoa - 974;Fungi - 991;Plants - 531;Viruses - 0;Other Eukaryotes - 9610 (source: NCBI BLink).	unknown protein;LOCATED IN: chloroplast;Has 35333 Blast hits to 34131 proteins in 2444 species: Archae - 798;Bacteria - 22429;Metazoa - 974;Fungi - 991;Plants - 531;Viruses - 0;Other Eukaryotes - 9610 (source: NCBI BLink).	plastid
AT3G12390.1	Nascent polypeptide-associated complex (NAC), alpha subunit family protein	Nascent polypeptide-associated complex (NAC), alpha subunit family protein	nucleus
AT3G12780.1	PGK1	phosphoglycerate kinase 1	plastid
AT3G13120.2	Ribosomal protein S10p/S20e family protein	Ribosomal protein S10p/S20e family protein	plastid
AT3G13300.2	VCS	Transducin/WD40 repeat-like superfamily protein	cytosol
AT3G13870.1	RHD3	Root hair defective 3 GTP-binding protein (RHD3)	endoplasmic reticulum,golgi

AT3G13920.1	EIF4A1, RH4, TIF4A1	eukaryotic translation initiation factor 4A1	cytosol
AT3G13930.1	Dihydrolipoamide acetyltransferase, long form protein	Dihydrolipoamide acetyltransferase, long form protein	mitochondrion
AT3G14110.2	FLU	Tetratricopeptide repeat (TPR)-like superfamily protein	vacuole
AT3G14210.1	ESM1	epithiospecifier modifier 1	vacuole
AT3G14310.1	ATPME3, PME3	pectin methylesterase 3	extracellular
AT3G14415.1	Aldolase-type TIM barrel family protein	Aldolase-type TIM barrel family protein	peroxisome
AT3G14420.2	Aldolase-type TIM barrel family protein	Aldolase-type TIM barrel family protein	peroxisome
AT3G15090.1	GroES-like zinc-binding alcohol dehydrogenase family protein	GroES-like zinc-binding alcohol dehydrogenase family protein	mitochondrion
AT3G15110.1	unknown protein;FUNCTIONS IN: molecular_function unknown;INVOLVED IN: biological_process unknown;LOCATED IN: chloroplast thylakoid membrane;EXPRESSED IN: 20 plant structures;EXPRESSED DURING: 13 growth stages;CONTAINS InterP	unknown protein;FUNCTIONS IN: molecular_function unknown;INVOLVED IN: biological_process unknown;LOCATED IN: chloroplast thylakoid membrane;EXPRESSED IN: 20 plant structures;EXPRESSED DURING: 13 growth stages;CONTAINS InterP	plastid
AT3G15190.1	chloroplast 30S ribosomal protein S20, putative	chloroplast 30S ribosomal protein S20, putative	plastid
AT3G15360.1	ATHM4, TRX-M4, ATM4	thioredoxin M-type 4	plastid
AT3G15520.1	Cyclophilin-like peptidyl-prolyl cis-trans isomerase family protein	Cyclophilin-like peptidyl-prolyl cis-trans isomerase family protein	plastid
AT3G15730.1	PLDALPHA1, PLD	phospholipase D alpha 1	cytosol
AT3G15980.1	Coatomer, beta subunit	Coatomer, beta subunit	cytosol
AT3G16000.1	MFP1	MAR binding filament-like protein 1	plastid
AT3G16140.1	PSAH-1	photosystem I subunit H-1	plastid
AT3G16480.1	MPPalpha	mitochondrial processing peptidase alpha subunit	mitochondrion
AT3G16640.1	TCTP	translationally controlled tumor protein	cytosol
AT3G16780.1	Ribosomal protein L19e family protein	Ribosomal protein L19e family protein	cytosol
AT3G17020.1	Adenine nucleotide alpha hydrolases-like superfamily protein	Adenine nucleotide alpha hydrolases-like superfamily protein	plasma membrane,cytosol
AT3G17240.1	mtLPD2	lipoamide dehydrogenase 2	mitochondrion
AT3G17930.1	unknown protein;FUNCTIONS IN: molecular_function unknown;INVOLVED IN: biological_process	unknown protein;FUNCTIONS IN: molecular_function unknown;INVOLVED IN: biological_process	plastid

	unknown;LOCATED IN: chloroplast thylakoid membrane;EXPRESSED IN: 22 plant structures;EXPRESSED DURING: 13 growth stages;CONTAINS InterP	unknown;LOCATED IN: chloroplast thylakoid membrane;EXPRESSED IN: 22 plant structures;EXPRESSED DURING: 13 growth stages;CONTAINS InterP	
AT3G17970.1	atToc64-III, TOC64-III	translocon at the outer membrane of chloroplasts 64-III	plastid
AT3G18035.1	HON4	winged-helix DNA-binding transcription factor family protein	nucleus
AT3G18740.1	Ribosomal protein L7Ae/L30e/S12e/Gadd45 family protein	Ribosomal protein L7Ae/L30e/S12e/Gadd45 family protein	cytosol
AT3G18780.2	ACT2, DER1, LSR2, ENL2	actin 2	cytosol
AT3G18820.1	ATRABG3F, ATRAB7B, RAB71, RABG3F, RAB7B	RAB GTPase homolog G3F	golgi
AT3G18890.1	NAD(P)-binding Rossmann-fold superfamily protein	NAD(P)-binding Rossmann-fold superfamily protein	plastid
AT3G19170.1	ATPREP1, ATZNMP, PREP1	presequence protease 1	mitochondrion,plastid
AT3G19420.1	ATPEN2, PEN2	PTEN 2	cytosol
AT3G19760.1	EIF4A-III	eukaryotic initiation factor 4A-III	nucleus
AT3G19820.3	DWF1	cell elongation protein / DWARF1 / DIMINUTO (DIM)	plasma membrane,cytosol
AT3G20000.1	TOM40	translocase of the outer mitochondrial membrane 40	mitochondrion
AT3G20320.1	TGD2	trigalactosyldiacylglycerol2	plastid
AT3G20390.1	endoribonuclease L-PSP family protein	endoribonuclease L-PSP family protein	plastid
AT3G20820.1	Leucine-rich repeat (LRR) family protein	Leucine-rich repeat (LRR) family protein	extracellular
AT3G21055.1	PSBTN	photosystem II subunit T	plastid
AT3G22200.1	POP2, GABA-T, HER1	Pyridoxal phosphate (PLP)-dependent transferases superfamily protein	mitochondrion
AT3G22230.1	Ribosomal L27e protein family	Ribosomal L27e protein family	cytosol
AT3G22845.1	emp24/gp25L/p24 family/GOLD family protein	emp24/gp25L/p24 family/GOLD family protein	endoplasmic reticulum
AT3G23300.1	S-adenosyl-L-methionine-dependent methyltransferases superfamily protein	S-adenosyl-L-methionine-dependent methyltransferases superfamily protein	golgi
AT3G23400.1	FIB4	Plastid-lipid associated protein PAP / fibrillin family protein	plastid
AT3G23990.1	HSP60, HSP60-3B	heat shock protein 60	mitochondrion
AT3G24590.1	PLSP1	plastidic type i signal peptidase 1	plastid
AT3G24830.1	Ribosomal protein L13 family protein	Ribosomal protein L13 family protein	cytosol
AT3G25520.1	ATL5, PGY3, OLI5, RPL5A	ribosomal protein L5	cytosol
AT3G25690.1	CHUP1	Hydroxyproline-rich glycoprotein family protein	plastid

AT3G25760.1	AOC1, ERD12	allene oxide cyclase 1	plastid
AT3G25770.1	AOC2	allene oxide cyclase 2	plastid
AT3G25800.1	PDF1, PR 65, PP2AA2	protein phosphatase 2A subunit A2	cytosol
AT3G25860.1	LTA2, PLE2	2-oxoacid dehydrogenases acyltransferase family protein	plastid
AT3G25920.1	RPL15	ribosomal protein L15	plastid
AT3G26060.1	ATPRX Q	Thioredoxin superfamily protein	plastid
AT3G26070.1	Plastid-lipid associated protein PAP / fibrillin family protein	Plastid-lipid associated protein PAP / fibrillin family protein	plastid
AT3G26520.1	TIP2, SITIP, GAMMA-TIP2, TIP1;2	tonoplast intrinsic protein 2	vacuole
AT3G26650.1	GAPA, GAPA-1	glyceraldehyde 3-phosphate dehydrogenase A subunit	plastid
AT3G26710.1	CCB1	cofactor assembly of complex C	plastid
AT3G26740.1	CCL	CCR-like	plastid
AT3G27240.1	Cytochrome C1 family	Cytochrome C1 family	mitochondrion
AT3G27380.2	SDH2-1	succinate dehydrogenase 2-1	mitochondrion
AT3G27820.1	ATMDAR4, MDAR4	monodehydroascorbate reductase 4	plastid,peroxisome
AT3G27850.1	RPL12-C	ribosomal protein L12-C	plastid
AT3G27925.1	DEGP1, Deg1	DegP protease 1	plastid
AT3G28710.1	ATPase, V0/A0 complex, subunit C/D	ATPase, V0/A0 complex, subunit C/D	vacuole
AT3G42050.1	vacuolar ATP synthase subunit H family protein	vacuolar ATP synthase subunit H family protein	vacuole,golgi
AT3G42170.1	BED zinc finger;hAT family dimerisation domain	BED zinc finger;hAT family dimerisation domain	nucleus
AT3G43300.2	ATMIN7	HOPM interactor 7	cytosol
AT3G43520.1	Transmembrane proteins 14C	Transmembrane proteins 14C	plastid
AT3G44110.1	ATJ3, ATJ	DNAJ homologue 3	nucleus
AT3G44310.1	NIT1, ATNIT1, NITI	nitrilase 1	cytosol
AT3G44320.1	NIT3, AtNIT3	nitrilase 3	cytosol
AT3G44330.1	INVOLVED IN: protein processing;LOCATED IN: mitochondrion, endoplasmic reticulum, plasma membrane, vacuole;EXPRESSED IN: 25 plant structures;EXPRESSED DURING: 13 growth stages;CONTAINS InterPro DOMAIN/s: Nicalin (InterPro:IPR0	INVOLVED IN: protein processing;LOCATED IN: mitochondrion, endoplasmic reticulum, plasma membrane, vacuole;EXPRESSED IN: 25 plant structures;EXPRESSED DURING: 13 growth stages;CONTAINS InterPro DOMAIN/s: Nicalin (InterPro:IPR0	endoplasmic reticulum
AT3G44880.1	ACD1, LLS1, PAO	Pheophorbide a oxygenase family protein with Rieske [2Fe-2S] domain	plastid
AT3G44890.1	RPL9	ribosomal protein L9	plastid

AT5G62300.2	Ribosomal protein S10p/S20e family protein	Ribosomal protein S10p/S20e family protein	cytosol
AT3G45140.1	LOX2, ATLOX2	lipoxygenase 2	plastid
AT3G45780.2	PHOT1, NPH1, JK224, RPT1	phototropin 1	plastid
AT3G46060.3	ARA3	RAB GTPase homolog 8A	cytosol
AT5G59613.2	unknown protein;FUNCTIONS IN: molecular_function unknown;INVOLVED IN: biological_process unknown;LOCATED IN: mitochondrial respiratory chain complex III;BEST Arabidopsis thaliana protein match is: unknown protein (TAIR:AT3G464	unknown protein;FUNCTIONS IN: molecular_function unknown;INVOLVED IN: biological_process unknown;LOCATED IN: mitochondrial respiratory chain complex III;BEST Arabidopsis thaliana protein match is: unknown protein (TAIR:AT3G464	mitochondrion
AT3G46740.1	TOC75-III, MAR1	translocon at the outer envelope membrane of chloroplasts 75-III	plastid
AT3G46780.1	PTAC16	plastid transcriptionally active 16	plastid
AT3G46970.1	ATPHS2, PHS2	alpha-glucan phosphorylase 2	cytosol
AT3G47070.1	LOCATED IN: thylakoid, chloroplast thylakoid membrane, chloroplast, chloroplast envelope;EXPRESSED IN: 22 plant structures;EXPRESSED DURING: 13 growth stages;CONTAINS InterPro DOMAIN/s: Thylakoid soluble phosphoprotein TSP9 (In	LOCATED IN: thylakoid, chloroplast thylakoid membrane, chloroplast, chloroplast envelope;EXPRESSED IN: 22 plant structures;EXPRESSED DURING: 13 growth stages;CONTAINS InterPro DOMAIN/s: Thylakoid soluble phosphoprotein TSP9 (In	plastid
AT3G47370.3	Ribosomal protein S10p/S20e family protein	Ribosomal protein S10p/S20e family protein	cytosol
AT3G47470.1	LHCA4, CAB4	light-harvesting chlorophyll-protein complex I subunit A4	plastid
AT3G47520.1	MDH	malate dehydrogenase	plastid
AT3G47860.1	CHL	chloroplastic lipocalin	plastid
AT3G47930.1	ATGLDH, GLDH	L-galactono-1,4-lactone dehydrogenase	mitochondrion
AT3G48000.1	ALDH2B4, ALDH2, ALDH2A	aldehyde dehydrogenase 2B4	mitochondrion
AT3G48140.1	B12D protein	B12D protein	peroxisome
AT3G48500.1	PDE312, PTAC10	Nucleic acid-binding, OB-fold-like protein	plastid
AT3G48730.1	GSA2	glutamate-1-semialdehyde 2,1-aminomutase 2	plastid
AT3G48890.1	ATMP2, ATMAPR3, MSBP2, MAPR3	membrane-associated progesterone binding protein 3	endoplasmic reticulum
AT3G48930.1	EMB1080	Nucleic acid-binding, OB-fold-like protein	cytosol
AT3G49010.4	ATBBC1, BBC1, RSU2	breast basic conserved 1	cytosol
AT3G49430.2	SRp34a, SR34a, At-SR34a	SER/ARG-rich protein 34A	nucleus

AT3G49470.1	NACA2	nascent polypeptide-associated complex subunit alpha-like protein 2	nucleus
AT3G49720.2	unknown protein;FUNCTIONS IN: molecular_function unknown;INVOLVED IN: biological_process unknown;LOCATED IN: chloroplast thylakoid membrane, Golgi apparatus, plasma membrane, membrane;EXPRESSED IN: 25 plant structures;EXPRESS	unknown protein;FUNCTIONS IN: molecular_function unknown;INVOLVED IN: biological_process unknown;LOCATED IN: chloroplast thylakoid membrane, Golgi apparatus, plasma membrane, membrane;EXPRESSED IN: 25 plant structures;EXPRESS	golgi
AT3G49870.1	ATARLA1C, ARLA1C	ADP-ribosylation factor-like A1C	cytosol
AT3G49910.1	Translation protein SH3-like family protein	Translation protein SH3-like family protein	cytosol
AT3G50500.2	SNRK2.2	SNF1-related protein kinase 2.2	cytosol
AT3G50590.1	Transducin/WD40 repeat-like superfamily protein	Transducin/WD40 repeat-like superfamily protein	plasma membrane,nucleus
AT3G50670.1	U1-70K, U1SNRNP	U1 small nuclear ribonucleoprotein-70K	nucleus
AT3G51140.1	Protein of unknown function (DUF3353)	Protein of unknown function (DUF3353)	plastid
AT3G51420.1	SSL4, ATSSL4	strictosidine synthase-like 4	plasma membrane
AT3G51800.3	ATG2	metallopeptidase M24 family protein	cytosol
AT3G51820.1	ATG4, G4, CHLG	UbiA prenyltransferase family protein	plastid
AT3G52140.1	tetratricopeptide repeat (TPR)-containing protein	tetratricopeptide repeat (TPR)-containing protein	cytosol
AT3G52150.2	RNA-binding (RRM/RBD/RNP motifs) family protein	RNA-binding (RRM/RBD/RNP motifs) family protein	plastid
AT3G52200.1	LTA3	Dihydrolipoamide acetyltransferase, long form protein	mitochondrion
AT3G52230.1	unknown protein;FUNCTIONS IN: molecular_function unknown;INVOLVED IN: biological_process unknown;LOCATED IN: chloroplast outer membrane, chloroplast thylakoid membrane, chloroplast, chloroplast envelope;EXPRESSED IN: 24 plant	unknown protein;FUNCTIONS IN: molecular_function unknown;INVOLVED IN: biological_process unknown;LOCATED IN: chloroplast outer membrane, chloroplast thylakoid membrane, chloroplast, chloroplast envelope;EXPRESSED IN: 24 plant	plastid
AT3G52300.1	ATPQ	ATP synthase D chain, mitochondrial	mitochondrion
AT3G52380.1	CP33, PDE322	chloroplast RNA-binding protein 33	plastid

AT3G52730.1	ubiquinol-cytochrome C reductase UQCRX/QCR9-like family protein	ubiquinol-cytochrome C reductase UQCRX/QCR9-like family protein	mitochondrion
AT3G52880.1	ATMDAR1, MDAR1	monodehydroascorbate reductase 1	peroxisome
AT3G52930.1	Aldolase superfamily protein	Aldolase superfamily protein	cytosol
AT3G52950.1	CBS / octicosapeptide/Phox/Bemp1 (PB1) domains-containing protein	CBS / octicosapeptide/Phox/Bemp1 (PB1) domains-containing protein	mitochondrion
AT3G52960.1	Thioredoxin superfamily protein	Thioredoxin superfamily protein	plastid
AT3G53020.1	STV1, RPL24B, RPL24	Ribosomal protein L24e family protein	cytosol
AT3G53110.1	LOS4	P-loop containing nucleoside triphosphate hydrolases superfamily protein	nucleus,cytosol
AT3G53130.1	LUT1, CYP97C1	Cytochrome P450 superfamily protein	plastid
AT3G53420.2	PIP2A, PIP2, PIP2;1	plasma membrane intrinsic protein 2A	plasma membrane
AT3G53430.1	Ribosomal protein L11 family protein	Ribosomal protein L11 family protein	cytosol
AT3G53470.1	unknown protein;FUNCTIONS IN: molecular_function unknown;INVOLVED IN: biological_process unknown;LOCATED IN: chloroplast thylakoid membrane, chloroplast;EXPRESSED IN: 22 plant structures;EXPRESSED DURING: 13 growth stages;Ha	unknown protein;FUNCTIONS IN: molecular_function unknown;INVOLVED IN: biological_process unknown;LOCATED IN: chloroplast thylakoid membrane, chloroplast;EXPRESSED IN: 22 plant structures;EXPRESSED DURING: 13 growth stages;Ha	plastid
AT3G53740.1	Ribosomal protein L36e family protein	Ribosomal protein L36e family protein	cytosol
AT3G53990.1	Adenine nucleotide alpha hydrolases-like superfamily protein	Adenine nucleotide alpha hydrolases-like superfamily protein	plasma membrane,cytosol
AT3G54110.1	ATPUMP1, UCP, PUMP1, ATUCP1, UCP1	plant uncoupling mitochondrial protein 1	mitochondrion
AT3G54210.1	Ribosomal protein L17 family protein	Ribosomal protein L17 family protein	plastid
AT3G54540.1	ATGCN4, GCN4	general control non-repressible 4	nucleus
AT3G54890.4	LHCA1	photosystem I light harvesting complex gene 1	plastid
AT3G55220.1	Cleavage and polyadenylation specificity factor (CPSF) A subunit protein	Cleavage and polyadenylation specificity factor (CPSF) A subunit protein	nucleus
AT3G55330.1	PPL1	PsbP-like protein 1	plastid

AT3G55410.1	2-oxoglutarate dehydrogenase, E1 component	2-oxoglutarate dehydrogenase, E1 component	mitochondrion
AT3G55440.1	ATCTIMC, TPI, CYTOTPI	triosephosphate isomerase	cytosol
AT3G55800.1	SBPASE	sedoheptulose-bisphosphatase	plastid
AT3G56150.2	EIF3C	eukaryotic translation initiation factor 3C	nucleus
AT3G56190.1	ALPHA-SNAP2, ASNAP	alpha-soluble NSF attachment protein 2	vacuole
AT3G56310.1	Melibiase family protein	Melibiase family protein	extracellular
AT3G56340.1	Ribosomal protein S26e family protein	Ribosomal protein S26e family protein	cytosol
AT3G56430.1	unknown protein;FUNCTIONS IN: molecular_function unknown;INVOLVED IN: biological_process unknown;LOCATED IN: mitochondrion, vacuole;EXPRESSED IN: 22 plant structures;EXPRESSED DURING: 13 growth stages;BEST Arabidopsis thalia	unknown protein;FUNCTIONS IN: molecular_function unknown;INVOLVED IN: biological_process unknown;LOCATED IN: mitochondrion, vacuole;EXPRESSED IN: 22 plant structures;EXPRESSED DURING: 13 growth stages;BEST Arabidopsis thalia	mitochondrion
AT3G56650.1	Mog1/PsbP/DUF1795-like photosystem II reaction center PsbP family protein	Mog1/PsbP/DUF1795-like photosystem II reaction center PsbP family protein	plastid
AT3G56910.1	PSRP5	plastid-specific 50S ribosomal protein 5	plastid
AT3G56940.1	CRD1, CHL27, ACSF	dicarboxylate diiron protein, putative (Crd1)	plastid
AT3G57150.1	NAP57, AtNAP57, CBF5, AtCBF5	homologue of NAP57	nucleus
AT3G57290.1	EIF3E, TIF3E1, ATEIF3E-1, INT-6, ATINT6, INT6	eukaryotic translation initiation factor 3E	cytosol
AT3G57410.1	VLN3, ATVLN3	villin 3	cytosol
AT3G58010.1	PGL34	plastoglobulin 34kD	plastid
AT3G58610.3	ketol-acid reductoisomerase	ketol-acid reductoisomerase	plastid
AT3G58730.1	vacuolar ATP synthase subunit D (VATD) / V-ATPase D subunit / vacuolar proton pump D subunit (VATPD)	vacuolar ATP synthase subunit D (VATD) / V-ATPase D subunit / vacuolar proton pump D subunit (VATPD)	vacuole,golgi
AT3G59020.1	ARM repeat superfamily protein	ARM repeat superfamily protein	nucleus,cytosol
AT3G59780.1	Rhodanese/Cell cycle control phosphatase superfamily protein	Rhodanese/Cell cycle control phosphatase superfamily protein	plastid
AT3G59820.1	LETM1-like protein	LETM1-like protein	mitochondrion
AT3G59970.3	MTHFR1	methylenetetrahydrofolate reductase 1	cytosol
AT3G60190.1	ADL4, ADLP2, EDR3, DRP1E, ADL1E, DL1E	DYNAMIN-like 1E	cytosol

AT3G60245.1	Zinc-binding ribosomal protein family protein	Zinc-binding ribosomal protein family protein	cytosol
AT3G60600.1	VAP27-1, VAP, (AT)VAP, VAP27	vesicle associated protein	plasma membrane,endoplasmic reticulum
AT3G60750.2	Transketolase	Transketolase	plastid
AT3G61050.2	NTMC2TYPE4, NTMC2T4	Calcium-dependent lipid-binding (CaLB domain) family protein	endoplasmic reticulum
AT3G61260.1	Remorin family protein	Remorin family protein	plasma membrane
AT3G61430.1	PIP1A, ATPIP1, PIP1, PIP1;1	plasma membrane intrinsic protein 1A	plasma membrane
AT3G61440.1	ATCYSC1, ARATH;BSAS3;1, CYSC1	cysteine synthase C1	mitochondrion
AT3G61470.1	LHCA2	photosystem I light harvesting complex gene 2	plastid
AT3G61820.1	Eukaryotic aspartyl protease family protein	Eukaryotic aspartyl protease family protein	extracellular
AT3G61870.1	unknown protein;FUNCTIONS IN: molecular_function unknown;INVOLVED IN: biological_process unknown;LOCATED IN: chloroplast, chloroplast inner membrane, chloroplast envelope;EXPRESSED IN: 23 plant structures;EXPRESSED DURING: 14	unknown protein;FUNCTIONS IN: molecular_function unknown;INVOLVED IN: biological_process unknown;LOCATED IN: chloroplast, chloroplast inner membrane, chloroplast envelope;EXPRESSED IN: 23 plant structures;EXPRESSED DURING: 14	plastid
AT3G62030.3	ROC4	rotamase CYP 4	plastid
AT3G62360.1	Carbohydrate-binding-like fold	Carbohydrate-binding-like fold	endoplasmic reticulum
AT3G62870.1	Ribosomal protein L7Ae/L30e/S12e/Gadd45 family protein	Ribosomal protein L7Ae/L30e/S12e/Gadd45 family protein	cytosol
AT3G63080.1	ATGPX5, MEE42, GPX5	glutathione peroxidase 5	plasma membrane
AT3G63130.2	RANGAP1	RAN GTPase activating protein 1	nucleus
AT3G63140.1	CSP41A	chloroplast stem-loop binding protein of 41 kDa	plastid
AT3G63160.1	FUNCTIONS IN: molecular_function unknown;INVOLVED IN: biological_process unknown;LOCATED IN: chloroplast outer membrane, thylakoid, chloroplast thylakoid membrane, chloroplast, chloroplast envelope;EXPRESSED IN: 21 plant struct	FUNCTIONS IN: molecular_function unknown;INVOLVED IN: biological_process unknown;LOCATED IN: chloroplast outer membrane, thylakoid, chloroplast thylakoid membrane, chloroplast, chloroplast envelope;EXPRESSED IN: 21 plant struct	plastid
AT3G63190.1	RRF, HFP108, cpRRF, AtcpRRF	ribosome recycling factor, chloroplast precursor	plastid

AT3G63410.1	APG1, VTE3, IEP37, E37	S-adenosyl-L-methionine-dependent methyltransferases superfamily protein	plastid
AT3G63460.3	transducin family protein / WD-40 repeat family protein	transducin family protein / WD-40 repeat family protein	nucleus
AT3G63490.1	Ribosomal protein L1p/L10e family	Ribosomal protein L1p/L10e family	plastid
AT3G63520.1	CCD1, ATCCD1, ATNCED1, NCED1	carotenoid cleavage dioxygenase 1	vacuole
AT4G00100.1	ATRPS13A, RPS13, PFL2, RPS13A	ribosomal protein S13A	cytosol
AT4G00570.1	NAD-ME2	NAD-dependent malic enzyme 2	mitochondrion
AT4G00830.4	RNA-binding (RRM/RBD/RNP motifs) family protein	RNA-binding (RRM/RBD/RNP motifs) family protein	nucleus
AT4G00860.1	ATOZI1, AT0ZI1	Protein of unknown function (DUF1138)	mitochondrion
AT4G01050.1	TROL	thylakoid rhodanese-like	plastid
AT4G01100.1	ADNT1	adenine nucleotide transporter 1	mitochondrion
AT4G01150.1	unknown protein;FUNCTIONS IN: molecular_function unknown;INVOLVED IN: biological_process unknown;LOCATED IN: thylakoid, chloroplast thylakoid membrane, chloroplast, plastoglobule, chloroplast envelope;EXPRESSED IN: 23 plant st	unknown protein;FUNCTIONS IN: molecular_function unknown;INVOLVED IN: biological_process unknown;LOCATED IN: thylakoid, chloroplast thylakoid membrane, chloroplast, plastoglobule, chloroplast envelope;EXPRESSED IN: 23 plant st	plastid
AT4G01310.1	Ribosomal L5P family protein	Ribosomal L5P family protein	plastid
AT4G01690.1	PPOX, HEMG1, PPO1	Flavin containing amine oxidoreductase family	plastid
AT4G01800.1	AGY1, AtcpSecA, SECA1	Albino or Glassy Yellow 1	plastid
AT4G02230.1	Ribosomal protein L19e family protein	Ribosomal protein L19e family protein	cytosol
AT4G02450.2	HSP20-like chaperones superfamily protein	HSP20-like chaperones superfamily protein	cytosol
AT4G02510.1	TOC159, TOC86, PPI2, TOC160, ATTOC159	translocon at the outer envelope membrane of chloroplasts 159	plastid
AT4G02520.1	ATGSTF2, ATPM24.1, ATPM24, GST2, GSTF2	glutathione S-transferase PHI 2	cytosol
AT4G02530.1	chloroplast thylakoid lumen protein	chloroplast thylakoid lumen protein	plastid
AT4G02580.1	NADH-ubiquinone oxidoreductase 24 kDa subunit, putative	NADH-ubiquinone oxidoreductase 24 kDa subunit, putative	mitochondrion
AT4G02620.1	vacuolar ATPase subunit F family protein	vacuolar ATPase subunit F family protein	vacuole
AT4G02725.1	unknown protein;FUNCTIONS IN: molecular_function unknown;INVOLVED IN:	unknown protein;FUNCTIONS IN: molecular_function unknown;INVOLVED IN:	plastid

	biological_process unknown;LOCATED IN: chloroplast, membrane;Has 35333 Blast hits to 34131 proteins in 2444 species: Archae - 798;Bacteria - 22429;Metazoa	biological_process unknown;LOCATED IN: chloroplast, membrane;Has 35333 Blast hits to 34131 proteins in 2444 species: Archae - 798;Bacteria - 22429;Metazoa	
AT4G02770.1	PSAD-1	photosystem I subunit D-1	plastid
AT4G02930.1	GTP binding Elongation factor Tu family protein	GTP binding Elongation factor Tu family protein	mitochondrion
AT4G03280.2	PETC, PGR1	photosynthetic electron transfer C	plastid
AT4G03520.1	ATHM2	Thioredoxin superfamily protein	plastid
AT4G04020.1	FIB	fibrillin	plastid
AT4G04210.1	PUX4	plant UBX domain containing protein 4	nucleus
AT4G04640.1	ATPC1	ATPase, F1 complex, gamma subunit protein	plastid
AT4G05180.1	PSBQ, PSBQ-2, PSII-Q	photosystem II subunit Q-2	plastid
AT4G08520.1	SNARE-like superfamily protein	SNARE-like superfamily protein	cytosol
AT4G08870.1	Arginase/deacetylase superfamily protein	Arginase/deacetylase superfamily protein	mitochondrion
AT4G09000.1	GRF1, GF14 CHI	general regulatory factor 1	cytosol
AT4G09010.1	APX4, TL29	ascorbate peroxidase 4	plastid
AT4G09040.2	RNA-binding (RRM/RBD/RNP motifs) family protein	RNA-binding (RRM/RBD/RNP motifs) family protein	plastid
AT4G09320.1	NDPK1	Nucleoside diphosphate kinase family protein	cytosol
AT4G09650.1	ATPD	ATP synthase delta-subunit gene	plastid
AT4G10000.2	Thioredoxin family protein	Thioredoxin family protein	plastid
AT4G10340.1	LHCB5	light harvesting complex of photosystem II 5	plastid
AT4G10480.1	Nascent polypeptide-associated complex (NAC), alpha subunit family protein	Nascent polypeptide-associated complex (NAC), alpha subunit family protein	cytosol
AT4G10750.1	Phosphoenolpyruvate carboxylase family protein	Phosphoenolpyruvate carboxylase family protein	plastid
AT4G11010.1	NDPK3	nucleoside diphosphate kinase 3	mitochondrion
AT4G11150.1	TUF, emb2448, TUFF, VHA-E1	vacuolar ATP synthase subunit E1	vacuole,golgi
AT4G11420.1	EIF3A, ATEIF3A-1, EIF3A-1, ATTIF3A1, TIF3A1	eukaryotic translation initiation factor 3A	nucleus,cytosol
AT4G11960.1	PGRL1B	PGR5-like B	plastid
AT4G12060.1	Double Clp-N motif protein	Double Clp-N motif protein	plastid
AT4G12340.1	copper ion binding	copper ion binding	mitochondrion
AT4G12420.2	SKU5	Cupredoxin superfamily protein	plasma membrane,extracellular
AT4G12650.1	Endomembrane protein 70 protein family	Endomembrane protein 70 protein family	golgi
AT4G12730.1	FLA2	FASCICLIN-like arabinogalactan 2	plasma membrane

AT4G12800.1	PSAL	photosystem I subunit l	plastid
AT4G13010.1	Oxidoreductase, zinc-binding dehydrogenase family protein	Oxidoreductase, zinc-binding dehydrogenase family protein	cytosol
AT4G13200.1	unknown protein;FUNCTIONS IN: molecular_function unknown;INVOLVED IN: biological_process unknown;LOCATED IN: thylakoid, chloroplast thylakoid membrane, chloroplast, plastoglobule;EXPRESSED IN: 22 plant structures;EXPRESSED DU	unknown protein;FUNCTIONS IN: molecular_function unknown;INVOLVED IN: biological_process unknown;LOCATED IN: thylakoid, chloroplast thylakoid membrane, chloroplast, plastoglobule;EXPRESSED IN: 22 plant structures;EXPRESSED DU	plastid
AT4G13220.1	unknown protein;FUNCTIONS IN: molecular_function unknown;INVOLVED IN: biological_process unknown;LOCATED IN: chloroplast;EXPRESSED IN: 22 plant structures;EXPRESSED DURING: 13 growth stages;Has 27 Blast hits to 27 proteins i	unknown protein;FUNCTIONS IN: molecular_function unknown;INVOLVED IN: biological_process unknown;LOCATED IN: chloroplast;EXPRESSED IN: 22 plant structures;EXPRESSED DURING: 13 growth stages;Has 27 Blast hits to 27 proteins i	plastid
AT4G13670.1	PTAC5	plastid transcriptionally active 5	plastid
AT4G13930.1	SHM4	serine hydroxymethyltransferase 4	cytosol
AT4G13940.1	HOG1, EMB1395, SAHH1, MEE58, ATSAHH1	S-adenosyl-L-homocysteine hydrolase	cytosol
AT4G14070.1	AAE15	acyl-activating enzyme 15	plastid
AT4G14210.2	PDS3, PDS, PDE226	phytoene desaturase 3	plastid
AT4G14870.1	SECE1	secE/sec61-gamma protein transport protein	plastid
AT4G14880.4	OASA1	O-acetylserine (thiol) lyase (OAS-TL) isoform A1	cytosol
AT4G15000.2	Ribosomal L27e protein family	Ribosomal L27e protein family	cytosol
AT4G15110.1	CYP97B3	cytochrome P450, family 97, subfamily B, polypeptide 3	plastid
AT4G15410.1	PUX5	serine/threonine protein phosphatase 2A 55 kDa regulatory subunit B prime gamma	nucleus
AT4G15510.3	Photosystem II reaction center PsbP family protein	Photosystem II reaction center PsbP family protein	plastid
AT4G15545.1	unknown protein;BEST Arabidopsis thaliana protein match is: unknown protein (TAIR:AT1G16520.1);Has 30201 Blast hits to 17322 proteins in 780 species: Archae - 12;Bacteria -	unknown protein;BEST Arabidopsis thaliana protein match is: unknown protein (TAIR:AT1G16520.1);Has 30201 Blast hits to 17322 proteins in 780 species: Archae - 12;Bacteria - 1396;Metazoa - 17338;Fungi - 3422;Plants - 5037;Vi	nucleus

	1396;Metazoa - 17338;Fungi - 3422;Plants - 5037;Vi		
AT4G16143.2	IMPA-2	importin alpha isoform 2	cytosol
AT4G16155.1	dihydrolipoyl dehydrogenases	dihydrolipoyl dehydrogenases	plastid
AT4G16450.2	unknown protein;FUNCTIONS IN: molecular_function unknown;INVOLVED IN: photorespiration;LOCATED IN: mitochondrion, mitochondrial membrane, mitochondrial respiratory chain complex I, respiratory chain complex I;EXPRESSED IN: 25	unknown protein;FUNCTIONS IN: molecular_function unknown;INVOLVED IN: photorespiration;LOCATED IN: mitochondrion, mitochondrial membrane, mitochondrial respiratory chain complex I, respiratory chain complex I;EXPRESSED IN: 25	vacuole,golgi
AT4G16500.1	Cystatin/monellin superfamily protein	Cystatin/monellin superfamily protein	extracellular
AT4G16660.1	heat shock protein 70 (Hsp 70) family protein	heat shock protein 70 (Hsp 70) family protein	endoplasmic reticulum
AT4G16720.1	Ribosomal protein L23/L15e family protein	Ribosomal protein L23/L15e family protein	cytosol
AT4G17090.1	CT-BMY, BAM3, BMY8	chloroplast beta-amylase	plastid
AT4G17140.1	pleckstrin homology (PH) domain-containing protein	pleckstrin homology (PH) domain-containing protein	golgi
AT4G17170.1	AT-RAB2, ATRABB1C, ATRAB2A, RAB2A, RABB1C, ATRAB-B1B, RAB-B1B	RAB GTPase homolog B1C	vacuole
AT4G17340.1	TIP2;2, DELTA-TIP2	tonoplast intrinsic protein 2;2	vacuole
AT4G17530.1	RAB1C, ATRAB1C, ATRABD2C	RAB GTPase homolog 1C	cytosol
AT4G17600.1	LIL3	Chlorophyll A-B binding family protein	plastid
AT4G18100.1	Ribosomal protein L32e	Ribosomal protein L32e	cytosol
AT4G18370.1	DEG5, DEGP5, HHOA	DEGP protease 5	plastid
AT4G18480.1	CHLI1, CH42, CH-42, CHL11, CHLI-1	P-loop containing nucleoside triphosphate hydrolases superfamily protein	plastid
AT4G18810.1	NAD(P)-binding Rossmann-fold superfamily protein	NAD(P)-binding Rossmann-fold superfamily protein	plastid
AT4G19170.1	NCED4, CCD4	nine-cis-epoxycarotenoid dioxygenase 4	plastid
AT4G20130.1	PTAC14	plastid transcriptionally active 14	plastid
AT4G20260.3	ATPCAP1, PCAP1	plasma-membrane associated cation-binding protein 1	cytosol
AT4G20360.1	ATRAB8D, ATRABE1B, RABE1b	RAB GTPase homolog E1B	plastid
AT4G20890.1	TUB9	tubulin beta-9 chain	cytosol
AT4G20980.4	Eukaryotic translation initiation factor 3 subunit 7 (eIF-3)	Eukaryotic translation initiation factor 3 subunit 7 (eIF-3)	cytosol
AT4G21105.1	cytochrome-c oxidases;electron carriers	cytochrome-c oxidases;electron carriers	mitochondrion
AT4G21150.1	HAP6	ribophorin II (RPN2) family protein	endoplasmic reticulum

AT4G21280.1	PSBQ, PSBQA, PSBQ-1	photosystem II subunit QA	plastid
AT4G21660.2	proline-rich spliceosome-associated (PSP) family protein	proline-rich spliceosome-associated (PSP) family protein	nucleus
AT4G21960.1	PRXR1	Peroxidase superfamily protein	extracellular
AT4G22010.1	sks4	SKU5 similar 4	extracellular
AT4G22240.1	Plastid-lipid associated protein PAP / fibrillin family protein	Plastid-lipid associated protein PAP / fibrillin family protein	plastid
AT4G22670.1	AtHip1, HIP1, TPR11	HSP70-interacting protein 1	cytosol
AT4G22690.1	CYP706A1	cytochrome P450, family 706, subfamily A, polypeptide 1	endoplasmic reticulum
AT4G22890.3	PGR5-LIKE A	PGR5-LIKE A	plastid
AT4G23180.1	CRK10, RLK4	cysteine-rich RLK (RECEPTOR-like protein kinase) 10	plasma membrane
AT4G23400.1	PIP1D, PIP1;5	plasma membrane intrinsic protein 1;5	plasma membrane
AT4G23630.1	BTI1, RTNLB1	VIRB2-interacting protein 1	plasma membrane
AT4G23650.1	CDPK6, CPK3	calcium-dependent protein kinase 6	cytosol
AT4G23670.1	Polyketide cyclase/dehydrase and lipid transport superfamily protein	Polyketide cyclase/dehydrase and lipid transport superfamily protein	cytosol
AT4G23850.1	LACS4	AMP-dependent synthetase and ligase family protein	plasma membrane
AT4G23890.1	unknown protein;FUNCTIONS IN: molecular_function unknown;INVOLVED IN: biological_process unknown;LOCATED IN: chloroplast thylakoid membrane, chloroplast;EXPRESSED IN: 22 plant structures;EXPRESSED DURING: 13 growth stages;CO	unknown protein;FUNCTIONS IN: molecular_function unknown;INVOLVED IN: biological_process unknown;LOCATED IN: chloroplast thylakoid membrane, chloroplast;EXPRESSED IN: 22 plant structures;EXPRESSED DURING: 13 growth stages;CO	plastid
AT4G24190.2	SHD, AtHsp90.7, AtHsp90-7	Chaperone protein htpG family protein	endoplasmic reticulum
AT4G24280.1	cpHsc70-1	chloroplast heat shock protein 70-1	plastid
AT4G24750.1	Rhodanese/Cell cycle control phosphatase superfamily protein	Rhodanese/Cell cycle control phosphatase superfamily protein	plastid
AT4G24770.1	RBP31, ATRBP31, CP31, ATRBP33	31-kDa RNA binding protein	plastid
AT4G24800.3	MA3 domain-containing protein	MA3 domain-containing protein	cytosol
AT4G24820.2	26S proteasome, regulatory subunit Rpn7;Proteasome component (PCI) domain	26S proteasome, regulatory subunit Rpn7;Proteasome component (PCI) domain	cytosol
AT4G25080.4	CHLM	magnesium-protoporphyrin IX methyltransferase	cytosol

AT4G25130.1	PMSR4	peptide met sulfoxide reductase 4	plastid
AT4G25210.1	DNA-binding storekeeper protein-related transcriptional regulator	DNA-binding storekeeper protein-related transcriptional regulator	nucleus
AT4G25450.3	ATNAP8, NAP8	non-intrinsic ABC protein 8	plastid
AT4G25550.1	Cleavage/polyadenylation specificity factor, 25kDa subunit	Cleavage/polyadenylation specificity factor, 25kDa subunit	cytosol
AT5G52470.1	FIB1, FBR1, ATFIB1, ATFBR1, SKIP7	fibrillarin 1	nucleus
AT4G25650.1	ACD1-LIKE, PTC52, TIC55-IV	ACD1-like	plastid
AT4G25740.1	RNA binding Plectin/S10 domain-containing protein	RNA binding Plectin/S10 domain-containing protein	cytosol
AT4G25970.1	PSD3	phosphatidylserine decarboxylase 3	endoplasmic reticulum
AT4G26110.2	NAP1;1	nucleosome assembly protein1;1	nucleus
AT4G26530.1	Aldolase superfamily protein	Aldolase superfamily protein	plastid
AT4G26630.2	DEK domain-containing chromatin associated protein	DEK domain-containing chromatin associated protein	nucleus
AT4G26780.1	AR192	Co-chaperone GrpE family protein	mitochondrion
AT4G26910.3	Dihydrolipoamide succinyltransferase	Dihydrolipoamide succinyltransferase	mitochondrion
AT4G26970.1	ACO2	aconitase 2	mitochondrion
AT4G27090.1	Ribosomal protein L14	Ribosomal protein L14	cytosol
AT4G27440.2	PORB	protochlorophyllide oxidoreductase B	plastid
AT4G27500.1	PPI1	proton pump interactor 1	plasma membrane,endoplasmic reticulum
AT4G27520.1	ENODL2, AtENODL2	early nodulin-like protein 2	plasma membrane
AT4G27585.1	SPFH/Band 7/PHB domain-containing membrane-associated protein family	SPFH/Band 7/PHB domain-containing membrane-associated protein family	mitochondrion
AT4G27700.1	Rhodanese/Cell cycle control phosphatase superfamily protein	Rhodanese/Cell cycle control phosphatase superfamily protein	plastid
AT4G28220.1	NDB1	NAD(P)H dehydrogenase B1	mitochondrion
AT4G28510.1	ATPHB1, PHB1	prohibitin 1	mitochondrion
AT4G28660.1	PSB28	photosystem II reaction center PSB28 protein	plastid
AT4G28750.1	PSAE-1	Photosystem I reaction centre subunit IV / PsaE protein	plastid
AT4G29010.1	AIM1	Enoyl-CoA hydratase/isomerase family	peroxisome
AT4G29060.1	emb2726	elongation factor Ts family protein	plastid
AT4G29130.1	ATHXK1, GIN2, HXK1	hexokinase 1	mitochondrion
AT4G29410.2	Ribosomal L28e protein family	Ribosomal L28e protein family	cytosol
AT4G29480.1	Mitochondrial ATP synthase subunit G protein	Mitochondrial ATP synthase subunit G protein	mitochondrion

AT4G29520.1	LOCATED IN: endoplasmic reticulum, plasma membrane;EXPRESSED IN: 23 plant structures;EXPRESSED DURING: 14 growth stages;CONTAINS InterPro DOMAIN/s: Saposin B (InterPro:IPR008139);Has 137 Blast hits to 137 proteins in 50 specie	LOCATED IN: endoplasmic reticulum, plasma membrane;EXPRESSED IN: 23 plant structures;EXPRESSED DURING: 14 growth stages;CONTAINS InterPro DOMAIN/s: Saposin B (InterPro:IPR008139);Has 137 Blast hits to 137 proteins in 50 specie	endoplasmic reticulum
AT4G30010.1	unknown protein;FUNCTIONS IN: molecular_function unknown;INVOLVED IN: biological_process unknown;LOCATED IN: mitochondrion, plastid;EXPRESSED IN: 26 plant structures;EXPRESSED DURING: 15 growth stages;Has 39 Blast hits to 39	unknown protein;FUNCTIONS IN: molecular_function unknown;INVOLVED IN: biological_process unknown;LOCATED IN: mitochondrion, plastid;EXPRESSED IN: 26 plant structures;EXPRESSED DURING: 15 growth stages;Has 39 Blast hits to 39	mitochondrion
AT4G30190.1	AHA2, PMA2, HA2	H(+)-ATPase 2	plasma membrane
AT4G30480.2	TPR1, AtTPR1	Tetratricopeptide repeat (TPR)-like superfamily protein	nucleus
AT4G31340.2	myosin heavy chain-related	myosin heavy chain-related	extracellular
AT4G31390.1	Protein kinase superfamily protein	Protein kinase superfamily protein	plastid
AT4G31480.2	Coatomer, beta subunit	Coatomer, beta subunit	cytosol
AT4G31530.1	NAD(P)-binding Rossmann-fold superfamily protein	NAD(P)-binding Rossmann-fold superfamily protein	plastid
AT4G31700.1	RPS6, RPS6A	ribosomal protein S6	cytosol
AT4G31880.2	LOCATED IN: cytosol;EXPRESSED IN: 24 plant structures;EXPRESSED DURING: 14 growth stages;BEST Arabidopsis thaliana protein match is: Tudor/PWWP/MBT superfamily protein (TAIR:AT1G15940.1).	LOCATED IN: cytosol;EXPRESSED IN: 24 plant structures;EXPRESSED DURING: 14 growth stages;BEST Arabidopsis thaliana protein match is: Tudor/PWWP/MBT superfamily protein (TAIR:AT1G15940.1).	nucleus
AT4G32260.1	ATPase, F0 complex, subunit B/B, bacterial/chloroplast	ATPase, F0 complex, subunit B/B, bacterial/chloroplast	plastid
AT4G32330.2	TPX2 (targeting protein for Xklp2) protein family	TPX2 (targeting protein for Xklp2) protein family	nucleus
AT4G32470.1	Cytochrome bd ubiquinol oxidase, 14kDa subunit	Cytochrome bd ubiquinol oxidase, 14kDa subunit	mitochondrion
AT4G32770.1	VTE1, ATSDX1	tocopherol cyclase, chloroplast / vitamin E deficient 1 (VTE1) / sucrose export defective 1 (SXD1)	plastid
AT4G33010.1	AtGLDP1, GLDP1	glycine decarboxylase P-protein 1	mitochondrion
AT4G33250.1	EIF3K, TIF3K1, ATTIF3K1	eukaryotic translation initiation factor 3K	cytosol

AT4G33350.1	Tic22-like family protein	Tic22-like family protein	plastid
AT4G33500.1	Protein phosphatase 2C family protein	Protein phosphatase 2C family protein	plastid
AT4G33625.1	FUNCTIONS IN: molecular_function unknown;INVOLVED IN: biological_process unknown;LOCATED IN: plasma membrane, vacuole;EXPRESSED IN: cultured cell;CONTAINS InterPro DOMAIN/s: Golgi apparatus membrane protein TVP15 (InterPro:IPR	FUNCTIONS IN: molecular_function unknown;INVOLVED IN: biological_process unknown;LOCATED IN: plasma membrane, vacuole;EXPRESSED IN: cultured cell;CONTAINS InterPro DOMAIN/s: Golgi apparatus membrane protein TVP15 (InterPro:IPR	vacuole
AT4G33650.1	ADL2, DRP3A	dynamin-related protein 3A	mitochondrion
AT4G34110.1	PAB2, PABP2, ATPAB2	poly(A) binding protein 2	nucleus
AT4G34240.1	ALDH3I1, ALDH3	aldehyde dehydrogenase 3I1	plastid
AT4G34450.1	coatomer gamma-2 subunit, putative / gamma-2 coat protein, putative / gamma-2 COP, putative	coatomer gamma-2 subunit, putative / gamma-2 coat protein, putative / gamma-2 COP, putative	cytosol
AT4G34620.1	SSR16	small subunit ribosomal protein 16	plastid
AT4G34670.1	Ribosomal protein S3Ae	Ribosomal protein S3Ae	cytosol
AT4G34700.1	CIB22, AtCIB22	LYR family of Fe/S cluster biogenesis protein	mitochondrion
AT4G35000.1	APX3	ascorbate peroxidase 3	peroxisome
AT4G35090.1	CAT2	catalase 2	peroxisome
AT4G35100.2	PIP3	plasma membrane intrinsic protein 3	plasma membrane
AT4G35250.1	NAD(P)-binding Rossmann-fold superfamily protein	NAD(P)-binding Rossmann-fold superfamily protein	plastid
AT4G35260.1	IDH1, IDH-I	isocitrate dehydrogenase 1	mitochondrion
AT4G35450.3	AKR2, AFT, AKR2A	ankyrin repeat-containing protein 2	nucleus
AT4G35760.1	NAD(P)H dehydrogenase (quinone)s	NAD(P)H dehydrogenase (quinone)s	plastid
AT4G35860.1	ATRABB1B, ATGB2, ATRAB2C, GB2	GTP-binding 2	golgi
AT4G36250.1	ALDH3F1	aldehyde dehydrogenase 3F1	cytosol
AT4G36530.1	alpha/beta-Hydrolases superfamily protein	alpha/beta-Hydrolases superfamily protein	plastid
AT4G37200.1	HCF164	Thioredoxin superfamily protein	plastid
AT4G37910.1	mtHsc70-1	mitochondrial heat shock protein 70-1	mitochondrion
AT4G37925.1	NDH-M	subunit NDH-M of NAD(P)H:plastoquinone dehydrogenase complex	plastid
AT4G37930.1	SHM1, STM, SHMT1	serine transhydroxymethyltransferase 1	mitochondrion

AT4G38220.1	Peptidase M20/M25/M40 family protein	Peptidase M20/M25/M40 family protein	endoplasmic reticulum
AT4G38350.1	Patched family protein	Patched family protein	vacuole
AT4G38600.1	KAK, UPL3	HEAT repeat;HECT-domain (ubiquitin-transferase)	golgi
AT4G38630.1	RPN10, MCB1, ATMCB1, MBP1	regulatory particle non-ATPase 10	cytosol
AT4G38970.1	FBA2	fructose-bisphosphate aldolase 2	plastid
AT4G39080.1	VHA-A3	vacuolar proton ATPase A3	vacuole
AT4G39200.2	Ribosomal protein S25 family protein	Ribosomal protein S25 family protein	nucleus
AT4G39260.1	CCR1, ATGRP8, GR-RBP8, GRP8	cold, circadian rhythm, and RNA binding 1	nucleus
AT4G39460.1	SAMC1, SAMT1	S-adenosylmethionine carrier 1	plastid
AT4G39520.1	GTP-binding protein-related	GTP-binding protein-related	cytosol
AT4G39680.2	SAP domain-containing protein	SAP domain-containing protein	nucleus
AT4G39690.1	FUNCTIONS IN: molecular_function unknown;INVOLVED IN: biological_process unknown;LOCATED IN: mitochondrion;EXPRESSED IN: 26 plant structures;EXPRESSED DURING: 14 growth stages;CONTAINS InterPro DOMAIN/s: Mitochondrial inner m	FUNCTIONS IN: molecular_function unknown;INVOLVED IN: biological_process unknown;LOCATED IN: mitochondrion;EXPRESSED IN: 26 plant structures;EXPRESSED DURING: 14 growth stages;CONTAINS InterPro DOMAIN/s: Mitochondrial inner m	mitochondrion
AT4G39710.1	FKBP16-2	FK506-binding protein 16-2	plastid
AT4G39730.1	Lipase/lipooxygenase, PLAT/LH2 family protein	Lipase/lipooxygenase, PLAT/LH2 family protein	plastid
AT4G39960.1	Molecular chaperone Hsp40/DnaJ family protein	Molecular chaperone Hsp40/DnaJ family protein	plastid
AT5G01220.1	SQD2	sulfoquinovosyldiacylglycerol 2	plastid
AT5G01530.1	LHCB4.1	light harvesting complex photosystem II	plastid
AT5G01590.1	unknown protein;FUNCTIONS IN: molecular_function unknown;INVOLVED IN: biological_process unknown;LOCATED IN: chloroplast, chloroplast envelope;EXPRESSED IN: 22 plant structures;EXPRESSED DURING: 13 growth stages;Has 60 Blast	unknown protein;FUNCTIONS IN: molecular_function unknown;INVOLVED IN: biological_process unknown;LOCATED IN: chloroplast, chloroplast envelope;EXPRESSED IN: 22 plant structures;EXPRESSED DURING: 13 growth stages;Has 60 Blast	plastid
AT5G01750.2	Protein of unknown function (DUF567)	Protein of unknown function (DUF567)	golgi,cytosol
AT5G01920.1	STN8	Protein kinase superfamily protein	plastid

AT5G02050.1	Mitochondrial glycoprotein family protein	Mitochondrial glycoprotein family protein	mitochondrion
AT5G02120.1	OHP	one helix protein	plastid
AT5G02160.1	unknown protein;FUNCTIONS IN: molecular_function unknown;INVOLVED IN: biological_process unknown;LOCATED IN: chloroplast thylakoid membrane;EXPRESSED IN: 23 plant structures;EXPRESSED DURING: 13 growth stages;Has 121 Blast h	unknown protein;FUNCTIONS IN: molecular_function unknown;INVOLVED IN: biological_process unknown;LOCATED IN: chloroplast thylakoid membrane;EXPRESSED IN: 23 plant structures;EXPRESSED DURING: 13 growth stages;Has 121 Blast h	plastid
AT5G02450.1	Ribosomal protein L36e family protein	Ribosomal protein L36e family protein	cytosol
AT5G02500.1	HSC70-1, HSP70-1, AT-HSC70-1, HSC70	heat shock cognate protein 70-1	cytosol
AT5G02610.1	Ribosomal L29 family protein	Ribosomal L29 family protein	cytosol
AT5G02870.1	Ribosomal protein L4/L1 family	Ribosomal protein L4/L1 family	cytosol
AT5G02940.1	Protein of unknown function (DUF1012)	Protein of unknown function (DUF1012)	plastid
AT5G03290.1	IDH-V	isocitrate dehydrogenase V	mitochondrion
AT5G03740.1	HD2C, HDT3	histone deacetylase 2C	nucleus
AT5G03880.1	Thioredoxin family protein	Thioredoxin family protein	plastid
AT5G03900.2	Iron-sulphur cluster biosynthesis family protein	Iron-sulphur cluster biosynthesis family protein	plastid
AT5G03940.1	FFC, 54CP, CPSRP54, SRP54CP	chloroplast signal recognition particle 54 kDa subunit	plastid
AT5G04130.1	GYRB2	DNA GYRASE B2	mitochondrion
AT5G04140.1	GLU1, GLS1, GLUS, FD-GOGAT	glutamate synthase 1	plastid
AT5G04280.1	RNA-binding (RRM/RBD/RNP motifs) family protein with retrovirus zinc finger-like domain	RNA-binding (RRM/RBD/RNP motifs) family protein with retrovirus zinc finger-like domain	nucleus
AT5G04430.1	BTR1, BTR1S	binding to TOMV RNA 1L (long form)	nucleus
AT5G04900.1	NOL	NYC1-like	plastid
AT5G04990.1	SUN1, ATSUN1	SAD1/UNC-84 domain protein 1	endoplasmic reticulum,nucleus
AT5G05000.3	TOC34, ATTOC34, OEP34	translocon at the outer envelope membrane of chloroplasts 34	cytosol
AT5G05010.2	clathrin adaptor complexes medium subunit family protein	clathrin adaptor complexes medium subunit family protein	cytosol
AT5G05520.1	Outer membrane OMP85 family protein	Outer membrane OMP85 family protein	mitochondrion
AT5G05740.3	EGY2	ethylene-dependent gravitropism-deficient and yellow-green-like 2	plastid
AT5G06290.1	2-Cys Prx B, 2CPB	2-cysteine peroxiredoxin B	plastid

AT5G07020.1	proline-rich family protein	proline-rich family protein	plastid
AT5G07340.1	Calreticulin family protein	Calreticulin family protein	endoplasmic reticulum
AT5G07350.1	Tudor1, AtTudor1, TSN1	TUDOR-SN protein 1	nucleus
AT5G08050.1	Protein of unknown function (DUF1118)	Protein of unknown function (DUF1118)	plastid
AT5G08060.1	unknown protein;FUNCTIONS IN: molecular_function unknown;INVOLVED IN: biological_process unknown;LOCATED IN: mitochondrion;EXPRESSED IN: 24 plant structures;EXPRESSED DURING: 16 growth stages;Has 42 Blast hits to 42 proteins	unknown protein;FUNCTIONS IN: molecular_function unknown;INVOLVED IN: biological_process unknown;LOCATED IN: mitochondrion;EXPRESSED IN: 24 plant structures;EXPRESSED DURING: 16 growth stages;Has 42 Blast hits to 42 proteins	mitochondrion
AT5G08080.3	SYP132	syntaxin of plants 132	cytosol
AT5G08280.1	HEMC	hydroxymethylbilane synthase	plastid
AT5G08530.1	CI51	51 kDa subunit of complex I	mitochondrion
AT5G08540.1	unknown protein;FUNCTIONS IN: molecular_function unknown;INVOLVED IN: biological_process unknown;LOCATED IN: chloroplast thylakoid membrane, chloroplast, chloroplast envelope;EXPRESSED IN: 24 plant structures;EXPRESSED DURING	unknown protein;FUNCTIONS IN: molecular_function unknown;INVOLVED IN: biological_process unknown;LOCATED IN: chloroplast thylakoid membrane, chloroplast, chloroplast envelope;EXPRESSED IN: 24 plant structures;EXPRESSED DURING	plastid
AT5G08590.1	ASK2, SNRK2-1, SNRK2.1, SRK2G	SNF1-related protein kinase 2.1	nucleus
AT5G08690.1	ATP synthase alpha/beta family protein	ATP synthase alpha/beta family protein	mitochondrion
AT5G08740.1	NDC1	NAD(P)H dehydrogenase C1	mitochondrion,plastid
AT5G09510.1	Ribosomal protein S19 family protein	Ribosomal protein S19 family protein	cytosol
AT5G09590.1	MTHSC70-2, HSC70-5	mitochondrial HSO70 2	mitochondrion
AT5G09660.2	PMDH2	peroxisomal NAD-malate dehydrogenase 2	peroxisome
AT5G09810.1	ACT7	actin 7	cytosol
AT5G10010.1	unknown protein;FUNCTIONS IN: molecular_function unknown;INVOLVED IN: biological_process unknown;LOCATED IN: nucleolus;EXPRESSED IN: 25 plant structures;EXPRESSED DURING: 15 growth	unknown protein;FUNCTIONS IN: molecular_function unknown;INVOLVED IN: biological_process unknown;LOCATED IN: nucleolus;EXPRESSED IN: 25 plant structures;EXPRESSED DURING: 15 growth stages;BEST Arabidopsis thaliana protein ma	nucleus

	stages;BEST Arabidopsis thaliana protein ma		
AT5G10360.1	EMB3010, RPS6B	Ribosomal protein S6e	cytosol
AT5G10450.1	GRF6, AFT1, 14-3-3lambda	G-box regulating factor 6	cytosol
AT5G10470.1	KCA1, KAC1	kinesin like protein for actin based chloroplast movement 1	cytosol
AT5G10840.1	Endomembrane protein 70 protein family	Endomembrane protein 70 protein family	golgi
AT5G10860.1	Cystathionine beta-synthase (CBS) family protein	Cystathionine beta-synthase (CBS) family protein	mitochondrion
AT5G11200.1	DEAD/DEAH box RNA helicase family protein	DEAD/DEAH box RNA helicase family protein	nucleus
AT5G11450.1	Mog1/PsbP/DUF1795-like photosystem II reaction center PsbP family protein	Mog1/PsbP/DUF1795-like photosystem II reaction center PsbP family protein	plastid
AT5G11560.1	catalytics	catalytics	endoplasmic reticulum
AT5G11770.1	NADH-ubiquinone oxidoreductase 20 kDa subunit, mitochondrial	NADH-ubiquinone oxidoreductase 20 kDa subunit, mitochondrial	mitochondrion
AT5G12130.1	PDE149, ATTERC	integral membrane TerC family protein	plastid
AT5G12250.1	TUB6	beta-6 tubulin	cytosol
AT5G12470.1	Protein of unknown function (DUF3411)	Protein of unknown function (DUF3411)	plastid
AT5G12860.2	DiT1	dicarboxylate transporter 1	plastid
AT5G13120.2	CYP20-2	cyclophilin 20-2	plastid
AT5G13410.1	FKBP-like peptidyl-prolyl cis-trans isomerase family protein	FKBP-like peptidyl-prolyl cis-trans isomerase family protein	plastid
AT5G13430.1	Ubiquinol-cytochrome C reductase iron-sulfur subunit	Ubiquinol-cytochrome C reductase iron-sulfur subunit	mitochondrion
AT5G13450.1	ATP5	delta subunit of Mt ATP synthase	mitochondrion
AT5G13490.2	AAC2	ADP/ATP carrier 2	mitochondrion
AT5G13510.1	Ribosomal protein L10 family protein	Ribosomal protein L10 family protein	plastid
AT5G13850.1	NACA3	nascent polypeptide-associated complex subunit alpha-like protein 3	cytosol
AT5G14030.4	translocon-associated protein beta (TRAPB) family protein	translocon-associated protein beta (TRAPB) family protein	extracellular
AT5G14040.1	PHT3;1	phosphate transporter 3;1	mitochondrion
AT5G14220.1	HEMG2, MEE61, PPO2	Flavin containing amine oxidoreductase family	mitochondrion,plastid
AT5G14320.1	Ribosomal protein S13/S18 family	Ribosomal protein S13/S18 family	plastid
AT5G14740.2	CA2, CA18, BETA CA2	carbonic anhydrase 2	plastid
AT5G14780.1	FDH	formate dehydrogenase	mitochondrion

Supplementary Table 2 Plastid proteins used for PCA analysis

Accession	Symbol	Name	Subacon Consensus Localization
AT1G01080.1	RNA-binding (RRM/RBD/RNP motifs) family protein	RNA-binding (RRM/RBD/RNP motifs) family protein	plastid
AT1G01790.1	KEA1, ATKEA1	K+ efflux antiporter 1	plastid
AT1G02560.1	CLPP5, NCLPP5, NCLPP1	nuclear encoded CLP protease 5	plastid
AT1G02910.1	LPA1	tetratricopeptide repeat (TPR)-containing protein	plastid
AT1G03160.1	FZL	FZO-like	plastid
AT1G03475.1	LIN2, HEMF1, ATCPO-I	Coproporphyrinogen III oxidase	plastid
AT1G03600.1	PSB27	photosystem II family protein	plastid
AT1G03630.2	POR C, PORC	protochlorophyllide oxidoreductase C	plastid
AT1G03680.1	ATHM1, TRX-M1, ATM1, THM1	thioredoxin M-type 1	plastid
AT1G04620.1	coenzyme F420 hydrogenase family / dehydrogenase, beta subunit family	coenzyme F420 hydrogenase family / dehydrogenase, beta subunit family	plastid
AT1G05190.1	emb2394	Ribosomal protein L6 family	plastid
AT1G06190.1	Rho termination factor	Rho termination factor	plastid
AT1G06430.1	FTSH8	FTSH protease 8	plastid
AT1G06680.2	PSBP-1, OEE2, PSII-P	photosystem II subunit P-1	plastid
AT1G06690.1	NAD(P)-linked oxidoreductase superfamily protein	NAD(P)-linked oxidoreductase superfamily protein	plastid
AT1G06950.1	ATTIC110, TIC110	translocon at the inner envelope membrane of chloroplasts 110	plastid
AT1G07320.1	RPL4	ribosomal protein L4	plastid
AT1G08520.1	ALB1, ALB-1V, V157, PDE166, CHLD	ALBINA 1	plastid
AT1G08550.2	NPQ1, AVDE1	non-photochemical quenching 1	plastid
AT1G08640.1	CJD1	Chloroplast J-like domain 1	plastid
AT1G09340.1	CRB, CSP41B, HIP1.3	chloroplast RNA binding	plastid
AT1G10510.1	emb2004	RNI-like superfamily protein	plastid
AT1G11750.1	CLPP6, NCLPP1, NCLPP6	CLP protease proteolytic subunit 6	plastid
AT1G12250.2	Pentapeptide repeat-containing protein	Pentapeptide repeat-containing protein	plastid
AT1G12900.4	GAPA-2	glyceraldehyde 3-phosphate dehydrogenase A subunit 2	plastid
AT1G14150.1	PQL1, PQL2	PsbQ-like 2	plastid
AT1G14345.1	NAD(P)-linked oxidoreductase superfamily protein	NAD(P)-linked oxidoreductase superfamily protein	plastid
AT1G15820.1	LHCB6, CP24	light harvesting complex photosystem II subunit 6	plastid
AT1G15980.1	NDF1, NDH48	NDH-dependent cyclic electron flow 1	plastid

AT1G16445.1	S-adenosyl-L-methionine-dependent methyltransferases superfamily protein	S-adenosyl-L-methionine-dependent methyltransferases superfamily protein	plastid
AT1G16610.2	SR45, RNPS1	arginine/serine-rich 45	plastid
AT1G16720.1	HCF173	high chlorophyll fluorescence phenotype 173	plastid
AT1G16880.1	uridylyltransferase-related	uridylyltransferase-related	plastid
AT1G17220.1	FUG1	Translation initiation factor 2, small GTP-binding protein	plastid
AT1G18170.1	FKBP-like peptidyl-prolyl cis-trans isomerase family protein	FKBP-like peptidyl-prolyl cis-trans isomerase family protein	plastid
AT1G20020.1	ATLFNR2, FNR2	ferredoxin-NADP(+)-oxidoreductase 2	plastid
AT1G20340.1	DRT112, PETE2	Cupredoxin superfamily protein	plastid
AT1G21500.1	unknown protein;Has 29 Blast hits to 29 proteins in 12 species: Archae - 0;Bacteria - 0;Metazoa - 0;Fungi - 2;Plants - 27;Viruses - 0;Other Eukaryotes - 0 (source: NCBI BLink).	unknown protein;Has 29 Blast hits to 29 proteins in 12 species: Archae - 0;Bacteria - 0;Metazoa - 0;Fungi - 2;Plants - 27;Viruses - 0;Other Eukaryotes - 0 (source: NCBI BLink).	plastid
AT1G22700.3	Tetratricopeptide repeat (TPR)-like superfamily protein	Tetratricopeptide repeat (TPR)-like superfamily protein	plastid
AT1G24490.2	ALB4, ARTEMIS	OxaA/YidC-like membrane insertion protein	plastid
AT1G27400.1	Ribosomal protein L22p/L17e family protein	Ribosomal protein L22p/L17e family protein	plastid,nucleus,cytosol
AT1G28140.1	unknown protein;FUNCTIONS IN: molecular_function unknown;INVOLVED IN: biological_process unknown;LOCATED IN: chloroplast;EXPRESSED IN: 22 plant structures;EXPRESSED DURING: 13 growth stages;CONTAINS InterPro DOMAIN/s: Protei	unknown protein;FUNCTIONS IN: molecular_function unknown;INVOLVED IN: biological_process unknown;LOCATED IN: chloroplast;EXPRESSED IN: 22 plant structures;EXPRESSED DURING: 13 growth stages;CONTAINS InterPro DOMAIN/s: Protei	plastid
AT1G29700.1	Metallo-hydrolase/oxidoreductase superfamily protein	Metallo-hydrolase/oxidoreductase superfamily protein	plastid
AT1G29930.1	CAB1, AB140, CAB140, LHCB1.3	chlorophyll A/B binding protein 1	plastid
AT1G30380.1	PSAK	photosystem I subunit K	plastid
AT1G31330.1	PSAF	photosystem I subunit F	plastid
AT1G32060.1	PRK	phosphoribulokinase	plastid
AT1G32220.1	NAD(P)-binding Rossmann-fold superfamily protein	NAD(P)-binding Rossmann-fold superfamily protein	plastid
AT1G61520.2	LHCA3	photosystem I light harvesting complex gene 3	plastid
AT1G32990.1	PRPL11	plastid ribosomal protein l11	plastid
AT1G34000.1	OHP2	one-helix protein 2	plastid

AT1G34430.1	EMB3003	2-oxoacid dehydrogenases acyltransferase family protein	plastid
AT1G35680.1	Ribosomal protein L21	Ribosomal protein L21	plastid
AT1G42960.1	expressed protein localized to the inner membrane of the chloroplast.	expressed protein localized to the inner membrane of the chloroplast.	plastid
AT1G42970.1	GAPB	glyceraldehyde-3-phosphate dehydrogenase B subunit	plastid
AT1G44575.1	NPQ4, PSBS	Chlorophyll A-B binding family protein	plastid
AT1G45474.2	LHCA5	photosystem I light harvesting complex gene 5	plastid
AT1G49970.1	CLPR1, NCLPP5, SVR2	CLP protease proteolytic subunit 1	plastid
AT1G50250.1	FTSH1	FTSH protease 1	plastid
AT1G50450.1	Saccharopine dehydrogenase	Saccharopine dehydrogenase	plastid
AT1G51100.1	unknown protein;FUNCTIONS IN: molecular_function unknown;INVOLVED IN: biological_process unknown;LOCATED IN: chloroplast, chloroplast stroma;EXPRESSED IN: 22 plant structures;EXPRESSED DURING: 13 growth stages;Has 26 Blast h	unknown protein;FUNCTIONS IN: molecular_function unknown;INVOLVED IN: biological_process unknown;LOCATED IN: chloroplast, chloroplast stroma;EXPRESSED IN: 22 plant structures;EXPRESSED DURING: 13 growth stages;Has 26 Blast h	plastid
AT1G51110.1	Plastid-lipid associated protein PAP / fibrillin family protein	Plastid-lipid associated protein PAP / fibrillin family protein	plastid
AT1G51400.1	Photosystem II 5 kD protein	Photosystem II 5 kD protein	plastid
AT1G52510.1	alpha/beta-Hydrolases superfamily protein	alpha/beta-Hydrolases superfamily protein	plastid
AT1G54350.1	ABC transporter family protein	ABC transporter family protein	plastid
AT1G54500.1	Rubredoxin-like superfamily protein	Rubredoxin-like superfamily protein	plastid
AT1G54520.1	unknown protein;FUNCTIONS IN: molecular_function unknown;INVOLVED IN: biological_process unknown;LOCATED IN: chloroplast;EXPRESSED IN: 24 plant structures;EXPRESSED DURING: 15 growth stages;CONTAINS InterPro DOMAIN/s: Protei	unknown protein;FUNCTIONS IN: molecular_function unknown;INVOLVED IN: biological_process unknown;LOCATED IN: chloroplast;EXPRESSED IN: 24 plant structures;EXPRESSED DURING: 15 growth stages;CONTAINS InterPro DOMAIN/s: Protei	plastid
AT1G54780.1	TLP18.3	thylakoid lumen 18.3 kDa protein	plastid
AT1G55480.1	ZKT	protein containing PDZ domain, a K-box domain, and a TPR region	plastid
AT1G55490.2	CPN60B, LEN1	chaperonin 60 beta	plastid
AT1G55670.1	PSAG	photosystem I subunit G	plastid
AT1G56500.1	haloacid dehalogenase-like hydrolase family protein	haloacid dehalogenase-like hydrolase family protein	plastid

AT1G62750.1	ATSCO1, ATSCO1/CPEF-G, SCO1	Translation elongation factor EFG/EF2 protein	plastid
AT1G63940.4	MDAR6	monodehydroascorbate reductase 6	plastid
AT1G64355.1	unknown protein;FUNCTIONS IN: molecular_function unknown;INVOLVED IN: biological_process unknown;LOCATED IN: chloroplast;EXPRESSED IN: 23 plant structures;EXPRESSED DURING: 13 growth stages;CONTAINS InterPro DOMAIN/s: Protei	unknown protein;FUNCTIONS IN: molecular_function unknown;INVOLVED IN: biological_process unknown;LOCATED IN: chloroplast;EXPRESSED IN: 23 plant structures;EXPRESSED DURING: 13 growth stages;CONTAINS InterPro DOMAIN/s: Protei	plastid
AT1G64770.1	NDF2, NDH45	NDH-dependent cyclic electron flow 1	plastid
AT1G64970.1	G-TMT, TMT1, VTE4	gamma-tocopherol methyltransferase	plastid
AT1G65230.1	Uncharacterized conserved protein (DUF2358)	Uncharacterized conserved protein (DUF2358)	plastid
AT1G65260.1	PTAC4, VIPP1	plastid transcriptionally active 4	plastid
AT1G67090.1	RBCS1A	ribulose bisphosphate carboxylase small chain 1A	plastid
AT1G67700.2	unknown protein;FUNCTIONS IN: molecular_function unknown;INVOLVED IN: biological_process unknown;LOCATED IN: chloroplast, chloroplast envelope;EXPRESSED IN: 22 plant structures;EXPRESSED DURING: 13 growth stages;Has 49 Blast	unknown protein;FUNCTIONS IN: molecular_function unknown;INVOLVED IN: biological_process unknown;LOCATED IN: chloroplast, chloroplast envelope;EXPRESSED IN: 22 plant structures;EXPRESSED DURING: 13 growth stages;Has 49 Blast	plastid
AT1G68590.2	Ribosomal protein PSRP-3/Ycf65	Ribosomal protein PSRP-3/Ycf65	plastid
AT1G68830.1	STN7	STT7 homolog STN7	plastid
AT1G69200.1	FLN2	fructokinase-like 2	plastid
AT1G70070.1	EMB25, PDE317, ISE2	DEAD/DEAH box helicase, putative	plastid
AT1G70760.1	CRR23	inorganic carbon transport protein-related	plastid
AT1G71480.1	Nuclear transport factor 2 (NTF2) family protein	Nuclear transport factor 2 (NTF2) family protein	plastid
AT1G71500.1	Rieske (2Fe-2S) domain-containing protein	Rieske (2Fe-2S) domain-containing protein	plastid
AT1G71810.1	Protein kinase superfamily protein	Protein kinase superfamily protein	plastid
AT1G72640.2	NAD(P)-binding Rossmann-fold superfamily protein	NAD(P)-binding Rossmann-fold superfamily protein	plastid
AT1G73060.1	LPA3	Low PSII Accumulation 3	plastid
AT1G73110.1	P-loop containing nucleoside triphosphate hydrolases superfamily protein	P-loop containing nucleoside triphosphate hydrolases superfamily protein	plastid

AT1G73990.1	SPPA, SPPA1	signal peptide peptidase	plastid
AT1G74070.1	Cyclophilin-like peptidyl-prolyl cis-trans isomerase family protein	Cyclophilin-like peptidyl-prolyl cis-trans isomerase family protein	plastid
AT1G74470.1	Pyridine nucleotide-disulphide oxidoreductase family protein	Pyridine nucleotide-disulphide oxidoreductase family protein	plastid
AT1G74730.1	Protein of unknown function (DUF1118)	Protein of unknown function (DUF1118)	plastid
AT1G74970.1	RPS9, TWN3	ribosomal protein S9	plastid
AT1G75350.1	emb2184	Ribosomal protein L31	plastid
AT1G75690.1	DnaJ/Hsp40 cysteine-rich domain superfamily protein	DnaJ/Hsp40 cysteine-rich domain superfamily protein	plastid
AT1G76450.1	Photosystem II reaction center PsbP family protein	Photosystem II reaction center PsbP family protein	plastid
AT1G77490.1	TAPX	thylakoidal ascorbate peroxidase	plastid
AT1G77590.1	LACS9	long chain acyl-CoA synthetase 9	plastid
AT1G78140.1	S-adenosyl-L-methionine-dependent methyltransferases superfamily protein	S-adenosyl-L-methionine-dependent methyltransferases superfamily protein	plastid
AT1G78915.1	Tetratricopeptide repeat (TPR)-like superfamily protein	Tetratricopeptide repeat (TPR)-like superfamily protein	plastid
AT1G79040.1	PSBR	photosystem II subunit R	plastid
AT1G79560.1	EMB156, EMB36, EMB1047, FTSH12	FTSH protease 12	plastid
AT1G79600.1	Protein kinase superfamily protein	Protein kinase superfamily protein	plastid
AT1G79850.1	RPS17, CS17, PRPS17	ribosomal protein S17	plastid
AT1G80030.3	Molecular chaperone Hsp40/DnaJ family protein	Molecular chaperone Hsp40/DnaJ family protein	plastid
AT1G80300.1	NTT1, ATNTT1	nucleotide transporter 1	plastid
AT1G80380.3	P-loop containing nucleoside triphosphate hydrolases superfamily protein	P-loop containing nucleoside triphosphate hydrolases superfamily protein	plastid
AT2G01110.1	APG2, UNE3, PGA2, TATC	Sec-independent periplasmic protein translocase	plastid
AT2G01140.1	Aldolase superfamily protein	Aldolase superfamily protein	plastid
AT2G03420.1	unknown protein;Has 38 Blast hits to 38 proteins in 17 species: Archae - 0;Bacteria - 0;Metazoa - 0;Fungi - 0;Plants - 38;Viruses - 0;Other Eukaryotes - 0 (source: NCBI BLink).	unknown protein;Has 38 Blast hits to 38 proteins in 17 species: Archae - 0;Bacteria - 0;Metazoa - 0;Fungi - 0;Plants - 38;Viruses - 0;Other Eukaryotes - 0 (source: NCBI BLink).	plastid
AT2G04030.1	CR88, EMB1956, HSP90.5, Hsp88.1, AtHsp90.5	Chaperone protein htpG family protein	plastid
AT2G05070.1	LHCB2.2, LHCB2	photosystem II light harvesting complex gene 2.2	plastid
AT2G05620.1	PGR5	proton gradient regulation 5	plastid
AT2G07698.1	ATPase, F1 complex, alpha subunit protein	ATPase, F1 complex, alpha subunit protein	mitochondrion,plastid
AT2G14880.1	SWIB/MDM2 domain superfamily protein	SWIB/MDM2 domain superfamily protein	plastid

AT2G15290.1	ATTIC21, TIC21, CIA5, PIC1	translocon at inner membrane of chloroplasts 21	plastid
AT2G18710.1	SCY1	SECY homolog 1	plastid
AT2G20260.1	PSAE-2	photosystem I subunit E-2	plastid
AT2G20890.1	PSB29, THF1	photosystem II reaction center PSB29 protein	plastid
AT2G21280.1	GC1, ATSULA, SULA	NAD(P)-binding Rossmann-fold superfamily protein	plastid
AT2G21330.1	FBA1	fructose-bisphosphate aldolase 1	plastid
AT2G21530.1	SMAD/FHA domain-containing protein	SMAD/FHA domain-containing protein	plastid
AT2G21960.1	unknown protein;LOCATED IN: chloroplast;EXPRESSED IN: 22 plant structures;EXPRESSED DURING: 13 growth stages;BEST Arabidopsis thaliana protein match is: unknown protein (TAIR:AT1G56180.1);Has 224 Blast hits to 222 proteins in	unknown protein;LOCATED IN: chloroplast;EXPRESSED IN: 22 plant structures;EXPRESSED DURING: 13 growth stages;BEST Arabidopsis thaliana protein match is: unknown protein (TAIR:AT1G56180.1);Has 224 Blast hits to 222 proteins in	plastid
AT2G22360.1	DNAJ heat shock family protein	DNAJ heat shock family protein	plastid
AT2G23670.1	YCF37	homolog of Synechocystis YCF37	plastid
AT2G24020.2	Uncharacterised BCR, YbaB family COG0718	Uncharacterised BCR, YbaB family COG0718	plastid
AT2G24060.1	Translation initiation factor 3 protein	Translation initiation factor 3 protein	plastid
AT2G24090.1	Ribosomal protein L35	Ribosomal protein L35	plastid
AT2G24820.1	TIC55-II	translocon at the inner envelope membrane of chloroplasts 55-II	plastid
AT2G25080.1	ATGPX1, GPX1	glutathione peroxidase 1	plastid
AT2G26340.1	unknown protein;FUNCTIONS IN: molecular_function unknown;INVOLVED IN: biological_process unknown;LOCATED IN: chloroplast thylakoid membrane, chloroplast thylakoid lumen, chloroplast;EXPRESSED IN: 22 plant structures;EXPRESSED	unknown protein;FUNCTIONS IN: molecular_function unknown;INVOLVED IN: biological_process unknown;LOCATED IN: chloroplast thylakoid membrane, chloroplast thylakoid lumen, chloroplast;EXPRESSED IN: 22 plant structures;EXPRESSED	plastid
AT2G27290.1	Protein of unknown function (DUF1279)	Protein of unknown function (DUF1279)	plastid
AT2G27680.1	NAD(P)-linked oxidoreductase superfamily protein	NAD(P)-linked oxidoreductase superfamily protein	plastid
AT2G28000.1	CPN60A, CH-CPN60A, SLP	chaperonin-60alpha	plastid
AT2G28800.4	ALB3	63 kDa inner membrane family protein	plastid
AT2G28900.1	OEP16, ATOEP16-L, ATOEP16-1, OEP16-1	outer plastid envelope protein 16-1	plastid
AT2G29650.2	ANTR1	phosphate transporter 4;1	plastid
AT2G30390.1	FC2, FC-II, ATFC-II	ferrochelatase 2	plastid
AT2G30950.1	VAR2, FTSH2	FtsH extracellular protease family	plastid

AT2G32640.1	Lycopene beta/epsilon cyclase protein	Lycopene beta/epsilon cyclase protein	plastid
AT2G33450.1	Ribosomal L28 family	Ribosomal L28 family	plastid
AT2G33800.1	Ribosomal protein S5 family protein	Ribosomal protein S5 family protein	plastid
AT2G34420.1	LHB1B2, LHCB1.5	photosystem II light harvesting complex gene B1B2	plastid
AT2G34430.1	LHB1B1, LHCB1.4	light-harvesting chlorophyll-protein complex II subunit B1	plastid
AT2G34460.1	NAD(P)-binding Rossmann-fold superfamily protein	NAD(P)-binding Rossmann-fold superfamily protein	plastid
AT2G35410.1	RNA-binding (RRM/RBD/RNP motifs) family protein	RNA-binding (RRM/RBD/RNP motifs) family protein	plastid
AT2G35490.1	Plastid-lipid associated protein PAP / fibrillin family protein	Plastid-lipid associated protein PAP / fibrillin family protein	plastid
AT2G36145.1	unknown protein;FUNCTIONS IN: molecular_function unknown;INVOLVED IN: biological_process unknown;LOCATED IN: chloroplast thylakoid membrane, chloroplast stroma, chloroplast;EXPRESSED IN: 19 plant structures;EXPRESSED DURING:	unknown protein;FUNCTIONS IN: molecular_function unknown;INVOLVED IN: biological_process unknown;LOCATED IN: chloroplast thylakoid membrane, chloroplast stroma, chloroplast;EXPRESSED IN: 19 plant structures;EXPRESSED DURING:	plastid
AT2G36250.2	FTSZ2-1, ATFTSZ2-1	Tubulin/FtsZ family protein	plastid
AT2G37220.1	RNA-binding (RRM/RBD/RNP motifs) family protein	RNA-binding (RRM/RBD/RNP motifs) family protein	plastid
AT2G37660.1	NAD(P)-binding Rossmann-fold superfamily protein	NAD(P)-binding Rossmann-fold superfamily protein	plastid
AT2G37860.3	LCD1	Protein of unknown function (DUF3411)	plastid
AT2G38040.2	CAC3	acetyl Co-enzyme a carboxylase carboxyltransferase alpha subunit	plastid
AT2G38270.1	CXIP2, ATGRX2	CAX-interacting protein 2	plastid
AT2G38550.1	Transmembrane proteins 14C	Transmembrane proteins 14C	plastid
AT2G39470.1	PPL2	PsbP-like protein 2	plastid
AT2G39730.1	RCA	rubisco activase	plastid
AT2G40100.1	LHCB4.3	light harvesting complex photosystem II	plastid
AT2G42130.3	Plastid-lipid associated protein PAP / fibrillin family protein	Plastid-lipid associated protein PAP / fibrillin family protein	plastid
AT2G42220.1	Rhodanese/Cell cycle control phosphatase superfamily protein	Rhodanese/Cell cycle control phosphatase superfamily protein	plastid
AT2G43030.1	Ribosomal protein L3 family protein	Ribosomal protein L3 family protein	plastid
AT2G43950.1	OEP37, ATOEP37	chloroplast outer envelope protein 37	plastid
AT2G44640.1	FUNCTIONS IN: molecular_function unknown;INVOLVED IN:	FUNCTIONS IN: molecular_function unknown;INVOLVED IN:	plastid

	biological_process unknown;LOCATED IN: mitochondrion, chloroplast, plasma membrane, plastid, chloroplast envelope;EXPRESSED IN: 23 plant structures;EXPRESSED DURING: 13 gro	biological_process unknown;LOCATED IN: mitochondrion, chloroplast, plasma membrane, plastid, chloroplast envelope;EXPRESSED IN: 23 plant structures;EXPRESSED DURING: 13 gro	
AT2G46820.2	PTAC8, TMP14, PSAP, PSI-P	photosystem I P subunit	plastid
AT2G47730.1	ATGSTF8, ATGSTF5, GST6, GSTF8	glutathione S-transferase phi 8	plastid
AT2G47840.1	Uncharacterised conserved protein ycf60	Uncharacterised conserved protein ycf60	plastid
AT3G01440.1	PQL1, PQL2	PsbQ-like 1	plastid
AT3G01480.1	CYP38, ATCYP38	cyclophilin 38	plastid
AT3G01500.1	CA1, ATBCA1, SABP3, ATSABP3	carbonic anhydrase 1	plastid
AT3G03710.1	RIF10, PNP	polyribonucleotide nucleotidyltransferase, putative	plastid
AT3G04260.1	PTAC3	plastid transcriptionally active 3	plastid
AT3G04340.1	emb2458	FtsH extracellular protease family	plastid
AT3G04790.1	Ribose 5-phosphate isomerase, type A protein	Ribose 5-phosphate isomerase, type A protein	plastid
AT3G04870.2	ZDS, PDE181, SPC1	zeta-carotene desaturase	plastid
AT3G06510.1	SFR2, ATSFR2	Glycosyl hydrolase superfamily protein	plastid
AT3G06730.1	TRX P, TRX z	Thioredoxin z	plastid
AT3G08920.1	Rhodanese/Cell cycle control phosphatase superfamily protein	Rhodanese/Cell cycle control phosphatase superfamily protein	plastid
AT3G08940.2	LHCB4.2	light harvesting complex photosystem II	plastid
AT3G09050.1	unknown protein;FUNCTIONS IN: molecular_function unknown;INVOLVED IN: biological_process unknown;LOCATED IN: chloroplast thylakoid membrane, chloroplast;EXPRESSED IN: 21 plant structures;EXPRESSED DURING: 13 growth stages;Ha	unknown protein;FUNCTIONS IN: molecular_function unknown;INVOLVED IN: biological_process unknown;LOCATED IN: chloroplast thylakoid membrane, chloroplast;EXPRESSED IN: 21 plant structures;EXPRESSED DURING: 13 growth stages;Ha	plastid
AT3G09580.1	FAD/NAD(P)-binding oxidoreductase family protein	FAD/NAD(P)-binding oxidoreductase family protein	plastid
AT3G10060.1	FKBP-like peptidyl-prolyl cis-trans isomerase family protein	FKBP-like peptidyl-prolyl cis-trans isomerase family protein	plastid
AT3G10130.1	SOUL heme-binding family protein	SOUL heme-binding family protein	plastid
AT3G10350.1	P-loop containing nucleoside triphosphate hydrolases superfamily protein	P-loop containing nucleoside triphosphate hydrolases superfamily protein	plastid
AT3G10690.1	GYRA	DNA GYRASE A	plastid
AT3G11630.1	Thioredoxin superfamily protein	Thioredoxin superfamily protein	plastid
AT3G11945.1	PDS2, ATHST, HST	homogentisate prenyltransferase	plastid

AT3G12345.1	unknown protein;LOCATED IN: chloroplast;Has 35333 Blast hits to 34131 proteins in 2444 species: Archae - 798;Bacteria - 22429;Metazoa - 974;Fungi - 991;Plants - 531;Viruses - 0;Other Eukaryotes - 9610 (source: NCBI BLink).	unknown protein;LOCATED IN: chloroplast;Has 35333 Blast hits to 34131 proteins in 2444 species: Archae - 798;Bacteria - 22429;Metazoa - 974;Fungi - 991;Plants - 531;Viruses - 0;Other Eukaryotes - 9610 (source: NCBI BLink).	plastid
AT3G12780.1	PGK1	phosphoglycerate kinase 1	plastid
AT3G13120.2	Ribosomal protein S10p/S20e family protein	Ribosomal protein S10p/S20e family protein	plastid
AT3G15110.1	unknown protein;FUNCTIONS IN: molecular_function unknown;INVOLVED IN: biological_process unknown;LOCATED IN: chloroplast thylakoid membrane;EXPRESSED IN: 20 plant structures;EXPRESSED DURING: 13 growth stages;CONTAINS InterP	unknown protein;FUNCTIONS IN: molecular_function unknown;INVOLVED IN: biological_process unknown;LOCATED IN: chloroplast thylakoid membrane;EXPRESSED IN: 20 plant structures;EXPRESSED DURING: 13 growth stages;CONTAINS InterP	plastid
AT3G15190.1	chloroplast 30S ribosomal protein S20, putative	chloroplast 30S ribosomal protein S20, putative	plastid
AT3G15360.1	ATHM4, TRX-M4, ATM4	thioredoxin M-type 4	plastid
AT3G15520.1	Cyclophilin-like peptidyl-prolyl cis-trans isomerase family protein	Cyclophilin-like peptidyl-prolyl cis-trans isomerase family protein	plastid
AT3G16000.1	MFP1	MAR binding filament-like protein 1	plastid
AT3G16140.1	PSAH-1	photosystem I subunit H-1	plastid
AT3G17930.1	unknown protein;FUNCTIONS IN: molecular_function unknown;INVOLVED IN: biological_process unknown;LOCATED IN: chloroplast thylakoid membrane;EXPRESSED IN: 22 plant structures;EXPRESSED DURING: 13 growth stages;CONTAINS InterP	unknown protein;FUNCTIONS IN: molecular_function unknown;INVOLVED IN: biological_process unknown;LOCATED IN: chloroplast thylakoid membrane;EXPRESSED IN: 22 plant structures;EXPRESSED DURING: 13 growth stages;CONTAINS InterP	plastid
AT3G17970.1	atToc64-III, TOC64-III	translocon at the outer membrane of chloroplasts 64-III	plastid
AT3G18890.1	NAD(P)-binding Rossmann-fold superfamily protein	NAD(P)-binding Rossmann-fold superfamily protein	plastid
AT3G19170.1	ATPREP1, ATZNMP, PREP1	presequence protease 1	mitochondrion,plastid
AT3G20320.1	TGD2	trigalactosyldiacylglycerol2	plastid
AT3G20390.1	endoribonuclease L-PSP family protein	endoribonuclease L-PSP family protein	plastid
AT3G21055.1	PSBTN	photosystem II subunit T	plastid
AT3G23400.1	FIB4	Plastid-lipid associated protein PAP / fibrillin family protein	plastid
AT3G24590.1	PLSP1	plastidic type i signal peptidase 1	plastid

AT3G25690.1	CHUP1	Hydroxyproline-rich glycoprotein family protein	plastid
AT3G25760.1	AOC1, ERD12	allene oxide cyclase 1	plastid
AT3G25770.1	AOC2	allene oxide cyclase 2	plastid
AT3G25860.1	LTA2, PLE2	2-oxoacid dehydrogenases acyltransferase family protein	plastid
AT3G25920.1	RPL15	ribosomal protein L15	plastid
AT3G26060.1	ATPRX Q	Thioredoxin superfamily protein	plastid
AT3G26070.1	Plastid-lipid associated protein PAP / fibrillin family protein	Plastid-lipid associated protein PAP / fibrillin family protein	plastid
AT3G26650.1	GAPA, GAPA-1	glyceraldehyde 3-phosphate dehydrogenase A subunit	plastid
AT3G26710.1	CCB1	cofactor assembly of complex C	plastid
AT3G26740.1	CCL	CCR-like	plastid
AT3G27820.1	ATMDAR4, MDAR4	monodehydroascorbate reductase 4	plastid,peroxisome
AT3G27850.1	RPL12-C	ribosomal protein L12-C	plastid
AT3G27925.1	DEGP1, Deg1	DegP protease 1	plastid
AT3G43520.1	Transmembrane proteins 14C	Transmembrane proteins 14C	plastid
AT3G44880.1	ACD1, LLS1, PAO	Pheophorbide a oxygenase family protein with Rieske [2Fe-2S] domain	plastid
AT3G44890.1	RPL9	ribosomal protein L9	plastid
AT3G45140.1	LOX2, ATLOX2	lipoxygenase 2	plastid
AT3G45780.2	PHOT1, NPH1, JK224, RPT1	phototropin 1	plastid
AT3G46740.1	TOC75-III, MAR1	translocon at the outer envelope membrane of chloroplasts 75-III	plastid
AT3G46780.1	PTAC16	plastid transcriptionally active 16	plastid
AT3G47070.1	LOCATED IN: thylakoid, chloroplast thylakoid membrane, chloroplast, chloroplast envelope;EXPRESSED IN: 22 plant structures;EXPRESSED DURING: 13 growth stages;CONTAINS InterPro DOMAIN/s: Thylakoid soluble phosphoprotein TSP9 (In	LOCATED IN: thylakoid, chloroplast thylakoid membrane, chloroplast, chloroplast envelope;EXPRESSED IN: 22 plant structures;EXPRESSED DURING: 13 growth stages;CONTAINS InterPro DOMAIN/s: Thylakoid soluble phosphoprotein TSP9 (In	plastid
AT3G47470.1	LHCA4, CAB4	light-harvesting chlorophyll-protein complex I subunit A4	plastid
AT3G47520.1	MDH	malate dehydrogenase	plastid
AT3G47860.1	CHL	chloroplastic lipocalin	plastid
AT3G48500.1	PDE312, PTAC10	Nucleic acid-binding, OB-fold-like protein	plastid
AT3G48730.1	GSA2	glutamate-1-semialdehyde 2,1-aminomutase 2	plastid
AT3G51140.1	Protein of unknown function (DUF3353)	Protein of unknown function (DUF3353)	plastid
AT3G51820.1	ATG4, G4, CHLG	UbiA prenyltransferase family protein	plastid

AT3G52150.2	RNA-binding (RRM/RBD/RNP motifs) family protein	RNA-binding (RRM/RBD/RNP motifs) family protein	plastid
AT3G52230.1	unknown protein;FUNCTIONS IN: molecular_function unknown;INVOLVED IN: biological_process unknown;LOCATED IN: chloroplast outer membrane, chloroplast thylakoid membrane, chloroplast, chloroplast envelope;EXPRESSED IN: 24 plant	unknown protein;FUNCTIONS IN: molecular_function unknown;INVOLVED IN: biological_process unknown;LOCATED IN: chloroplast outer membrane, chloroplast thylakoid membrane, chloroplast, chloroplast envelope;EXPRESSED IN: 24 plant	plastid
AT3G52380.1	CP33, PDE322	chloroplast RNA-binding protein 33	plastid
AT3G52960.1	Thioredoxin superfamily protein	Thioredoxin superfamily protein	plastid
AT3G53130.1	LUT1, CYP97C1	Cytochrome P450 superfamily protein	plastid
AT3G53470.1	unknown protein;FUNCTIONS IN: molecular_function unknown;INVOLVED IN: biological_process unknown;LOCATED IN: chloroplast thylakoid membrane, chloroplast;EXPRESSED IN: 22 plant structures;EXPRESSED DURING: 13 growth stages;Ha	unknown protein;FUNCTIONS IN: molecular_function unknown;INVOLVED IN: biological_process unknown;LOCATED IN: chloroplast thylakoid membrane, chloroplast;EXPRESSED IN: 22 plant structures;EXPRESSED DURING: 13 growth stages;Ha	plastid
AT3G54210.1	Ribosomal protein L17 family protein	Ribosomal protein L17 family protein	plastid
AT3G54890.4	LHCA1	photosystem I light harvesting complex gene 1	plastid
AT3G55330.1	PPL1	PsbP-like protein 1	plastid
AT3G55800.1	SBPASE	sedoheptulose-bisphosphatase	plastid
AT3G56650.1	Mog1/PsbP/DUF1795-like photosystem II reaction center PsbP family protein	Mog1/PsbP/DUF1795-like photosystem II reaction center PsbP family protein	plastid
AT3G56910.1	PSRP5	plastid-specific 50S ribosomal protein 5	plastid
AT3G56940.1	CRD1, CHL27, ACSF	dicarboxylate diiron protein, putative (Crd1)	plastid
AT3G58010.1	PGL34	plastoglobulin 34kD	plastid
AT3G58610.3	ketol-acid reductoisomerase	ketol-acid reductoisomerase	plastid
AT3G59780.1	Rhodanese/Cell cycle control phosphatase superfamily protein	Rhodanese/Cell cycle control phosphatase superfamily protein	plastid
AT3G60750.2	Transketolase	Transketolase	plastid
AT3G61470.1	LHCA2	photosystem I light harvesting complex gene 2	plastid
AT3G61870.1	unknown protein;FUNCTIONS IN: molecular_function unknown;INVOLVED IN: biological_process unknown;LOCATED IN: chloroplast, chloroplast inner	unknown protein;FUNCTIONS IN: molecular_function unknown;INVOLVED IN: biological_process unknown;LOCATED IN: chloroplast, chloroplast inner	plastid

	membrane, chloroplast envelope;EXPRESSED IN: 23 plant structures;EXPRESSED DURING: 14	membrane, chloroplast envelope;EXPRESSED IN: 23 plant structures;EXPRESSED DURING: 14	
AT3G62030.3	ROC4	rotamase CYP 4	plastid
AT3G63140.1	CSP41A	chloroplast stem-loop binding protein of 41 kDa	plastid
AT3G63160.1	FUNCTIONS IN: molecular_function unknown;INVOLVED IN: biological_process unknown;LOCATED IN: chloroplast outer membrane, thylakoid, chloroplast thylakoid membrane, chloroplast, chloroplast envelope;EXPRESSED IN: 21 plant struct	FUNCTIONS IN: molecular_function unknown;INVOLVED IN: biological_process unknown;LOCATED IN: chloroplast outer membrane, thylakoid, chloroplast thylakoid membrane, chloroplast, chloroplast envelope;EXPRESSED IN: 21 plant struct	plastid
AT3G63190.1	RRF, HFP108, cpRRF, AtcpRRF	ribosome recycling factor, chloroplast precursor	plastid
AT3G63410.1	APG1, VTE3, IEP37, E37	S-adenosyl-L-methionine-dependent methyltransferases superfamily protein	plastid
AT3G63490.1	Ribosomal protein L1p/L10e family	Ribosomal protein L1p/L10e family	plastid
AT4G01050.1	TROL	thylakoid rhodanese-like	plastid
AT4G01150.1	unknown protein;FUNCTIONS IN: molecular_function unknown;INVOLVED IN: biological_process unknown;LOCATED IN: thylakoid, chloroplast thylakoid membrane, chloroplast, plastoglobule, chloroplast envelope;EXPRESSED IN: 23 plant st	unknown protein;FUNCTIONS IN: molecular_function unknown;INVOLVED IN: biological_process unknown;LOCATED IN: thylakoid, chloroplast thylakoid membrane, chloroplast, plastoglobule, chloroplast envelope;EXPRESSED IN: 23 plant st	plastid
AT4G01310.1	Ribosomal L5P family protein	Ribosomal L5P family protein	plastid
AT4G01690.1	PPOX, HEMG1, PPO1	Flavin containing amine oxidoreductase family	plastid
AT4G01800.1	AGY1, AtcpSecA, SECA1	Albino or Glassy Yellow 1	plastid
AT4G02510.1	TOC159, TOC86, PPI2, TOC160, ATTOC159	translocon at the outer envelope membrane of chloroplasts 159	plastid
AT4G02530.1	chloroplast thylakoid lumen protein	chloroplast thylakoid lumen protein	plastid
AT4G02725.1	unknown protein;FUNCTIONS IN: molecular_function unknown;INVOLVED IN: biological_process unknown;LOCATED IN: chloroplast, membrane;Has 35333 Blast hits to 34131 proteins in 2444 species: Archae - 798;Bacteria - 22429;Metazoa	unknown protein;FUNCTIONS IN: molecular_function unknown;INVOLVED IN: biological_process unknown;LOCATED IN: chloroplast, membrane;Has 35333 Blast hits to 34131 proteins in 2444 species: Archae - 798;Bacteria - 22429;Metazoa	plastid

AT4G02770.1	PSAD-1	photosystem I subunit D-1	plastid
AT4G03280.2	PETC, PGR1	photosynthetic electron transfer C	plastid
AT4G03520.1	ATHM2	Thioredoxin superfamily protein	plastid
AT4G04020.1	FIB	fibrillin	plastid
AT4G04640.1	ATPC1	ATPase, F1 complex, gamma subunit protein	plastid
AT4G05180.1	PSBQ, PSBQ-2, PSII-Q	photosystem II subunit Q-2	plastid
AT4G09010.1	APX4, TL29	ascorbate peroxidase 4	plastid
AT4G09040.2	RNA-binding (RRM/RBD/RNP motifs) family protein	RNA-binding (RRM/RBD/RNP motifs) family protein	plastid
AT4G09650.1	ATPD	ATP synthase delta-subunit gene	plastid
AT4G10000.2	Thioredoxin family protein	Thioredoxin family protein	plastid
AT4G10340.1	LHCB5	light harvesting complex of photosystem II 5	plastid
AT4G10750.1	Phosphoenolpyruvate carboxylase family protein	Phosphoenolpyruvate carboxylase family protein	plastid
AT4G11960.1	PGRL1B	PGR5-like B	plastid
AT4G12060.1	Double Clp-N motif protein	Double Clp-N motif protein	plastid
AT4G12800.1	PSAL	photosystem I subunit l	plastid
AT4G13200.1	unknown protein;FUNCTIONS IN: molecular_function unknown;INVOLVED IN: biological_process unknown;LOCATED IN: thylakoid, chloroplast thylakoid membrane, chloroplast, plastoglobule;EXPRESSED IN: 22 plant structures;EXPRESSED DU	unknown protein;FUNCTIONS IN: molecular_function unknown;INVOLVED IN: biological_process unknown;LOCATED IN: thylakoid, chloroplast thylakoid membrane, chloroplast, plastoglobule;EXPRESSED IN: 22 plant structures;EXPRESSED DU	plastid
AT4G13220.1	unknown protein;FUNCTIONS IN: molecular_function unknown;INVOLVED IN: biological_process unknown;LOCATED IN: chloroplast;EXPRESSED IN: 22 plant structures;EXPRESSED DURING: 13 growth stages;Has 27 Blast hits to 27 proteins i	unknown protein;FUNCTIONS IN: molecular_function unknown;INVOLVED IN: biological_process unknown;LOCATED IN: chloroplast;EXPRESSED IN: 22 plant structures;EXPRESSED DURING: 13 growth stages;Has 27 Blast hits to 27 proteins i	plastid
AT4G13670.1	PTAC5	plastid transcriptionally active 5	plastid
AT4G14070.1	AAE15	acyl-activating enzyme 15	plastid
AT4G14210.2	PDS3, PDS, PDE226	phytoene desaturase 3	plastid
AT4G14870.1	SECE1	secE/sec61-gamma protein transport protein	plastid
AT4G15110.1	CYP97B3	cytochrome P450, family 97, subfamily B, polypeptide 3	plastid
AT4G15510.3	Photosystem II reaction center PsbP family protein	Photosystem II reaction center PsbP family protein	plastid
AT4G16155.1	dihydrolipoyl dehydrogenases	dihydrolipoyl dehydrogenases	plastid
AT4G17090.1	CT-BMY, BAM3, BMY8	chloroplast beta-amylase	plastid

AT4G17600.1	LIL3	Chlorophyll A-B binding family protein	plastid
AT4G18370.1	DEG5, DEGP5, HHOA	DEGP protease 5	plastid
AT4G18480.1	CHLI1, CH42, CH-42, CHL11, CHLI-1	P-loop containing nucleoside triphosphate hydrolases superfamily protein	plastid
AT4G18810.1	NAD(P)-binding Rossmann-fold superfamily protein	NAD(P)-binding Rossmann-fold superfamily protein	plastid
AT4G19170.1	NCED4, CCD4	nine-cis-epoxycarotenoid dioxygenase 4	plastid
AT4G20130.1	PTAC14	plastid transcriptionally active 14	plastid
AT4G20360.1	ATRAB8D, ATRABE1B, RABE1b	RAB GTPase homolog E1B	plastid
AT4G21280.1	PSBQ, PSBQA, PSBQ-1	photosystem II subunit QA	plastid
AT4G22240.1	Plastid-lipid associated protein PAP / fibrillin family protein	Plastid-lipid associated protein PAP / fibrillin family protein	plastid
AT4G22890.3	PGR5-LIKE A	PGR5-LIKE A	plastid
AT4G23890.1	unknown protein;FUNCTIONS IN: molecular_function unknown;INVOLVED IN: biological_process unknown;LOCATED IN: chloroplast thylakoid membrane, chloroplast;EXPRESSED IN: 22 plant structures;EXPRESSED DURING: 13 growth stages;CO	unknown protein;FUNCTIONS IN: molecular_function unknown;INVOLVED IN: biological_process unknown;LOCATED IN: chloroplast thylakoid membrane, chloroplast;EXPRESSED IN: 22 plant structures;EXPRESSED DURING: 13 growth stages;CO	plastid
AT4G24280.1	cpHsc70-1	chloroplast heat shock protein 70-1	plastid
AT4G24750.1	Rhodanese/Cell cycle control phosphatase superfamily protein	Rhodanese/Cell cycle control phosphatase superfamily protein	plastid
AT4G24770.1	RBP31, ATRBP31, CP31, ATRBP33	31-kDa RNA binding protein	plastid
AT4G25130.1	PMSR4	peptide met sulfoxide reductase 4	plastid
AT4G25450.3	ATNAP8, NAP8	non-intrinsic ABC protein 8	plastid
AT4G25650.1	ACD1-LIKE, PTC52, TIC55-IV	ACD1-like	plastid
AT4G26530.1	Aldolase superfamily protein	Aldolase superfamily protein	plastid
AT4G27440.2	PORB	protochlorophyllide oxidoreductase B	plastid
AT4G27700.1	Rhodanese/Cell cycle control phosphatase superfamily protein	Rhodanese/Cell cycle control phosphatase superfamily protein	plastid
AT4G28660.1	PSB28	photosystem II reaction center PSB28 protein	plastid
AT4G28750.1	PSAE-1	Photosystem I reaction centre subunit IV / PsaE protein	plastid
AT4G29060.1	emb2726	elongation factor Ts family protein	plastid
AT4G31390.1	Protein kinase superfamily protein	Protein kinase superfamily protein	plastid
AT4G31530.1	NAD(P)-binding Rossmann-fold superfamily protein	NAD(P)-binding Rossmann-fold superfamily protein	plastid

AT4G32260.1	ATPase, F0 complex, subunit B/B, bacterial/chloroplast	ATPase, F0 complex, subunit B/B, bacterial/chloroplast	plastid
AT4G32770.1	VTE1, ATSDX1	tocopherol cyclase, chloroplast / vitamin E deficient 1 (VTE1) / sucrose export defective 1 (SXD1)	plastid
AT4G33350.1	Tic22-like family protein	Tic22-like family protein	plastid
AT4G33500.1	Protein phosphatase 2C family protein	Protein phosphatase 2C family protein	plastid
AT4G34240.1	ALDH3I1, ALDH3	aldehyde dehydrogenase 3I1	plastid
AT4G34620.1	SSR16	small subunit ribosomal protein 16	plastid
AT4G35250.1	NAD(P)-binding Rossmann-fold superfamily protein	NAD(P)-binding Rossmann-fold superfamily protein	plastid
AT4G35760.1	NAD(P)H dehydrogenase (quinone)s	NAD(P)H dehydrogenase (quinone)s	plastid
AT4G36530.1	alpha/beta-Hydrolases superfamily protein	alpha/beta-Hydrolases superfamily protein	plastid
AT4G37200.1	HCF164	Thioredoxin superfamily protein	plastid
AT4G37925.1	NDH-M	subunit NDH-M of NAD(P)H:plastoquinone dehydrogenase complex	plastid
AT4G38970.1	FBA2	fructose-bisphosphate aldolase 2	plastid
AT4G39460.1	SAMC1, SAMT1	S-adenosylmethionine carrier 1	plastid
AT4G39710.1	FKBP16-2	FK506-binding protein 16-2	plastid
AT4G39730.1	Lipase/lipooxygenase, PLAT/LH2 family protein	Lipase/lipooxygenase, PLAT/LH2 family protein	plastid
AT4G39960.1	Molecular chaperone Hsp40/DnaJ family protein	Molecular chaperone Hsp40/DnaJ family protein	plastid
AT5G01220.1	SQD2	sulfoquinovosyldiacylglycerol 2	plastid
AT5G01530.1	LHCB4.1	light harvesting complex photosystem II	plastid
AT5G01590.1	unknown protein;FUNCTIONS IN: molecular_function unknown;INVOLVED IN: biological_process unknown;LOCATED IN: chloroplast, chloroplast envelope;EXPRESSED IN: 22 plant structures;EXPRESSED DURING: 13 growth stages;Has 60 Blast	unknown protein;FUNCTIONS IN: molecular_function unknown;INVOLVED IN: biological_process unknown;LOCATED IN: chloroplast, chloroplast envelope;EXPRESSED IN: 22 plant structures;EXPRESSED DURING: 13 growth stages;Has 60 Blast	plastid
AT5G01920.1	STN8	Protein kinase superfamily protein	plastid
AT5G02120.1	OHP	one helix protein	plastid
AT5G02160.1	unknown protein;FUNCTIONS IN: molecular_function unknown;INVOLVED IN: biological_process unknown;LOCATED IN: chloroplast thylakoid membrane;EXPRESSED IN: 23	unknown protein;FUNCTIONS IN: molecular_function unknown;INVOLVED IN: biological_process unknown;LOCATED IN: chloroplast thylakoid membrane;EXPRESSED IN: 23	plastid

	plant structures;EXPRESSED DURING: 13 growth stages;Has 121 Blast h	plant structures;EXPRESSED DURING: 13 growth stages;Has 121 Blast h	
AT5G02940.1	Protein of unknown function (DUF1012)	Protein of unknown function (DUF1012)	plastid
AT5G03880.1	Thioredoxin family protein	Thioredoxin family protein	plastid
AT5G03900.2	Iron-sulphur cluster biosynthesis family protein	Iron-sulphur cluster biosynthesis family protein	plastid
AT5G03940.1	FFC, 54CP, CPSRP54, SRP54CP	chloroplast signal recognition particle 54 kDa subunit	plastid
AT5G04140.1	GLU1, GLS1, GLUS, FD-GOGAT	glutamate synthase 1	plastid
AT5G04900.1	NOL	NYC1-like	plastid
AT5G05740.3	EGY2	ethylene-dependent gravitropism-deficient and yellow-green-like 2	plastid
AT5G06290.1	2-Cys Prx B, 2CPB	2-cysteine peroxiredoxin B	plastid
AT5G07020.1	proline-rich family protein	proline-rich family protein	plastid
AT5G08050.1	Protein of unknown function (DUF1118)	Protein of unknown function (DUF1118)	plastid
AT5G08280.1	HEMC	hydroxymethylbilane synthase	plastid
AT5G08540.1	unknown protein;FUNCTIONS IN: molecular_function unknown;INVOLVED IN: biological_process unknown;LOCATED IN: chloroplast thylakoid membrane, chloroplast, chloroplast envelope;EXPRESSED IN: 24 plant structures;EXPRESSED DURING	unknown protein;FUNCTIONS IN: molecular_function unknown;INVOLVED IN: biological_process unknown;LOCATED IN: chloroplast thylakoid membrane, chloroplast, chloroplast envelope;EXPRESSED IN: 24 plant structures;EXPRESSED DURING	plastid
AT5G08740.1	NDC1	NAD(P)H dehydrogenase C1	mitochondrion,plastid
AT5G11450.1	Mog1/PsbP/DUF1795-like photosystem II reaction center PsbP family protein	Mog1/PsbP/DUF1795-like photosystem II reaction center PsbP family protein	plastid
AT5G12130.1	PDE149, ATTERC	integral membrane TerC family protein	plastid
AT5G12470.1	Protein of unknown function (DUF3411)	Protein of unknown function (DUF3411)	plastid
AT5G12860.2	DiT1	dicarboxylate transporter 1	plastid
AT5G13120.2	CYP20-2	cyclophilin 20-2	plastid
AT5G13410.1	FKBP-like peptidyl-prolyl cis-trans isomerase family protein	FKBP-like peptidyl-prolyl cis-trans isomerase family protein	plastid
AT5G13510.1	Ribosomal protein L10 family protein	Ribosomal protein L10 family protein	plastid
AT5G14220.1	HEMG2, MEE61, PPO2	Flavin containing amine oxidoreductase family	mitochondrion,plastid
AT5G14320.1	Ribosomal protein S13/S18 family	Ribosomal protein S13/S18 family	plastid
AT5G14740.2	CA2, CA18, BETA CA2	carbonic anhydrase 2	plastid
AT5G16620.1	PDE120, TIC40, ATTIC40	hydroxyproline-rich glycoprotein family protein	plastid

AT5G16660.2	unknown protein;FUNCTIONS IN: molecular_function unknown;INVOLVED IN: biological_process unknown;LOCATED IN: chloroplast, membrane;EXPRESSED IN: 23 plant structures;EXPRESSED DURING: 14 growth stages;BEST Arabidopsis thalian	unknown protein;FUNCTIONS IN: molecular_function unknown;INVOLVED IN: biological_process unknown;LOCATED IN: chloroplast, membrane;EXPRESSED IN: 23 plant structures;EXPRESSED DURING: 14 growth stages;BEST Arabidopsis thalian	plastid
AT5G17170.1	ENH1	rubredoxin family protein	plastid
AT5G18660.1	PCB2	NAD(P)-binding Rossmann-fold superfamily protein	plastid
AT5G19620.1	EMB213, OEP80, ATOEP80, TOC75	outer envelope protein of 80 kDa	plastid
AT5G19940.1	Plastid-lipid associated protein PAP / fibrillin family protein	Plastid-lipid associated protein PAP / fibrillin family protein	plastid
AT5G20140.1	SOUL heme-binding family protein	SOUL heme-binding family protein	plastid
AT5G20720.3	CPN20	chaperonin 20	plastid
AT5G21430.1	Chaperone DnaJ-domain superfamily protein	Chaperone DnaJ-domain superfamily protein	plastid
AT5G22830.1	ATMGT10, GMN10, MGT10, MRS2-11	magnesium (Mg) transporter 10	plastid
AT5G23060.1	CaS	calcium sensing receptor	plastid
AT5G23120.1	HCF136	photosystem II stability/assembly factor, chloroplast (HCF136)	plastid
AT5G23890.1	LOCATED IN: mitochondrion, chloroplast thylakoid membrane, chloroplast, plastid, chloroplast envelope;EXPRESSED IN: 24 plant structures;EXPRESSED DURING: 14 growth stages;CONTAINS InterPro DOMAIN/s: S-layer homology domain (Int	LOCATED IN: mitochondrion, chloroplast thylakoid membrane, chloroplast, plastid, chloroplast envelope;EXPRESSED IN: 24 plant structures;EXPRESSED DURING: 14 growth stages;CONTAINS InterPro DOMAIN/s: S-layer homology domain (Int	plastid
AT5G24300.2	SSI1	Glycogen/starch synthases, ADP-glucose type	plastid
AT5G24490.1	30S ribosomal protein, putative	30S ribosomal protein, putative	plastid
AT5G24690.1	Protein of unknown function (DUF3411)	Protein of unknown function (DUF3411)	plastid
AT5G26742.2	emb1138	DEAD box RNA helicase (RH3)	plastid
AT5G27290.1	unknown protein;LOCATED IN: chloroplast;EXPRESSED IN: 22 plant structures;EXPRESSED DURING: 13 growth stages;BEST Arabidopsis thaliana protein match is: unknown protein (TAIR:AT1G54680.3);Has 30201 Blast hits to 17322 protein	unknown protein;LOCATED IN: chloroplast;EXPRESSED IN: 22 plant structures;EXPRESSED DURING: 13 growth stages;BEST Arabidopsis thaliana protein match is: unknown protein (TAIR:AT1G54680.3);Has 30201 Blast hits to 17322 protein	plastid

AT5G28750.1	Bacterial sec-independent translocation protein mttA/Hcf106	Bacterial sec-independent translocation protein mttA/Hcf106	plastid
AT5G30510.1	RPS1, ARRPS1	ribosomal protein S1	plastid
AT5G33320.1	CUE1, PPT, ARAPPT	Glucose-6-phosphate/phosphate translocator-related	plastid
AT5G35100.1	Cyclophilin-like peptidyl-prolyl cis-trans isomerase family protein	Cyclophilin-like peptidyl-prolyl cis-trans isomerase family protein	plastid
AT5G35170.2	adenylate kinase family protein	adenylate kinase family protein	plastid
AT5G35360.1	CAC2	acetyl Co-enzyme a carboxylase biotin carboxylase subunit	plastid
AT5G35630.3	GS2, GLN2, ATGSL1	glutamine synthetase 2	plastid
AT5G35970.1	P-loop containing nucleoside triphosphate hydrolases superfamily protein	P-loop containing nucleoside triphosphate hydrolases superfamily protein	plastid
AT5G37360.1	unknown protein;FUNCTIONS IN: molecular_function unknown;INVOLVED IN: biological_process unknown;LOCATED IN: chloroplast thylakoid membrane, chloroplast;EXPRESSED IN: 23 plant structures;EXPRESSED DURING: 13 growth stages;Ha	unknown protein;FUNCTIONS IN: molecular_function unknown;INVOLVED IN: biological_process unknown;LOCATED IN: chloroplast thylakoid membrane, chloroplast;EXPRESSED IN: 23 plant structures;EXPRESSED DURING: 13 growth stages;Ha	plastid
AT5G38420.1	Ribulose bisphosphate carboxylase (small chain) family protein	Ribulose bisphosphate carboxylase (small chain) family protein	plastid
AT5G38660.1	APE1	acclimation of photosynthesis to environment	plastid
AT5G39830.2	DEGP8, DEG8	Trypsin family protein with PDZ domain	plastid
AT5G40950.1	RPL27	ribosomal protein large subunit 27	plastid
AT5G42070.1	unknown protein;FUNCTIONS IN: molecular_function unknown;INVOLVED IN: biological_process unknown;LOCATED IN: chloroplast thylakoid membrane, chloroplast;EXPRESSED IN: 21 plant structures;EXPRESSED DURING: 13 growth stages;Ha	unknown protein;FUNCTIONS IN: molecular_function unknown;INVOLVED IN: biological_process unknown;LOCATED IN: chloroplast thylakoid membrane, chloroplast;EXPRESSED IN: 21 plant structures;EXPRESSED DURING: 13 growth stages;Ha	plastid
AT5G42270.1	VAR1, FTSH5	FtsH extracellular protease family	plastid
AT5G42480.1	ARC6	Chaperone DnaJ-domain superfamily protein	plastid
AT5G42650.1	AOS, CYP74A, DDE2	allene oxide synthase	plastid
AT5G42765.1	INVOLVED IN: biological_process unknown;LOCATED IN: thylakoid, chloroplast thylakoid membrane, chloroplast;EXPRESSED IN: 22 plant structures;EXPRESSED	INVOLVED IN: biological_process unknown;LOCATED IN: thylakoid, chloroplast thylakoid membrane, chloroplast;EXPRESSED IN: 22 plant structures;EXPRESSED	plastid

	DURING: 13 growth stages;CONTAINS InterPro DOMAIN/s: Twin-arginine transloc	DURING: 13 growth stages;CONTAINS InterPro DOMAIN/s: Twin-arginine transloc	
AT5G42960.1	unknown protein;FUNCTIONS IN: molecular_function unknown;INVOLVED IN: biological_process unknown;LOCATED IN: mitochondrion, chloroplast, plastid, chloroplast envelope;EXPRESSED IN: 24 plant structures;EXPRESSED DURING: 13 gro	unknown protein;FUNCTIONS IN: molecular_function unknown;INVOLVED IN: biological_process unknown;LOCATED IN: mitochondrion, chloroplast, plastid, chloroplast envelope;EXPRESSED IN: 24 plant structures;EXPRESSED DURING: 13 gro	plastid
AT5G44650.1	CEST, AtCEST	Encodes a chloroplast protein that induces tolerance to multiple environmental stresses and reduces photooxidative damage.	plastid
AT5G45390.1	CLPP4, NCLPP4	CLP protease P4	plastid
AT5G46290.2	KASI, KAS1	3-ketoacyl-acyl carrier protein synthase I	plastid
AT5G47110.1	LIL3	Chlorophyll A-B binding family protein	plastid
AT5G47190.1	Ribosomal protein L19 family protein	Ribosomal protein L19 family protein	plastid
AT5G48300.1	ADG1, APS1	ADP glucose pyrophosphorylase 1	plastid
AT5G48790.1	Domain of unknown function (DUF1995)	Domain of unknown function (DUF1995)	plastid
AT5G49910.1	CPHSC70-2EAT SHOCK PROTEIN 70-2, HSC70-7, cpHsc70-2	chloroplast heat shock protein 70-2	plastid
AT5G50640.1	CBS / octicosapeptide/Phox/Bemp1 (PB1) domains-containing protein	CBS / octicosapeptide/Phox/Bemp1 (PB1) domains-containing protein	plastid
AT5G50920.1	CLPC, ATHSP93-V, HSP93-V, DCA1, CLPC1	CLPC homologue 1	plastid
AT5G51010.1	Rubredoxin-like superfamily protein	Rubredoxin-like superfamily protein	plastid
AT5G51545.1	LPA2	low psii accumulation2	plastid
AT5G52110.2	HCF208	Protein of unknown function (DUF2930)	plastid
AT5G52440.1	HCF106	Bacterial sec-independent translocation protein mttA/Hcf106	plastid
AT5G53480.1	ARM repeat superfamily protein	ARM repeat superfamily protein	plastid,nucleus,cytosol
AT5G54270.1	LHCB3, LHCB3*1	light-harvesting chlorophyll B-binding protein 3	plastid
AT5G54600.1	Translation protein SH3-like family protein	Translation protein SH3-like family protein	plastid
AT5G55220.1	trigger factor type chaperone family protein	trigger factor type chaperone family protein	plastid

AT5G55280.1	FTSZ1-1, ATFTSZ1-1, CPFTSZ	homolog of bacterial cytokinesis Z-ring protein FTSZ 1-1	plastid
AT5G55710.1	FUNCTIONS IN: molecular_function unknown;INVOLVED IN: biological_process unknown;LOCATED IN: chloroplast thylakoid membrane, chloroplast;EXPRESSED IN: 23 plant structures;EXPRESSED DURING: 14 growth stages;BEST Arabidopsis th	FUNCTIONS IN: molecular_function unknown;INVOLVED IN: biological_process unknown;LOCATED IN: chloroplast thylakoid membrane, chloroplast;EXPRESSED IN: 23 plant structures;EXPRESSED DURING: 14 growth stages;BEST Arabidopsis th	plastid
AT5G57030.1	LUT2	Lycopene beta/epsilon cyclase protein	plastid
AT5G58260.1	oxidoreductases, acting on NADH or NADPH, quinone or similar compound as acceptor	oxidoreductases, acting on NADH or NADPH, quinone or similar compound as acceptor	plastid
AT5G58330.3	lactate/malate dehydrogenase family protein	lactate/malate dehydrogenase family protein	plastid
AT5G61670.2	Encodes a close homolog of the Cauliflower OR (Orange) protein. The function of OR is to induce the differentiation of proplastids or other noncolored plastids into chromoplasts for carotenoid accumulation. Both proteins contain	Encodes a close homolog of the Cauliflower OR (Orange) protein. The function of OR is to induce the differentiation of proplastids or other noncolored plastids into chromoplasts for carotenoid accumulation. Both proteins contain	plastid
AT5G62140.1	unknown protein;FUNCTIONS IN: molecular_function unknown;INVOLVED IN: biological_process unknown;LOCATED IN: chloroplast;EXPRESSED IN: 19 plant structures;EXPRESSED DURING: 13 growth stages;Has 60 Blast hits to 60 proteins i	unknown protein;FUNCTIONS IN: molecular_function unknown;INVOLVED IN: biological_process unknown;LOCATED IN: chloroplast;EXPRESSED IN: 19 plant structures;EXPRESSED DURING: 13 growth stages;Has 60 Blast hits to 60 proteins i	plastid
AT5G63420.1	emb2746	RNA-metabolising metallo-beta-lactamase family protein	plastid
AT5G64040.1	PSAN	photosystem I reaction center subunit PSI-N, chloroplast, putative / PSI-N, putative (PSAN)	plastid
AT5G64290.1	DCT, DIT2.1	dicarboxylate transport 2.1	plastid
AT5G65220.1	Ribosomal L29 family protein	Ribosomal L29 family protein	plastid
AT5G66190.1	ATLFNR1, FNR1	ferredoxin-NADP(+)-oxidoreductase 1	plastid
AT5G67030.1	ABA1, LOS6, NPQ2, ATABA1, ZEP, IBS3, ATZEP	zeaxanthin epoxidase (ZEP) (ABA1)	plastid
ATCG00020.1	PSBA	photosystem II reaction center protein A	plastid
ATCG01230.1	RPS12B, RPS12	ribosomal protein S12B	plastid

ATCG00130.1	ATPF	ATPase, F0 complex, subunit B/B, bacterial/chloroplast	plastid
ATCG00150.1	ATPI	ATPase, F0 complex, subunit A protein	plastid
ATCG00160.1	RPS2	ribosomal protein S2	plastid
ATCG00270.1	PSBD	photosystem II reaction center protein D	plastid
ATCG00280.1	PSBC	photosystem II reaction center protein C	plastid
ATCG00330.1	RPS14	chloroplast ribosomal protein S14	plastid
ATCG00340.1	PSAB	Photosystem I, PsaA/PsaB protein	plastid
ATCG00350.1	PSAA	Photosystem I, PsaA/PsaB protein	plastid
ATCG00380.1	RPS4	chloroplast ribosomal protein S4	plastid
ATCG00420.1	NDHJ	NADH dehydrogenase subunit J	plastid
ATCG00430.1	PSBG	photosystem II reaction center protein G	plastid
ATCG00470.1	ATPE	ATP synthase epsilon chain	plastid
ATCG00490.1	RBCL	ribulose-bisphosphate carboxylases	plastid
ATCG00500.1	ACCD	acetyl-CoA carboxylase carboxyl transferase subunit beta	plastid
ATCG00520.1	YCF4	unfolded protein binding	plastid
ATCG00540.1	PETA	photosynthetic electron transfer A	plastid
ATCG00560.1	PSBL	photosystem II reaction center protein L	plastid
ATCG00570.1	PSBF	photosystem II reaction center protein F	plastid
ATCG00580.1	PSBE	photosystem II reaction center protein E	plastid
ATCG00650.1	RPS18	ribosomal protein S18	plastid
ATCG00680.1	PSBB	photosystem II reaction center protein B	plastid
ATCG00710.1	PSBH	photosystem II reaction center protein H	plastid
ATCG00720.1	PETB	photosynthetic electron transfer B	plastid
ATCG00730.1	PETD	photosynthetic electron transfer D	plastid
ATCG00750.1	RPS11	ribosomal protein S11	plastid
ATCG00770.1	RPS8	ribosomal protein S8	plastid
ATCG00780.1	RPL14	ribosomal protein L14	plastid
ATCG00790.1	RPL16	ribosomal protein L16	plastid
ATCG00800.1	structural constituent of ribosome	structural constituent of ribosome	plastid
ATCG00810.1	RPL22	ribosomal protein L22	plastid
ATCG01310.1	RPL2.2	ribosomal protein L2	plastid
ATCG01300.1	RPL23.2	ribosomal protein L23	plastid
ATCG01240.1	RPS7.2	ribosomal protein S7	plastid

ATCG01010.1	NDHF	NADH-Ubiquinone oxidoreductase (complex I), chain 5 protein	plastid
ATCG01050.1	NDHD	NADH-Ubiquinone/plastoquinone (complex I) protein	plastid
ATCG01060.1	PSAC	iron-sulfur cluster binding;electron carriers;4 iron, 4 sulfur cluster binding	plastid
ATCG01070.1	NDHE	NADH-ubiquinone/plastoquinone oxidoreductase chain 4L	plastid
ATCG01090.1	NDHI	NADPH dehydrogenases	plastid
ATCG01100.1	NDHA	NADH dehydrogenase family protein	plastid
ATCG01110.1	NDHH	NAD(P)H dehydrogenase subunit H	plastid
ATCG01120.1	RPS15	chloroplast ribosomal protein S15	plastid
ATCG01130.1	YCF1.2	Ycf1 protein	plastid

Supplementary Table 3 Subunits used for quantification of thylakoid protein complex abundance

Protein Complex	Accession	Symbol	Name
Photosystem I	AT3G16140.1	PSAH-1	photosystem I subunit H-1
Photosystem I	AT2G20260.1	PSAE-2	photosystem I subunit E-2
Photosystem I	AT4G02770.1	PSAD-1	photosystem I subunit D-1
Photosystem I	AT4G12800.1	PSAL	photosystem I subunit l
Photosystem I	AT4G28750.1	PSAE-1	Photosystem I reaction centre subunit IV / PsaE protein
Photosystem I	AT5G64040.1	PSAN	photosystem I reaction center subunit PSI-N, chloroplast, putative / PSI-N, putative (PSAN)
Photosystem I	ATCG00340.1	PSAB	Photosystem I, PsaA/PsaB protein
Photosystem I	ATCG00350.1	PSAA	Photosystem I, PsaA/PsaB protein
Photosystem I	ATCG01060.1	PSAC	iron-sulfur cluster binding;electron carriers;4 iron, 4 sulfur cluster binding
Photosystem I	AT1G55670.1	PSAG	photosystem I subunit G
Photosystem I	AT1G31330.1	PSAF	photosystem I subunit F
Photosystem I	AT1G30380.1	PSAK	photosystem I subunit K
Photosystem II	ATCG00560.1	PSBL	photosystem II reaction center protein L
Photosystem II	AT3G21055.1	PSBTN	photosystem II subunit T
Photosystem II	AT3G50820.1	PSBO2, PSBO-2, OEC33	photosystem II subunit O-2
Photosystem II	AT4G05180.1	PSBQ, PSBQ-2, PSII-Q	photosystem II subunit Q-2
Photosystem II	AT4G21280.1	PSBQ, PSBQA, PSBQ-1	photosystem II subunit QA
Photosystem II	AT5G66570.1	PSBO-1, OEE1, OEE33, OE33, PSBO1, MSP-1	PS II oxygen-evolving complex 1
Photosystem II	ATCG00020.1	PSBA	photosystem II reaction center protein A
Photosystem II	ATCG00270.1	PSBD	photosystem II reaction center protein D
Photosystem II	ATCG00280.1	PSBC	photosystem II reaction center protein C
Photosystem II	ATCG00570.1	PSBF	photosystem II reaction center protein F
Photosystem II	ATCG00580.1	PSBE	photosystem II reaction center protein E
Photosystem II	ATCG00680.1	PSBB	photosystem II reaction center protein B
Photosystem II	ATCG00710.1	PSBH	photosystem II reaction center protein H
Photosystem II	AT1G79040.1	PSBR	photosystem II subunit R
Photosystem II	AT1G06680.2	PSBP-1, OEE2, PSII-P	photosystem II subunit P-1
Cytochrome b6f	AT4G03280.2	PETC, PGR1	photosynthetic electron transfer C
Cytochrome b6f	ATCG00540.1	PETA	photosynthetic electron transfer A
Cytochrome b6f	ATCG00720.1	PETB	photosynthetic electron transfer B
Cytochrome b6f	ATCG00730.1	PETD	photosynthetic electron transfer D
ATP Synthase	ATCG00470.1	ATPE	ATP synthase epsilon chain

ATP Synthase	AT4G09650.1	ATPD	ATP synthase delta-subunit gene
ATP Synthase	ATCG00150.1	ATPI	ATPase, F0 complex, subunit A protein
ATP Synthase	ATCG00130.1	ATPF	ATPase, F0 complex, subunit B/B, bacterial/chloroplast
ATP Synthase	AT4G04640.1	ATPC1	ATPase, F1 complex, gamma subunit protein
ATP Synthase	ATCG00120.1	ATPA	ATP synthase subunit alpha
ATP Synthase	ATCG00480.1	ATPB	ATP synthase subunit beta
NAD(P)H Dehydrogenase	ATCG00420.1	NDHJ	NADH dehydrogenase subunit J
NAD(P)H Dehydrogenase	ATCG01070.1	NDHE	NADH-ubiquinone/plastoquinone oxidoreductase chain 4L
NAD(P)H Dehydrogenase	ATCG01090.1	NDHI	NADPH dehydrogenases
NAD(P)H Dehydrogenase	ATCG01100.1	NDHA	NADH dehydrogenase family protein
NAD(P)H Dehydrogenase	ATCG00430.1	PSBG	photosystem II reaction center protein G
NAD(P)H Dehydrogenase	ATCG01050.1	NDHD	NADH-Ubiquinone/plastoquinone (complex I) protein
NAD(P)H Dehydrogenase	AT5G21430.1	NDHU	Chaperone DnaJ-domain superfamily protein
NAD(P)H Dehydrogenase	AT2G39470.1	PPL2	PsbP-like protein 2
NAD(P)H Dehydrogenase	AT1G14150.1	PQL1, PQL2	PsbQ-like 2
NAD(P)H Dehydrogenase	AT3G01440.1	PQL1, PQL2	PsbQ-like 1
NAD(P)H Dehydrogenase	AT4G39710.1	FKBP16-2	FK506-binding protein 16-2
NAD(P)H Dehydrogenase	AT5G13120.2	CYP20-2	cyclophilin 20-2
NAD(P)H Dehydrogenase	AT1G15980.1	NDF1, NDH48	NDH-dependent cyclic electron flow 1
NAD(P)H Dehydrogenase	AT1G64770.1	NDF2, NDH45	NDH-dependent cyclic electron flow 1
NAD(P)H Dehydrogenase	AT4G37925.1	NDH-M	subunit NDH-M of NAD(P)H:plastoquinone dehydrogenase complex
NAD(P)H Dehydrogenase	AT5G58260.1	NDH-N	oxidoreductases, acting on NADH or NADPH, quinone or similar compound as acceptor
NAD(P)H Dehydrogenase	AT4G23890.1	NDHS	unknown protein; FUNCTIONS IN: molecular_function unknown; INVOLVED IN: biological_process unknown; LOCATED IN: chloroplast thylakoid membrane, chloroplast; EXPRESSED IN: 22 plant structures; EXPRESSED DURING: 13 growth stages; CO
NAD(P)H Dehydrogenase	ATCG01110.1	NDHH	NAD(P)H dehydrogenase subunit H
NAD(P)H Dehydrogenase	ATCG01010.1	NDHF	NADH-Ubiquinone oxidoreductase (complex I), chain 5 protein

Supplementary Table 4 Stoichiometries of major thylakoid protein complexes in wild type and *csk ko*

**WT-PSI Light**					
	**PSII**	**PSI**	**Cyt b_6f**	**ATPase**	**NDH**
**PSII**	1 ± 0	2.8 ± 0.08	3.9 ± 0.07	2.04 ± 0.05	100.89 ± 1.11
**PSI**	0.36 ± 0.01	1 ± 0	1.4 ± 0.02	0.73 ± 0.04	36.11 ± 0.61
**Cyt b_6f**	0.26 ± 0	0.72 ± 0.01	1 ± 0	0.52 ± 0.02	25.85 ± 0.21
**ATPase**	0.49 ± 0.01	1.37 ± 0.07	1.92 ± 0.08	1 ± 0	49.55 ± 1.67
**NDH**	0.01 ± 0	0.03 ± 0	0.04 ± 0	0.02 ± 0	1 ± 0

***csk*-PSI Light**					
	**PSII**	**PSI**	**Cyt b_6f**	**ATPase**	**NDH**
**PSII**	1 ± 0	2.83 ± 0.07	3.57 ± 0.01	2.1 ± 0.06	99.51 ± 3.21
**PSI**	0.35 ± 0.01	1 ± 0	1.26 ± 0.03	0.74 ± 0.01	35.14 ± 0.56
**Cyt b_6f**	0.28 ± 0	0.79 ± 0.02	1 ± 0	0.59 ± 0.02	27.89 ± 0.87
**ATPase**	0.48 ± 0.01	1.35 ± 0.01	1.7 ± 0.05	1 ± 0	47.39 ± 1.15
**NDH**	0.01 ± 0	0.03 ± 0	0.04 ± 0	0.02 ± 0	1 ± 0

**WT-PSII Light**					
	**PSII**	**PSI**	**Cyt b_6f**	**ATPase**	**NDH**
**PSII**	1 ± 0	1.58 ± 0.09	3.45 ± 0.12	2.12 ± 0.16	96.22 ± 2.2
**PSI**	0.59 ± 0.09	0.91 ± 0.09	2.02 ± 0.3	1.22 ± 0.11	56.05 ± 7.49
**Cyt b_6f**	0.29 ± 0.01	0.46 ± 0.03	1 ± 0	0.62 ± 0.05	27.92 ± 0.78
**ATPase**	0.47 ± 0.04	0.74 ± 0.02	1.63 ± 0.13	0.99 ± 0.01	45.31 ± 2.65
**NDH**	0.01 ± 0	0.02 ± 0	0.04 ± 0	0.02 ± 0	1 ± 0

***csk*-PSII Light**					
	**PSII**	**PSI**	**Cyt b_6f**	**ATPase**	**NDH**
**PSII**	1 ± 0	1.62 ± 0.04	3.62 ± 0.07	2.3 ± 0.02	101.09 ± 2.76
**PSI**	0.62 ± 0.02	1 ± 0	2.24 ± 0.07	1.42 ± 0.05	62.36 ± 1.28
**Cyt b_6f**	0.28 ± 0.01	0.45 ± 0.01	1 ± 0	0.64 ± 0.02	27.95 ± 1.16
**ATPase**	0.43 ± 0	0.7 ± 0.02	1.57 ± 0.05	1 ± 0	43.89 ± 1.18
**NDH**	0.01 ± 0	0.02 ± 0	0.04 ± 0	0.02 ± 0	1 ± 0

Supplementary Table 5 p-values for data contained in Figure 2B-P

t.test Wild type PSI vs PSII light (a)

Gene	p-value	p-value summary	Significance ($p < 0.05$)
psaA	0.001	***	Yes
psbA	0.029	*	Yes
psbB	0.003	**	Yes
psbD	0.048	*	Yes
petB	0.006	**	Yes
ndhB	0.004	**	Yes
atpB	0.007	**	Yes
SIG1	0.008	**	Yes
SIG2	0.003	**	Yes
SIG3	0.075	ns	No
SIG4	0.186	ns	No
SIG5	0.009	**	Yes
SIG6	0.001	***	Yes
psaA:*psbA*	0.030	*	Yes
psaA:*psbD*	0.025	*	Yes

t.test *phyB* PSI vs PSII light (b)

Gene	p-value	p-value summary	Significance ($p < 0.05$)
psaA	0.0110	*	Yes
psbA	0.0230	*	Yes
psbB	0.0153	*	Yes
psbD	0.2561	ns	No
petB	0.0288	*	Yes
ndhB	0.0102	*	Yes
atpB	0.0487	*	Yes
SIG1	0.2298	ns	No
SIG2	0.0749	ns	No
SIG3	0.0832	ns	No
SIG4	0.0646	ns	No
SIG5	0.0880	ns	No
SIG6	0.1299	ns	No
psaA:*psbA*	0.144	ns	No
psaA:*psbD*	0.042	*	Yes

t.test Wild type vs *phyB* PSI light (c)

Gene	p-value	p-value summary	Significance ($p < 0.05$)
psaA	0.011	*	Yes
psbA	0.010	**	Yes
psbB	0.035	*	Yes

psbD	0.405	ns	No
petB	0.241	ns	No
ndhB	0.008	**	Yes
atpB	0.025	*	Yes
SIG1	0.008	**	Yes
SIG2	0.033	*	Yes
SIG3	0.005	**	Yes
SIG4	< 0.0001	****	Yes
SIG5	0.003	**	Yes
SIG6	0.624	ns	No

t.test Wild type vs *phyB* PSII light (d)

Gene	p-value	p-value summary	Significance ($p < 0.05$)
psaA	0.001	***	Yes
psbA	0.054	ns	No
psbB	0.0005	***	Yes
psbD	0.108	ns	No
petB	0.029	*	Yes
ndhB	0.025	*	Yes
atpB	0.121	ns	No
SIG1	0.001	***	Yes
SIG2	0.003	**	Yes
SIG3	0.007	**	Yes
SIG4	0.003	**	Yes
SIG5	0.016	*	Yes
SIG6	0.452	ns	No

t.test Wild type vs *phyB* white light (e)

Gene	p-value	p-value summary	Significance ($p < 0.05$)
psaA	0.062	ns	No
psbA	0.064	ns	No
psbB	0.648	ns	No
psbD	0.255	ns	No
petB	0.022	*	Yes
ndhB	0.244	ns	No
atpB	0.509	ns	No
SIG1	0.025	*	Yes
SIG2	0.088	ns	No
SIG3	0.095	ns	No
SIG4	0.012	*	Yes
SIG5	0.454	ns	No
SIG6	0.0001	****	Yes

Supplementary Table 6 Up-regulated proteins in *phyB* vs wild type thylakoid proteome comparison

Light Conditions	Accession	Symbol	Name
PSI Light; PSII Light; White Light	AT2G35490.1		Plastid-lipid associated protein PAP / fibrillin family protein
PSI Light; PSII Light; White Light	AT3G52150.2		RNA-binding (RRM/RBD/RNP motifs) family protein
PSI Light; PSII Light; White Light	AT1G32220.1		NAD(P)-binding Rossmann-fold superfamily protein
PSI Light; PSII Light; White Light	AT3G10130.1		SOUL heme-binding family protein
PSI Light; PSII Light; White Light	AT4G35760.1		NAD(P)H dehydrogenase (quinone)s
PSI Light; PSII Light; White Light	AT5G11450.1		Mog1/PsbP/DUF1795-like photosystem II reaction center PsbP family protein
PSI Light; PSII Light; White Light	AT4G18370.1	DEG5, DEGP5, HHOA	DEGP protease 5
PSI Light; PSII Light; White Light	AT3G20390.1		endoribonuclease L-PSP family protein
PSI Light; PSII Light; White Light	AT4G19170.1	NCED4, CCD4	nine-cis-epoxycarotenoid dioxygenase 4
PSI Light; PSII Light; White Light	AT3G01480.1	CYP38, ATCYP38	cyclophilin 38
PSI Light; PSII Light; White Light	AT2G35410.1		RNA-binding (RRM/RBD/RNP motifs) family protein
PSI Light; PSII Light; White Light	AT1G75350.1	emb2184	Ribosomal protein L31
PSI Light; PSII Light; White Light	AT3G63190.1	RRF, HFP108, cpRRF, AtcpRRF	ribosome recycling factor, chloroplast precursor
PSI Light; White Light	AT2G25080.1	ATGPX1, GPX1	glutathione peroxidase 1
PSI Light; White Light	AT3G04870.2	ZDS, PDE181, SPC1	zeta-carotene desaturase
PSI Light; White Light	AT5G20720.3	CPN20	chaperonin 20

PSI Light; White Light	AT5G23120.1	HCF136	photosystem II stability/assembly factor, chloroplast (HCF136)
PSII Light; White Light	AT1G20340.1	DRT112, PETE2	Cupredoxin superfamily protein
PSII Light; White Light	AT4G13200.1		unknown protein;FUNCTIONS IN: molecular_function unknown;INVOLVED IN: biological_process unknown;LOCATED IN: thylakoid, chloroplast thylakoid membrane, chloroplast, plastoglobule;EXPRESSED IN: 22 plant structures;EXPRESSED DU
PSII Light; White Light	AT4G04020.1	FIB	fibrillin
PSII Light; White Light	AT1G64970.1	G-TMT, TMT1, VTE4	gamma-tocopherol methyltransferase
PSII Light; White Light	AT5G39830.2	DEGP8, DEG8	Trypsin family protein with PDZ domain
PSI Light; PSII Light	AT3G06510.1	SFR2, ATSFR2	Glycosyl hydrolase superfamily protein
PSI Light; PSII Light	AT1G51400.1		Photosystem II 5 kD protein
PSI Light; PSII Light	AT4G26530.1		Aldolase superfamily protein
PSI Light; PSII Light	ATCG01300.1	RPL23.2	ribosomal protein L23
PSI Light; PSII Light	ATCG00560.1	PSBL	photosystem II reaction center protein L
PSI Light; PSII Light	AT2G37660.1		NAD(P)-binding Rossmann-fold superfamily protein
PSI Light; PSII Light	AT3G62030.3	ROC4	rotamase CYP 4
PSI Light; PSII Light	AT5G48300.1	ADG1, APS1	ADP glucose pyrophosphorylase 1
PSI Light; PSII Light	AT5G58330.3		lactate/malate dehydrogenase family protein
PSI Light; PSII Light	AT3G55800.1	SBPASE	sedoheptulose-bisphosphatase
PSI Light; PSII Light	AT4G02770.1	PSAD-1	photosystem I subunit D-1
PSI Light; PSII Light	AT1G30380.1	PSAK	photosystem I subunit K
PSI Light; PSII Light	AT5G38420.1		Ribulose bisphosphate carboxylase (small chain) family protein
PSI Light; PSII Light	AT4G39730.1		Lipase/lipooxygenase, PLAT/LH2 family protein
PSI Light; PSII Light	AT3G63160.1		FUNCTIONS IN: molecular_function unknown;INVOLVED IN: biological_process unknown;LOCATED IN: chloroplast outer membrane, thylakoid, chloroplast thylakoid membrane, chloroplast, chloroplast envelope;EXPRESSED IN: 21 plant struct
PSI Light; PSII Light	AT3G43520.1		Transmembrane proteins 14C
PSI Light; PSII Light	AT2G21530.1		SMAD/FHA domain-containing protein

PSI Light; PSII Light	AT2G43030.1		Ribosomal protein L3 family protein
PSI Light; PSII Light	AT5G02940.1		Protein of unknown function (DUF1012)
PSI Light; PSII Light	AT1G16880.1		uridylyltransferase-related
PSI Light; PSII Light	AT2G28900.1	OEP16, ATOEP16-L, ATOEP16-1, OEP16-1	outer plastid envelope protein 16-1
PSI Light; PSII Light	AT3G21055.1	PSBTN	photosystem II subunit T
PSI Light; PSII Light	AT3G15110.1		unknown protein;FUNCTIONS IN: molecular_function unknown;INVOLVED IN: biological_process unknown;LOCATED IN: chloroplast thylakoid membrane;EXPRESSED IN: 20 plant structures;EXPRESSED DURING: 13 growth stages;CONTAINS InterP
PSI Light; PSII Light	AT5G28750.1		Bacterial sec-independent translocation protein mttA/Hcf106
PSI Light; PSII Light	AT5G64290.1	DCT, DIT2.1	dicarboxylate transport 2.1
PSI Light; PSII Light	AT1G32060.1	PRK	phosphoribulokinase
PSI Light; PSII Light	AT4G39460.1	SAMC1, SAMT1	S-adenosylmethionine carrier 1
PSI Light; PSII Light	AT1G03600.1	PSB27	photosystem II family protein
PSI Light; PSII Light	AT5G49910.1	CPHSC70-2EAT SHOCK PROTEIN 70-2, HSC70-7, cpHsc70-2	chloroplast heat shock protein 70-2
PSI Light; PSII Light	AT4G24280.1	cpHsc70-1	chloroplast heat shock protein 70-1
PSI Light; PSII Light	AT3G17970.1	atToc64-III, TOC64-III	translocon at the outer membrane of chloroplasts 64-III
PSI Light; PSII Light	AT3G52960.1		Thioredoxin superfamily protein
PSI Light; PSII Light	AT3G25920.1	RPL15	ribosomal protein L15
PSI Light; PSII Light	AT5G30510.1	RPS1, ARRPS1	ribosomal protein S1
PSI Light; PSII Light	AT4G22240.1		Plastid-lipid associated protein PAP / fibrillin family protein
PSI Light; PSII Light	AT4G28750.1	PSAE-1	Photosystem I reaction centre subunit IV / PsaE protein
PSI Light; PSII Light	AT2G24820.1	TIC55-II	translocon at the inner envelope membrane of chloroplasts 55-II
PSI Light; PSII Light	AT5G35170.2		adenylate kinase family protein
PSI Light; PSII Light	AT1G42970.1	GAPB	glyceraldehyde-3-phosphate dehydrogenase B subunit

PSI Light; PSII Light	AT2G28000.1	CPN60A, CH-CPN60A, SLP	chaperonin-60alpha
PSI Light; PSII Light	AT5G65220.1		Ribosomal L29 family protein
PSI Light; PSII Light	AT1G12250.2		Pentapeptide repeat-containing protein
PSI Light; PSII Light	AT3G52230.1		unknown protein;FUNCTIONS IN: molecular_function unknown;INVOLVED IN: biological_process unknown;LOCATED IN: chloroplast outer membrane, chloroplast thylakoid membrane, chloroplast, chloroplast envelope;EXPRESSED IN: 24 plant
PSI Light; PSII Light	AT3G01500.1	CA1, ATBCA1, SABP3, ATSABP3	carbonic anhydrase 1
PSI Light; PSII Light	AT5G14740.2	CA2, CA18, BETA CA2	carbonic anhydrase 2
PSI Light; PSII Light	AT2G47730.1	ATGSTF8, ATGSTF5, GST6, GSTF8	glutathione S-transferase phi 8
PSI Light; PSII Light	AT5G33320.1	CUE1, PPT, ARAPPT	Glucose-6-phosphate/phosphate translocator-related
PSI Light; PSII Light	AT3G45780.2	PHOT1, NPH1, JK224, RPT1	phototropin 1
PSI Light; PSII Light	AT5G06290.1	2-Cys Prx B, 2CPB	2-cysteine peroxiredoxin B
PSI Light; PSII Light	AT1G55670.1	PSAG	photosystem I subunit G
PSI Light; PSII Light	AT3G27850.1	RPL12-C	ribosomal protein L12-C
PSI Light; PSII Light	AT4G28660.1	PSB28	photosystem II reaction center PSB28 protein
PSI Light; PSII Light	ATCG00780.1	RPL14	ribosomal protein L14
PSI Light; PSII Light	AT5G16660.2		unknown protein;FUNCTIONS IN: molecular_function unknown;INVOLVED IN: biological_process unknown;LOCATED IN: chloroplast, membrane;EXPRESSED IN: 23 plant structures;EXPRESSED DURING: 14 growth stages;BEST Arabidopsis thalian
PSI Light; PSII Light	AT1G12900.4	GAPA-2	glyceraldehyde 3-phosphate dehydrogenase A subunit 2
PSI Light; PSII Light	AT4G24770.1	RBP31, ATRBP31, CP31, ATRBP33	31-kDa RNA binding protein
PSI Light; PSII Light	AT5G23890.1		LOCATED IN: mitochondrion, chloroplast thylakoid membrane, chloroplast, plastid, chloroplast envelope;EXPRESSED IN: 24 plant structures;EXPRESSED DURING: 14 growth stages;CONTAINS InterPro DOMAIN/s: S-layer homology domain (Int
PSI Light; PSII Light	AT3G25690.1	CHUP1	Hydroxyproline-rich glycoprotein family protein
PSI Light; PSII Light	AT3G13120.2		Ribosomal protein S10p/S20e family protein

PSI Light; PSII Light	AT5G55280.1	FTSZ1-1, ATFTSZ1-1, CPFTSZ	homolog of bacterial cytokinesis Z-ring protein FTSZ 1-1
PSI Light; PSII Light	AT3G11630.1		Thioredoxin superfamily protein
PSI Light; PSII Light	AT5G08540.1		unknown protein;FUNCTIONS IN: molecular_function unknown;INVOLVED IN: biological_process unknown;LOCATED IN: chloroplast thylakoid membrane, chloroplast, chloroplast envelope;EXPRESSED IN: 24 plant structures;EXPRESSED DURING
PSI Light; PSII Light	AT3G52380.1	CP33, PDE322	chloroplast RNA-binding protein 33
PSI Light; PSII Light	AT3G56910.1	PSRP5	plastid-specific 50S ribosomal protein 5
PSI Light; PSII Light	AT5G16620.1	PDE120, TIC40, ATTIC40	hydroxyproline-rich glycoprotein family protein
White Light	AT4G32770.1	VTE1, ATSDX1	tocopherol cyclase, chloroplast / vitamin E deficient 1 (VTE1) / sucrose export defective 1 (SXD1)
White Light	AT1G64355.1		unknown protein;FUNCTIONS IN: molecular_function unknown;INVOLVED IN: biological_process unknown;LOCATED IN: chloroplast;EXPRESSED IN: 23 plant structures;EXPRESSED DURING: 13 growth stages;CONTAINS InterPro DOMAIN/s: Protei
White Light	AT1G79600.1		Protein kinase superfamily protein
White Light	AT2G07698.1		ATPase, F1 complex, alpha subunit protein
White Light	AT1G78140.1		S-adenosyl-L-methionine-dependent methyltransferases superfamily protein
White Light	AT3G15360.1	ATHM4, TRX-M4, ATM4	thioredoxin M-type 4
White Light	AT1G03160.1	FZL	FZO-like
White Light	AT3G26060.1	ATPRX Q	Thioredoxin superfamily protein
White Light	AT1G06680.2	PSBP-1, OEE2, PSII-P	photosystem II subunit P-1
White Light	AT4G21280.1	PSBQ, PSBQA, PSBQ-1	photosystem II subunit QA
White Light	AT1G74070.1		Cyclophilin-like peptidyl-prolyl cis-trans isomerase family protein
White Light	AT1G16720.1	HCF173	high chlorophyll fluorescence phenotype 173
White Light	AT1G76450.1		Photosystem II reaction center PsbP family protein
White Light	AT5G67030.1	ABA1, LOS6, NPQ2, ATABA1, ZEP, IBS3, ATZEP	zeaxanthin epoxidase (ZEP) (ABA1)
White Light	AT3G15520.1		Cyclophilin-like peptidyl-prolyl cis-trans isomerase family protein
White Light	AT3G46780.1	PTAC16	plastid transcriptionally active 16
White Light	AT4G09010.1	APX4, TL29	ascorbate peroxidase 4
White Light	AT2G20890.1	PSB29, THF1	photosystem II reaction center PSB29 protein
White Light	AT5G55220.1		trigger factor type chaperone family protein
White Light	AT4G39960.1		Molecular chaperone Hsp40/DnaJ family protein

White Light	AT5G35100.1		Cyclophilin-like peptidyl-prolyl cis-trans isomerase family protein
White Light	AT4G33500.1		Protein phosphatase 2C family protein
White Light	AT1G17220.1	FUG1	Translation initiation factor 2, small GTP-binding protein
White Light	AT1G20020.1	ATLFNR2, FNR2	ferredoxin-NADP(+)-oxidoreductase 2
White Light	AT5G08740.1	NDC1	NAD(P)H dehydrogenase C1
White Light	AT5G04900.1	NOL	NYC1-like
White Light	AT1G71810.1		Protein kinase superfamily protein
White Light	AT2G21330.1	FBA1	fructose-bisphosphate aldolase 1
White Light	AT3G55330.1	PPL1	PsbP-like protein 1
White Light	ATCG01070.1	NDHE	NADH-ubiquinone/plastoquinone oxidoreductase chain 4L
White Light	AT1G01080.1		RNA-binding (RRM/RBD/RNP motifs) family protein
White Light	AT4G29060.1	emb2726	elongation factor Ts family protein
White Light	AT5G66190.1	ATLFNR1, FNR1	ferredoxin-NADP(+)-oxidoreductase 1
White Light	AT3G10060.1		FKBP-like peptidyl-prolyl cis-trans isomerase family protein
White Light	AT3G56650.1		Mog1/PsbP/DUF1795-like photosystem II reaction center PsbP family protein
White Light	AT3G01440.1	PQL1, PQL2	PsbQ-like 1
White Light	AT1G64770.1	NDF2, NDH45	NDH-dependent cyclic electron flow 1
White Light	AT5G13410.1		FKBP-like peptidyl-prolyl cis-trans isomerase family protein
White Light	AT3G58010.1	PGL34	plastoglobulin 34kD
White Light	AT2G30390.1	FC2, FC-II, ATFC-II	ferrochelatase 2
White Light	AT4G05180.1	PSBQ, PSBQ-2, PSII-Q	photosystem II subunit Q-2
White Light	AT4G09650.1	ATPD	ATP synthase delta-subunit gene
White Light	AT1G04620.1		coenzyme F420 hydrogenase family / dehydrogenase, beta subunit family
White Light	AT4G01310.1		Ribosomal L5P family protein
White Light	AT1G32990.1	PRPL11	plastid ribosomal protein l11
White Light	AT3G63140.1	CSP41A	chloroplast stem-loop binding protein of 41 kDa
White Light	AT3G15190.1		chloroplast 30S ribosomal protein S20, putative
White Light	AT3G12780.1	PGK1	phosphoglycerate kinase 1
White Light	AT1G55490.2	CPN60B, LEN1	chaperonin 60 beta
White Light	AT1G02560.1	CLPP5, NCLPP5, NCLPP1	nuclear encoded CLP protease 5
White Light	AT1G67090.1	RBCS1A	ribulose bisphosphate carboxylase small chain 1A
White Light	AT3G23400.1	FIB4	Plastid-lipid associated protein PAP / fibrillin family protein
White Light	AT5G54600.1		Translation protein SH3-like family protein
White Light	AT5G35630.3	GS2, GLN2, ATGSL1	glutamine synthetase 2
White Light	AT5G45390.1	CLPP4, NCLPP4	CLP protease P4
White Light	AT2G37220.1		RNA-binding (RRM/RBD/RNP motifs) family protein

White Light	ATCG01240.1	RPS7.2	ribosomal protein S7
White Light	AT2G33800.1		Ribosomal protein S5 family protein
White Light	AT3G60750.2		Transketolase
White Light	AT2G44640.1		FUNCTIONS IN: molecular_function unknown;INVOLVED IN: biological_process unknown;LOCATED IN: mitochondrion, chloroplast, plasma membrane, plastid, chloroplast envelope;EXPRESSED IN: 23 plant structures;EXPRESSED DURING: 13 gro
White Light	AT1G15820.1	LHCB6, CP24	light harvesting complex photosystem II subunit 6
White Light	ATCG00490.1	RBCL	ribulose-bisphosphate carboxylases
PSII Light	AT4G37200.1	HCF164	Thioredoxin superfamily protein
PSII Light	AT4G33350.1		Tic22-like family protein
PSII Light	AT4G02530.1		chloroplast thylakoid lumen protein
PSII Light	AT1G08550.2	NPQ1, AVDE1	non-photochemical quenching 1
PSII Light	AT1G50450.1		Saccharopine dehydrogenase
PSII Light	AT2G20260.1	PSAE-2	photosystem I subunit E-2
PSII Light	AT3G27925.1	DEGP1, Deg1	DegP protease 1
PSII Light	AT4G02510.1	TOC159, TOC86, PPI2, TOC160, ATTOC159	translocon at the outer envelope membrane of chloroplasts 159
PSII Light	AT1G42960.1		expressed protein localized to the inner membrane of the chloroplast.
PSII Light	AT3G27820.1	ATMDAR4, MDAR4	monodehydroascorbate reductase 4
PSII Light	AT4G03520.1	ATHM2	Thioredoxin superfamily protein
PSII Light	AT2G23670.1	YCF37	homolog of Synechocystis YCF37
PSII Light	AT5G42480.1	ARC6	Chaperone DnaJ-domain superfamily protein

Supplementary Table 7 Down-regulated proteins in *phyB* vs wild type thylakoid proteome comparison

Light Conditions	FASTA Header	Symbol	Name
PSI Light; PSII Light; White Light	AT4G14870.1	SECE1	secE/sec61-gamma protein transport protein
PSI Light; PSII Light; White Light	ATCG01120.1	RPS15	chloroplast ribosomal protein S15
PSI Light; PSII Light; White Light	ATCG01100.1	NDHA	NADH dehydrogenase family protein
PSI Light; PSII Light; White Light	AT4G24750.1		Rhodanese/Cell cycle control phosphatase superfamily protein
PSI Light; PSII Light; White Light	AT3G58610.3		ketol-acid reductoisomerase
PSI Light; PSII Light; White Light	AT5G24490.1		30S ribosomal protein, putative
PSI Light; White Light	AT5G46290.2	KASI, KAS1	3-ketoacyl-acyl carrier protein synthase I
PSII Light; White Light	AT3G19170.1	ATPREP1, ATZNMP, PREP1	presequence protease 1
PSI Light; PSII Light	ATCG00430.1	PSBG	photosystem II reaction center protein G
PSI Light; PSII Light	AT3G48500.1	PDE312, PTAC10	Nucleic acid-binding, OB-fold-like protein
PSI Light; PSII Light	AT3G04260.1	PTAC3	plastid transcriptionally active 3
PSI Light; PSII Light	AT1G31330.1	PSAF	photosystem I subunit F
PSI Light; PSII Light	AT1G03160.1	FZL	FZO-like
PSI Light; PSII Light	AT5G05740.3	EGY2	ethylene-dependent gravitropism-deficient and yellow-green-like 2
PSI Light; PSII Light	AT3G26060.1	ATPRX Q	Thioredoxin superfamily protein
PSI Light; PSII Light	AT5G44650.1	CEST, AtCEST	Encodes a chloroplast protein that induces tolerance to multiple environmental stresses and reduces photooxidative damage.
PSI Light; PSII Light	AT1G77490.1	TAPX	thylakoidal ascorbate peroxidase
PSI Light; PSII Light	AT3G26710.1	CCB1	cofactor assembly of complex C
PSI Light; PSII Light	AT5G58260.1		oxidoreductases, acting on NADH or NADPH, quinone or similar compound as acceptor

PSI Light; PSII Light	AT1G67700.2		unknown protein;FUNCTIONS IN: molecular_function unknown;INVOLVED IN: biological_process unknown;LOCATED IN: chloroplast, chloroplast envelope;EXPRESSED IN: 22 plant structures;EXPRESSED DURING: 13 growth stages;Has 49 Blast
PSI Light; PSII Light	AT5G51545.1	LPA2	low psii accumulation2
PSI Light; PSII Light	ATCG00720.1	PETB	photosynthetic electron transfer B
PSI Light; PSII Light	AT2G34420.1	LHB1B2, LHCB1.5	photosystem II light harvesting complex gene B1B2
PSI Light; PSII Light	AT1G74470.1		Pyridine nucleotide-disulphide oxidoreductase family protein
PSI Light; PSII Light	AT1G73110.1		P-loop containing nucleoside triphosphate hydrolases superfamily protein
PSI Light; PSII Light	AT2G05620.1	PGR5	proton gradient regulation 5
PSI Light; PSII Light	AT4G09010.1	APX4, TL29	ascorbate peroxidase 4
PSI Light; PSII Light	AT1G73990.1	SPPA, SPPA1	signal peptide peptidase
PSI Light; PSII Light	AT2G20890.1	PSB29, THF1	photosystem II reaction center PSB29 protein
PSI Light; PSII Light	ATCG01110.1	NDHH	NAD(P)H dehydrogenase subunit H
PSI Light; PSII Light	AT5G55220.1		trigger factor type chaperone family protein
PSI Light; PSII Light	AT5G18660.1	PCB2	NAD(P)-binding Rossmann-fold superfamily protein
PSI Light; PSII Light	AT4G14210.2	PDS3, PDS, PDE226	phytoene desaturase 3
PSI Light; PSII Light	AT1G74730.1		Protein of unknown function (DUF1118)
PSI Light; PSII Light	AT2G32640.1		Lycopene beta/epsilon cyclase protein
PSI Light; PSII Light	AT2G40100.1	LHCB4.3	light harvesting complex photosystem II
PSI Light; PSII Light	AT1G02910.1	LPA1	tetratricopeptide repeat (TPR)-containing protein
PSI Light; PSII Light	ATCG00680.1	PSBB	photosystem II reaction center protein B
PSI Light; PSII Light	AT3G12345.1		unknown protein;LOCATED IN: chloroplast;Has 35333 Blast hits to 34131 proteins in 2444 species: Archae - 798;Bacteria - 22429;Metazoa - 974;Fungi - 991;Plants - 531;Viruses - 0;Other Eukaryotes - 9610 (source: NCBI BLink).
PSI Light; PSII Light	AT3G47070.1		LOCATED IN: thylakoid, chloroplast thylakoid membrane, chloroplast, chloroplast envelope;EXPRESSED IN: 22 plant structures;EXPRESSED DURING: 13 growth stages;CONTAINS InterPro DOMAIN/s: Thylakoid soluble phosphoprotein TSP9 (In

PSI Light; PSII Light	AT2G24060.1		Translation initiation factor 3 protein
PSI Light; PSII Light	AT2G30950.1	VAR2, FTSH2	FtsH extracellular protease family
PSI Light; PSII Light	AT1G03630.2	POR C, PORC	protochlorophyllide oxidoreductase C
PSI Light; PSII Light	AT3G61470.1	LHCA2	photosystem I light harvesting complex gene 2
PSI Light; PSII Light	AT4G17600.1	LIL3:1	Chlorophyll A-B binding family protein
PSI Light; PSII Light	AT4G20130.1	PTAC14	plastid transcriptionally active 14
PSI Light; PSII Light	AT4G27440.2	PORB	protochlorophyllide oxidoreductase B
PSI Light; PSII Light	AT3G51820.1	ATG4, G4, CHLG	UbiA prenyltransferase family protein
PSI Light; PSII Light	AT1G73060.1	LPA3	Low PSII Accumulation 3
PSI Light; PSII Light	AT1G69200.1	FLN2	fructokinase-like 2
PSI Light; PSII Light	AT1G65230.1		Uncharacterized conserved protein (DUF2358)
PSI Light; PSII Light	AT2G42220.1		Rhodanese/Cell cycle control phosphatase superfamily protein
PSI Light; PSII Light	AT4G01690.1	PPOX, HEMG1, PPO1	Flavin containing amine oxidoreductase family
PSI Light; PSII Light	AT5G17170.1	ENH1	rubredoxin family protein
PSI Light; PSII Light	AT2G05070.1	LHCB2.2, LHCB2	photosystem II light harvesting complex gene 2.2
PSI Light; PSII Light	AT1G06190.1		Rho termination factor
PSI Light; PSII Light	AT1G54520.1		unknown protein;FUNCTIONS IN: molecular_function unknown;INVOLVED IN: biological_process unknown;LOCATED IN: chloroplast;EXPRESSED IN: 24 plant structures;EXPRESSED DURING: 15 growth stages;CONTAINS InterPro DOMAIN/s: Protei
PSI Light; PSII Light	ATCG00520.1	YCF4	unfolded protein binding
PSI Light; PSII Light	AT5G66190.1	ATLFNR1, FNR1	ferredoxin-NADP(+)-oxidoreductase 1
PSI Light; PSII Light	AT5G54270.1	LHCB3, LHCB3*1	light-harvesting chlorophyll B-binding protein 3
PSI Light; PSII Light	AT2G18710.1	SCY1	SECY homolog 1
PSI Light; PSII Light	AT4G27700.1		Rhodanese/Cell cycle control phosphatase superfamily protein
PSI Light; PSII Light	AT5G13120.2	CYP20-2	cyclophilin 20-2
PSI Light; PSII Light	ATCG01090.1	NDHI	NADPH dehydrogenases

PSI Light; PSII Light	AT5G47110.1	LIL3:2	Chlorophyll A-B binding family protein
PSI Light; PSII Light	AT5G57030.1	LUT2	Lycopene beta/epsilon cyclase protein
PSI Light; PSII Light	AT5G13410.1		FKBP-like peptidyl-prolyl cis-trans isomerase family protein
PSI Light; PSII Light	AT3G56940.1	CRD1, CHL27, ACSF	dicarboxylate diiron protein, putative (Crd1)
PSI Light; PSII Light	AT1G22700.3		Tetratricopeptide repeat (TPR)-like superfamily protein
PSI Light; PSII Light	AT2G30390.1	FC2, FC-II, ATFC-II	ferrochelatase 2
PSI Light; PSII Light	AT5G19940.1		Plastid-lipid associated protein PAP / fibrillin family protein
PSI Light; PSII Light	AT1G52510.1		alpha/beta-Hydrolases superfamily protein
PSI Light; PSII Light	ATCG00420.1	NDHJ	NADH dehydrogenase subunit J
PSI Light; PSII Light	AT1G50250.1	FTSH1	FTSH protease 1
PSI Light; PSII Light	ATCG00470.1	ATPE	ATP synthase epsilon chain
PSI Light; PSII Light	AT5G03940.1	FFC, 54CP, CPSRP54, SRP54CP	chloroplast signal recognition particle 54 kDa subunit
PSI Light; PSII Light	AT3G10690.1	GYRA	DNA GYRASE A
PSI Light; PSII Light	AT1G08520.1	ALB1, ALB-1V, V157, PDE166, CHLD	ALBINA 1
White Light	AT3G63140.1	CSP41A	chloroplast stem-loop binding protein of 41 kDa
White Light	AT4G26530.1		Aldolase superfamily protein
White Light	AT5G48300.1	ADG1, APS1	ADP glucose pyrophosphorylase 1
White Light	AT3G55800.1	SBPASE	sedoheptulose-bisphosphatase
White Light	AT3G12780.1	PGK1	phosphoglycerate kinase 1
White Light	AT5G38420.1		Ribulose bisphosphate carboxylase (small chain) family protein
White Light	AT1G55490.2	CPN60B, LEN1	chaperonin 60 beta
White Light	AT1G02560.1	CLPP5, NCLPP5, NCLPP1	nuclear encoded CLP protease 5
White Light	AT3G48730.1	GSA2	glutamate-1-semialdehyde 2,1-aminomutase 2
White Light	AT1G67090.1	RBCS1A	ribulose bisphosphate carboxylase small chain 1A
White Light	AT1G03475.1	LIN2, HEMF1, ATCPO-I	Coproporphyrinogen III oxidase
White Light	AT1G09340.1	CRB, CSP41B, HIP1.3	chloroplast RNA binding
White Light	AT1G32060.1	PRK	phosphoribulokinase
White Light	AT1G63940.4	MDAR6	monodehydroascorbate reductase 6
White Light	AT5G49910.1	CPHSC70-2EAT SHOCK PROTEIN 70-	chloroplast heat shock protein 70-2

		2, HSC70-7, cpHsc70-2	
White Light	AT4G24280.1	cpHsc70-1	chloroplast heat shock protein 70-1
White Light	AT5G35630.3	GS2, GLN2, ATGSL1	glutamine synthetase 2
White Light	AT5G45390.1	CLPP4, NCLPP4	CLP protease P4
White Light	AT3G52960.1		Thioredoxin superfamily protein
White Light	AT4G18480.1	CHLI1, CH42, CH-42, CHL11, CHLI-1	P-loop containing nucleoside triphosphate hydrolases superfamily protein
White Light	AT4G20360.1	ATRAB8D, ATRABE1B, RABE1b	RAB GTPase homolog E1B
White Light	AT5G35170.2		adenylate kinase family protein
White Light	AT3G26650.1	GAPA, GAPA-1	glyceraldehyde 3-phosphate dehydrogenase A subunit
White Light	AT1G42970.1	GAPB	glyceraldehyde-3-phosphate dehydrogenase B subunit
White Light	AT2G47730.1	ATGSTF8, ATGSTF5, GST6, GSTF8	glutathione S-transferase phi 8
White Light	AT1G11750.1	CLPP6, NCLPP1, NCLPP6	CLP protease proteolytic subunit 6
White Light	AT5G04140.1	GLU1, GLS1, GLUS, FD-GOGAT	glutamate synthase 1
White Light	AT3G45780.2	PHOT1, NPH1, JK224, RPT1	phototropin 1
White Light	AT1G62750.1	ATSCO1, ATSCO1/CPEF-G, SCO1	Translation elongation factor EFG/EF2 protein
White Light	AT3G60750.2		Transketolase
White Light	AT4G28660.1	PSB28	photosystem II reaction center PSB28 protein
White Light	AT1G12900.4	GAPA-2	glyceraldehyde 3-phosphate dehydrogenase A subunit 2
White Light	AT5G35360.1	CAC2	acetyl Co-enzyme a carboxylase biotin carboxylase subunit
White Light	ATCG00490.1	RBCL	ribulose-bisphosphate carboxylases
PSI Light	AT1G79600.1		Protein kinase superfamily protein
PSI Light	AT1G78140.1		S-adenosyl-L-methionine-dependent methyltransferases superfamily protein
PSI Light	AT4G18810.1		NAD(P)-binding Rossmann-fold superfamily protein
PSI Light	AT1G15980.1	NDF1, NDH48	NDH-dependent cyclic electron flow 1
PSI Light	AT3G18890.1		NAD(P)-binding Rossmann-fold superfamily protein
PSI Light	AT1G74070.1		Cyclophilin-like peptidyl-prolyl cis-trans isomerase family protein
PSI Light	AT1G72640.2		NAD(P)-binding Rossmann-fold superfamily protein
PSI Light	AT1G34430.1	EMB3003	2-oxoacid dehydrogenases acyltransferase family protein
PSI Light	AT4G34620.1	SSR16	small subunit ribosomal protein 16
PSI Light	AT4G33500.1		Protein phosphatase 2C family protein
PSI Light	AT5G08740.1	NDC1	NAD(P)H dehydrogenase C1
PSI Light	AT1G68830.1	STN7	STT7 homolog STN7
PSI Light	AT1G70760.1	CRR23	inorganic carbon transport protein-related

PSI Light	AT1G14345.1		NAD(P)-linked oxidoreductase superfamily protein
PSI Light	AT2G01110.1	APG2, UNE3, PGA2, TATC	Sec-independent periplasmic protein translocase
PSI Light	AT4G16155.1		dihydrolipoyl dehydrogenases
PSI Light	AT3G25860.1	LTA2, PLE2	2-oxoacid dehydrogenases acyltransferase family protein
PSI Light	AT3G16000.1	MFP1	MAR binding filament-like protein 1
PSI Light	AT2G21960.1		unknown protein;LOCATED IN: chloroplast;EXPRESSED IN: 22 plant structures;EXPRESSED DURING: 13 growth stages;BEST Arabidopsis thaliana protein match is: unknown protein (TAIR:AT1G56180.1);Has 224 Blast hits to 222 proteins in
PSI Light	AT1G04620.1		coenzyme F420 hydrogenase family / dehydrogenase, beta subunit family
PSII Light	AT5G26742.2	emb1138	DEAD box RNA helicase (RH3)
PSII Light	AT4G39960.1		Molecular chaperone Hsp40/DnaJ family protein
PSII Light	AT1G17220.1	FUG1	Translation initiation factor 2, small GTP-binding protein
PSII Light	AT2G21330.1	FBA1	fructose-bisphosphate aldolase 1
PSII Light	AT4G29060.1	emb2726	elongation factor Ts family protein
PSII Light	AT3G09580.1		FAD/NAD(P)-binding oxidoreductase family protein
PSII Light	AT3G47520.1	MDH	malate dehydrogenase
PSII Light	AT1G55480.1	ZKT	protein containing PDZ domain, a K-box domain, and a TPR region

Supplementary Table 8 Primer pairs used for qPCR analysis

Gene ID	Primer name	Sequence 5'-3'
psaA	PsaA-F	GGCACAAGCATCTCAGGTAA
	PsaA-R	AGCCCAAACAATGGATTCAA
psbA	PsbA-F	GGTTACAGATTCGGGCAAGA
	PsbA-R	AATACCTACTACCGGCCAAGC
psbB	PsbB-F	CTGCTCTAGTTGCTGGTTGG
	PsbB-R	CGCCCCAAGAATTGGTTATT
psbD	PsbD-F	CTTTAGGGGGTTGGTTCACA
	PsbD-R	GCTTCAGGACCCCACAGTAA
petB	PetB-F	ATTGGGCGGTCAAAATTGTA
	PetB-R	AGACGGCCGTAAGAAGAGGT
atpB	AtpB-F	ATGAGTGCGACAGAGGGTTT
	AtpB-R	TGTGCGAGTATCGACAGGAC
ndhB	NdhB-F	TACGAAGGATCTCCCACTCC
	NdhB-R	GCTAAGAATAGCTAGGATTTCCAGA
SIG1	SIG1-F	TCATAAGTCAAGATTGAAGGATAGATT
	SIG1-R	CAAACGCACATTACTCATAGCC
SIG2	SIG2-F	TGTGCCCCTAAACACAACAA
	SIG2-R	TGCCTGTCTGATATAAGGGAATC
SIG3	SIG3-F	TCCCAAACAGATTGTTGGTTC
	SIG3-R	CAATAACGAGAGCCAATGTTTA
SIG4	SIG4-F	TTGTACCTCAAGGAAGGAGCA
	SIG4-R	CTTTTCTCTCGCTTCTTTTCTACG
SIG5	SIG5-F	TGAAGGCTCTTCTTGAAGTGAA
	SIG5-R	TTAATAAGTTTGTTTCTTGCAGCTC
SIG6	SIG6-F	TCCTTCCTCTGTAGTGATGCTT
	SIG6-R	TCGTCTCTTAACGCATGTGA
rrn16	16S -F	CGGCTGCTAATACCCCGTAG
	16S -R	GTGGCTGATCATCCTCTCGG
